# Pharmacological suppression of the OTUD4/CD73 proteolytic axis revives antitumor immunity against immune-suppressive breast cancers

**DOI:** 10.1172/JCI176390

**Published:** 2024-03-26

**Authors:** Yueming Zhu, Anupam Banerjee, Ping Xie, Andrey A. Ivanov, Amad Uddin, Qiao Jiao, Junlong Jack Chi, Lidan Zeng, Ji Young Lee, Yifan Xue, Xinghua Lu, Massimo Cristofanilli, William J. Gradishar, Curtis J. Henry, Theresa W. Gillespie, Manali Ajay Bhave, Kevin Kalinsky, Haian Fu, Ivet Bahar, Bin Zhang, Yong Wan

**Affiliations:** 1 Department of Pharmacology and Chemical Biology and; 2 Winship Cancer Institute, Emory University School of Medicine, Atlanta, Georgia, USA.; 3 Laufer Center for Physical and Quantitative Biology, School of Medicine, Stony Brook University, Stony Brook, New York, USA.; 4 Department of Medicine, Robert H. Lurie Comprehensive Cancer Center, Northwestern University Feinberg School of Medicine, Chicago, Illinois, USA.; 5 Emory Chemical Biology Discovery Center, Emory University School of Medicine, Emory University, Atlanta, Georgia, USA.; 6 Driskill Graduate Program (DPG), Northwestern University Feinberg School of Medicine, Chicago, Illinois, USA.; 7 Department of Biomedical Informatics, University of Pittsburgh School of Medicine, Pittsburgh, Pennsylvania, USA.; 8 Department of Medicine, Weill Cornell Medicine, New York, New York, USA.; 9 Department of Pediatrics,; 10 Department of Surgery, and; 11 Department of Hematology and Medical Oncology, Emory University School of Medicine, Atlanta, Georgia, USA.; 12 Department of Biochemistry and Cell Biology, School of Medicine, Stony Brook University, Stony Brook, New York, USA.

**Keywords:** Oncology, Therapeutics, Cancer immunotherapy, Drug screens

## Abstract

Despite widespread utilization of immunotherapy, treating immune-cold tumors remains a challenge. Multiomic analyses and experimental validation identified the OTUD4/CD73 proteolytic axis as a promising target in treating immune-suppressive triple negative breast cancer (TNBC). Mechanistically, deubiquitylation of CD73 by OTUD4 counteracted its ubiquitylation by TRIM21, resulting in CD73 stabilization inhibiting tumor immune responses. We further demonstrated the importance of TGF-β signaling for orchestrating the OTUD4/CD73 proteolytic axis within tumor cells. Spatial transcriptomics profiling discovered spatially resolved features of interacting malignant and immune cells pertaining to expression levels of OTUD4 and CD73. In addition, ST80, a newly developed inhibitor, specifically disrupted proteolytic interaction between CD73 and OTUD4, leading to reinvigoration of cytotoxic CD8^+^ T cell activities. In preclinical models of TNBC, ST80 treatment sensitized refractory tumors to anti-PD-L1 therapy. Collectively, our findings uncover what we believe to be a novel strategy for targeting the immunosuppressive OTUD4/CD73 proteolytic axis in treating immune-suppressive breast cancers with the inhibitor ST80.

## Abstract 2

Pharmacological suppression of the OTUD4/CD73 proteolytic axis revives antitumor immunity against immune-suppressive breast cancers in mouse models.

## Graphical abstract

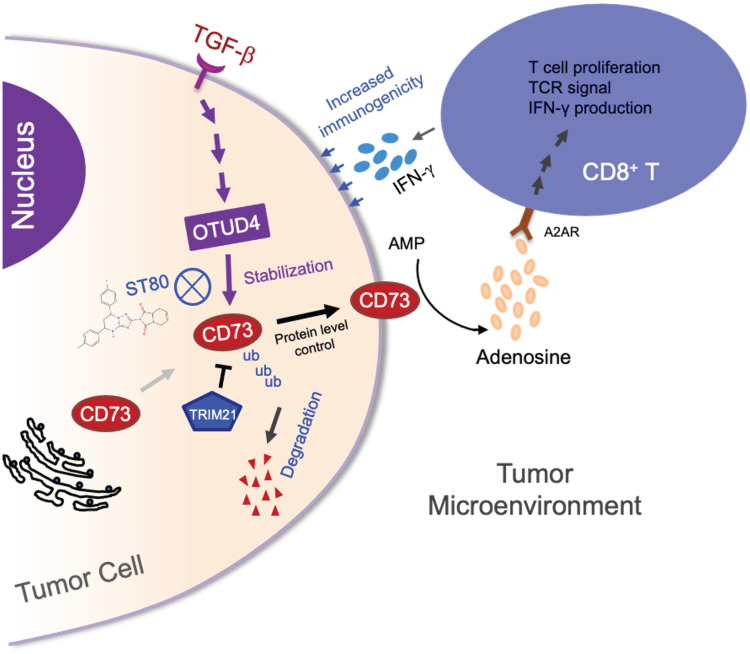

## Introduction

Despite the widespread utilization of immunotherapy, the poor clinical response by immune-suppressive tumors is an emerging challenge in use of current immune checkpoint inhibitors. Triple negative breast cancers (TNBCs) are the most aggressive mammary carcinoma subtype and have few effective targeted therapies. Although PD-L1 inhibitors demonstrated their promising clinical benefits in certain subsets of patients with TNBC, the intricacies of the tumor microenvironment, with its inherent immunosuppressive properties and varied immunogenicity profiles, limit the broad efficacy of such therapies (1–3). Breast cancer’s “immune-cold” property caused by a scarcity of T lymphocyte infiltration and expression of different immune checkpoint proteins has been proposed to be responsible for the decreased anticancer immunity (4–6). Our endeavor, based on multiomic analyses, to identify new targets for improving anticancer immune responses against TNBCs has drawn our attention to CD73 and OTUD4 (7–9).

CD73 or Ecto-5′-nucleotidase, a pivotal enzyme expressed in a range of tissues and cell types, acts together with CD39 in the conversion of extracellular ATP to immunosuppressive adenosine (10–13). This adenosinergic cascade leads to notable outcomes: the suppression of cytotoxic T cells and the creation of an immune-suppressive tumor microenvironment (7, 14). Further, CD73 is implicated in advancing tumor progression by facilitating tumor angiogenesis and interacting with cancer-associated fibroblasts through adenosine receptors such as A1R, A2AR, A2BR, and A3R vis-à-vis multiple types of immune cells such as Tregs (Foxp3^+^) T cells, effector T cells, natural killer (NK) cells, myeloid-derived suppressor cells (MDSCs), and macrophages, as well as B cells (9, 15). In the realm of breast cancer research, CD73 has attracted significant interest not only due to its association with poor prognosis but also its pronounced overexpression in TNBCs (16). While the regulation of CD73 expression by factors like HIF-1α and estrogen receptor (ER) has been documented, an intriguing yet underexplored dimension is the potential posttranslational modifications (PTMs) of CD73, especially given the well-established significance of PTMs in regulating other immune checkpoint proteins, such as PD-L1 and B7-H4 (4, 17).

OTUD4 (OTU domain-containing protein 4) acts as a deubiquitinase that counteracts E3 ligase–mediated protein ubiquitylation, stabilizing the targeted protein (18, 19). Malfunction of OTUD4 has been linked to abnormal antiviral immune response and inflammation through targeting MyD88, MAVS and TLRs (19). Moreover, the role of OTUD4 was thought to be involved in DNA repair by stabilizing ALKBH3 (18). Intriguingly, the function and regulation of OTUD4 have been subjected to TGF-β receptor signaling (20). In addition, aberrant accumulation of OTUD4 has been observed in various types of tumors and immune cells (8). Nevertheless, the impact of OTUD4 in regulating tumor immune response and tumor progression remains largely unknown.

In the current study, we have identified that the OTUD4/CD73 proteolytic axis is an ideal target in treating immune-suppressive TNBCs. We have demonstrated that, while E3 ligase TRIM21 catalyzes the ubiquitylation of CD73 for degradation, cleaving off the ubiquitin-conjugated chain from CD73 by OTUD4 results in the stabilization of CD73 that dampens CD8^+^ T cell function. We have further determined that the molecular axis OTUD4/CD73/adenosine is dictated by TGF-β signaling, whereas the TRIM21/CD73 axis is regulated in response to IFN-γ signaling. Clinically, we found that the OTUD4^lo^/CD73^lo^ signature in a subset of human breast malignancies was associated with a favorable immune profile. We further developed a pharmacological inhibitor, ST80, to resume CD73 proteolysis and restore capacity to elicit CD8^+^ interferon-γ-producing T cell responses through the blockade of the interaction between OTUD4 and CD73. Our findings uncover a novel strategy for targeting the immunosuppressive proteolytic axis OTUD4/CD73 in treating immune-suppressive TNBCs.

## Results

### Elevated expression of CD73 is dramatically associated with an unfavorable tumor immune response and prognosis in immune-suppressive TNBCs.

**Figure 1:**
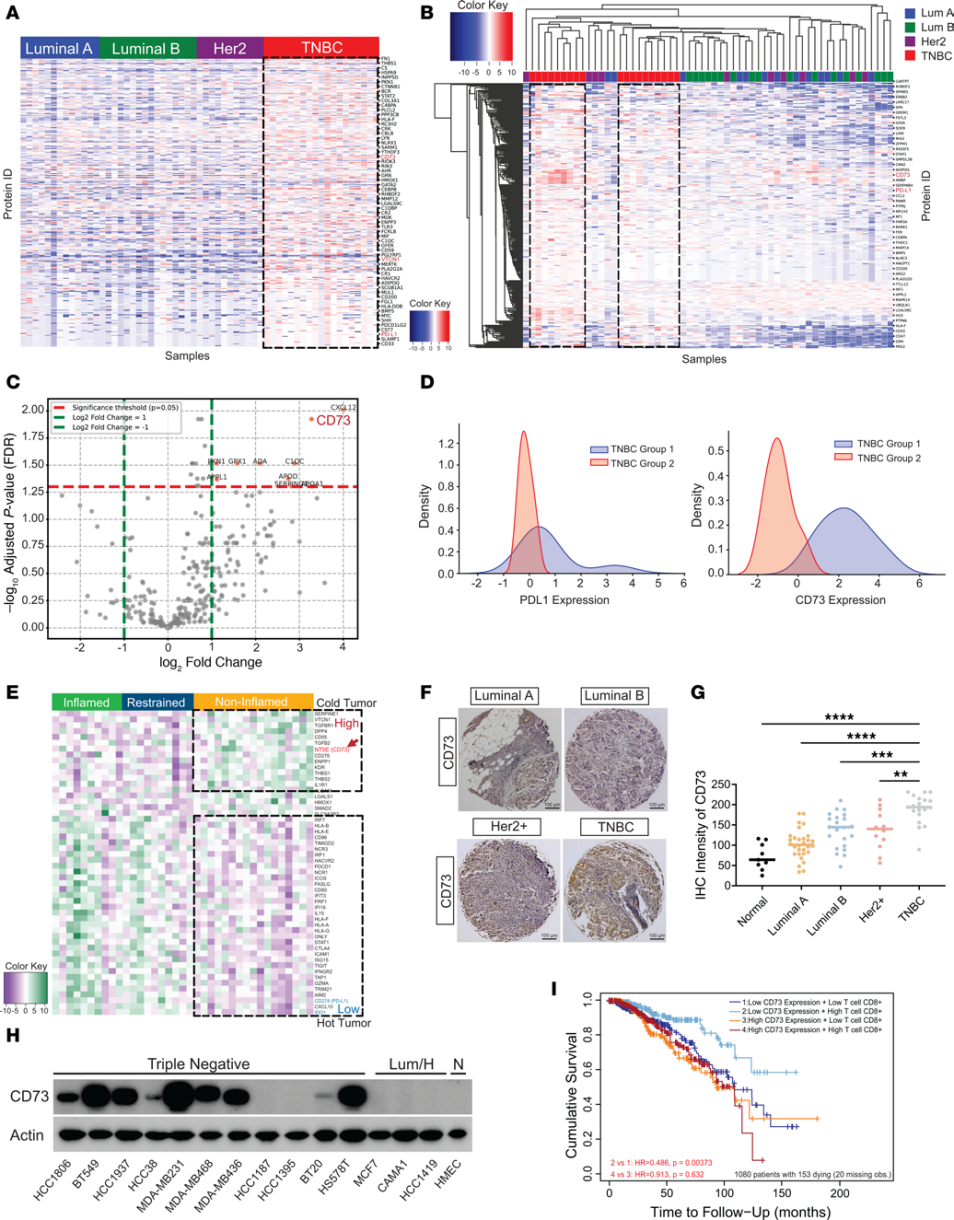
Accumulation of CD73 is dramatically associated with an unfavorable tumor immune response and prognosis in immune-suppressive breast cancers. (**A**) Proteomic analysis of 59 immune-related proteins in TCGA samples of PAM50-defined intrinsic and hormone receptor subtypes, including 19 TNBC, 13 luminal A, 17 luminal B, and 10 Her2^+^ breast tumors. (**B**) Heatmap and hierarchical clustering are based on the expression levels of the 309 immune-related proteins — differentially expressed in 4 breast cancer types. Each column represents a sample; each row represents a protein. The log_2_ relative protein expression scale is depicted on the top left. (**C**) Volcano plots showing the immune-related protein expression changes in 2 TNBC patient cohorts. Each circle represents 1 protein. The log fold change is represented on the x-axis. The y-axis shows the FDR adjusted log_10_ of the *P* value. (**D**) CD73 and PD-L1 expression density in 2 TNBC groups were calculated based on log_2_ relative protein expression. (**E**) mRNA analysis of immune-relevant proteins in 37 specimens from patients with TNBC from the GEO database. The genes (rows) are sorted according to the difference between the average mRNA levels in breast cancer types. (**F**) Breast cancer specimens TMA stained with an anti-CD73 antibody and representative pictures of different breast cancer subtypes are shown. Scale bars: 100 μm.(**G**) Quantified TMA consisting of luminal A, luminal B, Her2^+^, and TNBC samples immune stained for CD73. (**H**) The expression of CD73 in normal human mammary epithelial cells and various subtypes of breast cancer cells was detected by immunoblotting using an anti-CD73 antibody. (**I**) Kaplan-Meier curves using multivariable Cox proportional hazard model for the corresponding CD8^+^ T cells and CD73 expression. A total n = 1,100 breast cancer patients were included in the study, with 20 cases having missing data. The analysis was performed on the remaining 1,080 patients, of whom 153 had died, and Kaplan-Meier curves were subsequently plotted. Low- and high-expression groups refer to patients with expression levels lower and greater than the 50^th^ percentile, respectively. **P < 0.01, ***P < 0.001, and ****P < 0.0001, statistical significance was determined by 1-way ANOVA with Tukey’s multiple comparisons test for immunostaining using *n* = 110 breast samples.

To search for potential therapeutic targets for immune-cold breast cancer, we performed a bioinformatic analysis of proteomic data concerning over 300 immune-relevant proteins from 59 breast cancer patients in The Cancer Genome Atlas (TCGA) (4, 21). Elevated expression of immune-relevant proteins, including VTCN1, CD274 and CD73, were detected in the basal TNBC subtype of breast cancer compared with other subtypes (Figure 1A and Supplemental Table 2; supplemental material available online with this article; https://doi.org/10.1172/JCI176390DS1). Subsequent hierarchical cluster analysis revealed 2 distinct subgroups of patients with TNBC, each characterized by a unique immune-related protein expression profile (Figure 1B). Further differential protein expression analysis spotlighted the significant upregulation of CD73 in one of these subgroups (Figure 1C). Given the status of PD-L1 as a recognized biomarker for the “immune hot” classification of patients with TNBC, we examined the expression levels of both PD-L1 and CD73 across the 2 TNBC cohorts. Interestingly, while the average PD-L1 expression remained consistent across both cohorts except for the change in the width of the distribution, CD73 was markedly upregulated in one subgroup (TNBC group 1, Figure 1D), suggesting the presence of a TNBC subgroup with a potential immune-suppressive profile.

Additionally, Figure 1E showed that the mRNA expression of CD73 and several mRNAs involved in the adenosinergic pathway were significantly upregulated in immune-noninflamed TNBC samples but downregulated in immune-inflamed and restrained samples from patients with TNBC (GEO-accession number: GSE88847) (22). In contrast, the mRNAs downregulated in immune-noninflamed TNBCs but upregulated in immune-inflamed TNBCs were mostly associated with IFN-γ signaling pathway, which facilitated tumor cell recognition and elimination by recruiting cytotoxic T lymphocytes to tumor cells (Figure 1E and Supplemental Figure 1A).

To confirm the observations in Figure 1, A–E, a tissue microarray (TMA) comprising 110 distinct breast samples was examined using IHC. We identified what we considered to be a remarkable elevation in CD73 protein levels detected in the majority of TNBC tissue specimens (Figure 1, F–G). We next performed a systematic analysis of both protein expression and mRNA levels of CD73 across a wide spectrum of breast cancer cell lines, encompassing a total of 45 different lines (23). A similar expression pattern was measured in both CD73 protein and mRNA levels, where CD73 was significantly elevated in TNBC cells in comparison with other breast cancer cell lines (Figure 1H and Supplemental Figure 1, B–D).

To investigate the potential association between heightened CD73 expression and reduced tumor immunogenicity, we conducted a Gene Ontology Biological Process (GOBP) analysis using the TCGA breast cancer database. Results presented in Supplemental Figure 2A indicated a strong correlation between increased CD73 expression and biological processes related to tumor immune response, such as adaptive immune response (24). Additionally, the CIBERSORT analysis (25) in Supplemental Figure 2B verified the correlation between elevated CD73 expression in breast tumors and a reduced CD8^+^ T cell infiltration. To validate the observations in Supplemental Figure 2B and examine the association between CD73 expression and CD8^+^ T cell infiltration, the results of a breast cancer TMA presented in Supplemental Figure 2, C–D, demonstrated a marked reduction in CD8^+^ T cell staining in TNBC specimens with elevated CD73 expression. Moreover, spearman correlation analysis indicated a subset of TNBC tumors with diminished CD8^+^ T cell infiltration, particularly in cases with high CD73 expression. Likewise, a rise in CD73 expression was associated with the downregulation of immune response, T cell activation, and proliferation, as illustrated in Supplemental Figure 2, E–G, and Supplemental Figure 3, A and B, using the GOBP enrichment score and immune suppressive gene correlation analysis. To further determine the prognostic significance of CD73 expression in patients with TNBC, we examined 1,080 TNBC samples from TCGA database (25). There was a positive correlation between high CD73 expression and increased hazard risk and adverse outcomes in overall survival (OS) (Supplemental Figure 3, C and D), as indicated by univariate Cox regression models. Ultimately, we assessed the cumulative survival by considering both CD73 expression and CD8^+^ T cell population. As depicted in Figure 1I, a marked improvement in patient survival was observed among those with a high CD8^+^ T cell population but low CD73 expression. These findings demonstrated a significant correlation between aberrant increase in CD73 and poor prognosis of immune-suppressive TNBCs.

### Identification of OTUD4, a deubiquitinase, as a key driver for CD73 accumulation in immune-suppressive TNBCs.

**Figure 2:**
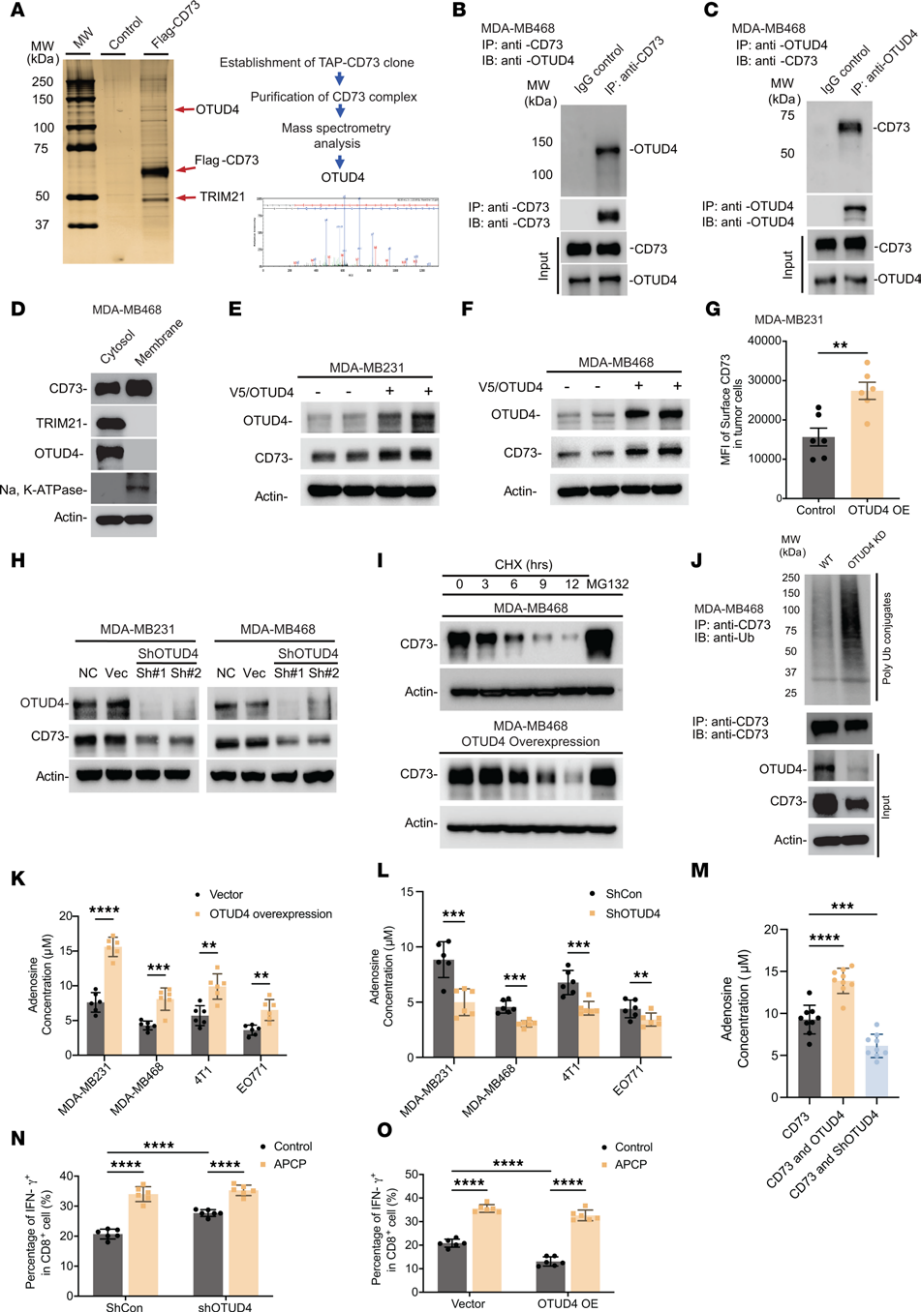
Identification of OTUD4 as a key driver for CD73 accumulation in immune-suppressive TNBC breast tumors. (**A**) CD73 complex was purified with a tandem-affinity purification protocol followed by mass spectrometry analysis in MDA-MB468-Flag/HA-CD73 cells. Silver staining of the purified CD73 complex was illustrated. OTUD4 was identified as a binding partner of CD73, and the representative spectra were included. (**B** and **C**) Validation of biochemical interaction between CD73 and OTUD4 in MDA-MB468 cells by coimmunoprecipitation of endogenous CD73 (**B**) and by coimmunoprecipitation of endogenous OTUD4 (**C**). (**D**) Cellular fractionated protein (cytosol versus membrane) expression of CD73 and OTUD4 in MDA-MB468 cells was determined. (**E** and **F**) CD73 protein levels were determined in MDA-MB231-OTUD4 (**E**) and MDA-MB468-OTUD4 (**F**) breast cancer cells. (**G**) The MFI of membrane-expressed CD73 was determined by flow cytometry in both MDA-MB231 and MDA-MB231-OTUD4 cells. (**H**) CD73 protein levels were determined in MDA-MB231-ShOTUD4 and MDA-MB468-ShOTUD4 cells. (**I**) MDA-MB468 and MDA-MB468-OTUD4 cells were treated with cycloheximide (CHX) or MG132, and CD73 protein levels were determined. (**J**) Validation of CD73 ubiquitylation by Coimmunoprecipitation of endogenous CD73 in both MDA-MB231 and MDA-MB231-ShOTUD4 cells. (**K** and **L**) The adenosine levels were determined in MDA-MB231, MDA-MB468, 4T1, and EO771 breast cancer cells with OTUD4 overexpression (**K**) or ShOTUD4 (**L**). (**M**) The adenosine levels were determined in MDA-MB468-CD73^WT^, MDA-MB468-CD73^WT^-OTUD4, and MDA-MB468-CD73^WT^-ShOTUD4 cells. (**N** and **O**) MDA-MB468, MDA-MB468-ShOTUD4 (**N**), and MDA-MB468-OTUD4-OE (**O**) were cocultured with human PBMCs with or without APCP treatment, and CD8^+^ IFN-γ^+^ T cell population was measured and quantified using flow cytometry (ShCon, control shRNA). Data (mean ± SEM) are representative of at least 3 independent experiments. **P < 0.01, ***P < 0.001 and ****P < 0.0001, by 1-way ANOVA with Tukey’s multiple comparisons test.

To explore the underlying mechanism that governs the abnormal accumulation of CD73 in immune-suppressive TNBC breast tumors, we have conducted a Tandem Affinity Purification (TAP) coupled with mass spectrometry analyses to identify CD73 interactome (4). Flag/HA-tagged hCD73 protein was ectopically expressed in MDA-MB-468 cells and subsequently purified using an affinity capture approach, with cells transfected with the 3 × Flag vector serving as the control (4). This approach led to the identification of OTUD4 as a putative critical driver for upregulating CD73 in TNBC cells (Figure 2A). In addition, TRIM21 was identified as the ubiquitin E3 ligase that suppresses CD73 expression levels through ubiquitin-proteasomal degradation (9). To validate the biochemical consequences as well as physiological relevance of interaction between OTUD4 and CD73, we performed a series of immunoprecipitation assays in the MDA-MB468 cells (26). As shown in Figure 2, B and C, we observed endogenous CD73 coimmunoprecipitated with OTUD4, while CD73 was detected on OTUD4 immunocomplexes. To further validate the generality of the OTUD4/CD73 interaction, a series of immunoprecipitation assays across diverse cancer cell lines, including the nonsmall cell lung cancer cell HCC827, human ovarian cancer cell SKOV3, and human colorectal cancer cell HCT116, consistently showed endogenous interaction between CD73 and OTUD4 (Supplemental Figure 4, A–D). To examine the cellular compartmentalization of the interaction between CD73 and OTUD4, we conducted cell lysate fractionation followed by Western blotting (27). As shown in Figure 2D, we observed that both OTUD4 and TRIM21 principally existed in the cytosol. We further identified the intracellular interaction site between OTUD4 and CD73 using an in vitro immunofluorescence staining and proximity ligation assay (4). As presented in Supplemental Figure 4E, although CD73 (green) was observed both in membrane and cytosol and OTUD4 (red) was observed both in nucleus and cytosol, the interaction between CD73 and OTUD4, in the cytosol as determined by proximity ligation assay (Supplemental Figure 4F), suggested a potential link between OTUD4-guided cytosolic deubiquitylation of CD73 and its subsequent displacement onto the cell membrane. To assess whether OTUD4 could directly affect CD73 turnover via its deubiquitinase activity, we engineered MDA-MB468 and MDA-MB231 human breast cancer cell lines with stable OTUD4 expression or knockdown (KD). As shown in Figure 2, E–G, elevated OTUD4 resulted in increased CD73 protein levels (Figure 2, E and F). To further assess whether the deubiquitylation of CD73 protein guided by OTUD4, leading to its stabilization, could result in enhanced membrane presentation, the results in Figure 2G and Supplemental Figure 4G indicated that the fluorescence intensity of membrane-bound CD73 in OTUD4-overexpressing (OE) MDA-MB231 (Figure 2G) and MDA-MB468 (Supplemental Figure 4G) cells was significantly higher than that in the control cells. In addition, immunofluorescence staining in MDA-MB468-OTUD4 further confirmed that enhanced OTUD4 expression leads to increased membrane-bound CD73 fluorescence intensity (Supplemental Figure 4H). In contrast, the KD of OTUD4 using shRNA in MDA-MB231 and MDA-MB468 cells led to a downregulation of both total and membrane-bound CD73 protein expression (Figure 2H and Supplemental Figure 4I). To determine if OTUD4 exerted a direct effect on CD73 turnover dynamics, we performed a pulse-chase assay in MDA-MB468 cells stably OE OTUD4 and found that overexpression of OTUD4 significantly slowed down CD73 protein turnover (Figure 2I). To examine the role of OTUD4 on CD73 degradation through catalyzing CD73 deubiquitylation, we performed a CD73 ubiquitylation assay using MDA-MB468 cells with OTUD4 stable KD. Figure 2J reveals that endogenous CD73 underwent ubiquitylation, and the knockdown of OTUD4 markedly increased the ubiquitin-conjugated CD73.

To ascertain whether the physiological relevance for the accumulation of CD73 is regulated by OTUD4, we evaluated adenosine production in both human and mouse TNBC breast cancer cells (MDA-MB231, MDA-MB468, 4T1, and EO771). Figure 2, K and L showed that OTUD4 overexpression in breast cancer cells significantly augmented adenosine production compared with the control, whereas OTUD4 KD resulted in a reduction of adenosine production. Similar results were also observed even in the case of CD73-overexpressed MDA-MB486 and MDA-MB231 cells (Figure 2M and Supplemental Figure 5A). In addition, we explored if modulation of OTUD4 could influence other enzymes within the adenosinergic pathway, including CD39 and adenosine deaminase (ADA). Results in Supplemental Figure 5, B–E clearly demonstrated that neither AMP production nor ADA activity was significantly altered by the levels of OTUD4 expression in MDA-MB468 cells. Further, we investigated the effect of CD73 deubiquitylation by OTUD4 in cancer cells on T cell function in vitro using a coculture experiment (Figure 2, N and O, and Supplemental Figure 5, F–I). T cell proliferation activity and IFN-γ production were elevated when OTUD4 was knocked down in tumor cells, whereas OTUD4 overexpression in tumor cells led to a decrease in T cell proliferation activity and IFN-γ production. The above results demonstrate that deubiquitinase OTUD4 is a key driver for sustaining CD73 protein accumulation with concomitant extracellular adenosine production, thereby limiting T cell function in immune-suppressive TNBCs.

### Mapping and structural modeling of the interaction between CD73 and OTUD4 by integrated use of experimental data and docking simulations followed by experimental validation.

**Figure 3:**
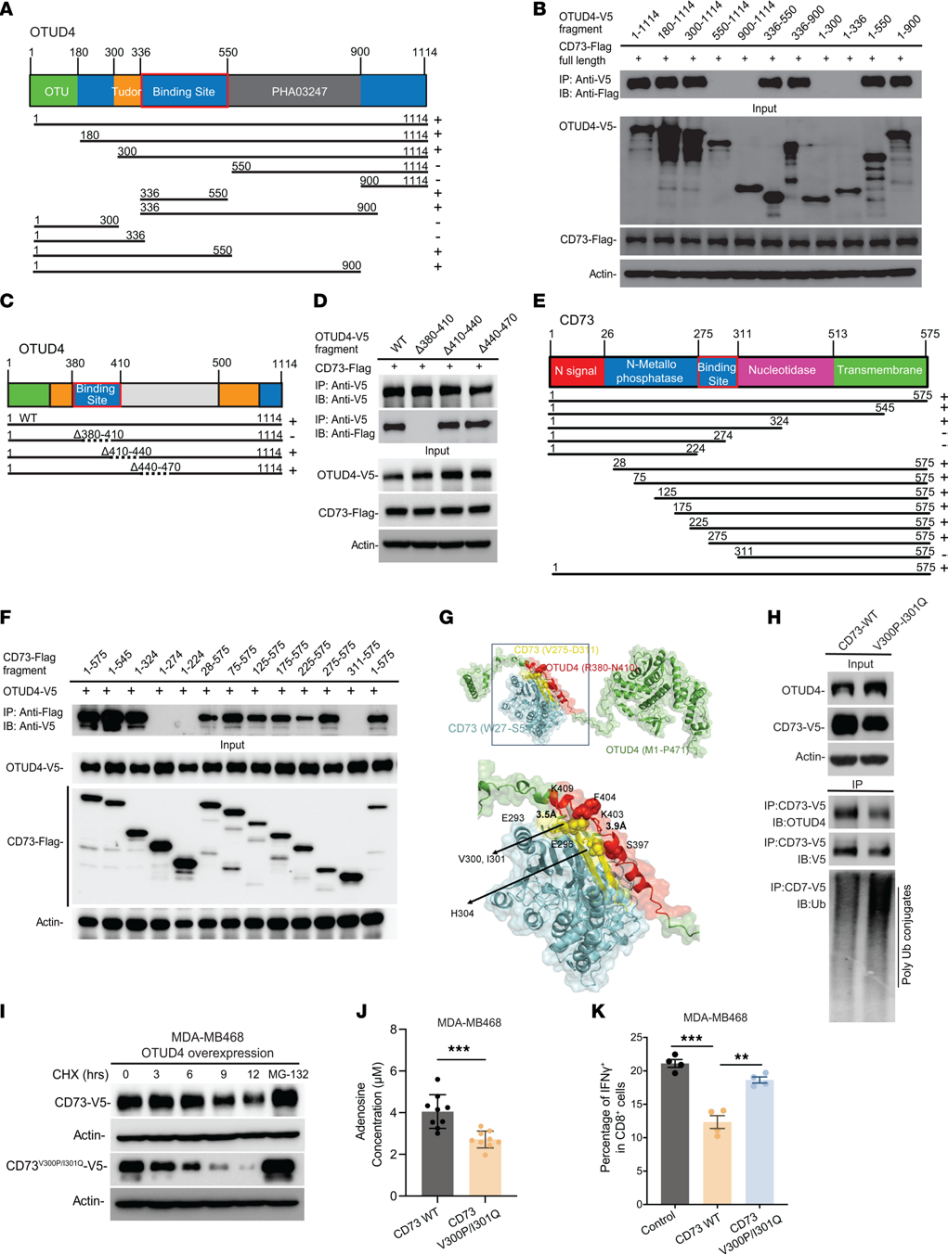
Mapping of molecular regions that facilitate the interaction between CD73 and OTUD4 and 3D structural modeling of CD73 regulation based on the interplay of ubiquitylation and deubiquitylation. (**A**–**D**) Schematic diagram of human OTUD4 domains and strategy to engineer a series of OTUD4 deletion mutants. Mapping of the molecular domain on OTUD4 involving in the interaction with CD73. The interactions between CD73 and OTUD4 fragments were examined by coimmunoprecipitation experiments in HEK-293T cells. Amino acids stretching from 380–410 on OTUD4 (**D**) were identified as the region that mediates the interaction between OTUD4 and CD73. (**E**) Schematic diagram of human CD73 domains and strategy to engineer a series of CD73 deletion fragments. (**F**) The interactions between OTUD4 and CD73 fragments were determined. Amino acids stretching from 275–311 on CD73 were identified as the region that mediates the interaction between OTUD4 and CD73. (**G**) Structural model for the interaction of CD73 with OTUD4. Residues R380–N410 (red) of OTUD4 (green) interact with the residues V275–D311 (yellow) of CD73 (cyan). Salt bridges are formed between E296 and E293 of CD73 and K409 and K403 of OTUD4, respectively. The interface is further stabilized by hydrophobic interactions between V300 and V301 of CD73 (yellow) and F404 (red) of OTUD4, and contacts between H304 (yellow) of CD73 and S397 (red) of OTUD4. (**H**) Validation of interaction domains between OTUD4 with CD73 by coimmunoprecipitation of ectopic V5-tagged CD73^WT^ and V5-tagged CD73^V300P/I301Q^ mutant in HEK-293T cells. The ubiquitylation status of CD73^WT^ and CD73^V300P/I301Q^ mutant was also determined. (**I**) MDA-MB468-CD73^WT^ and MDA-MB468-CD73^V300P/I301Q^ cells were treated with CHX and MG-132 and CD73 protein turnover were determined. (**J**) The adenosine levels were determined in MDA-MB468-CD73^WT^ and MDA-MB468-CD73^V300P/I301Q^. (**K**) MDA-MB468, MDA-MB468-CD73^WT^ and MDA-MB468-CD73^V300P/I301Q^ were cocultured with human PBMCs and percentage of IFN-γ ^+^ in CD8^+^ T cell population quantified using flow cytometry. Data (mean ± SEM) are representative of at least 3 independent experiments. **P < 0.01 and ***P < 0.001, by unpaired *t* test, 1-way ANOVA with Tukey’s multiple comparisons test.

To elucidate the in-depth mechanism by which CD73 is regulated by OTUD4, we have identified the interaction domains that facilitate the recognition and binding between OTUD4 and CD73. To this end, we constructed Flag/HA-tagged CD73 fragments and V5-tagged OTUD4 fragment mutants (Figure 3, A–F) and cotransfected them into HEK293T cells, followed by coimmunoprecipitation experiments. We then cotransfected a series of V5-tagged OTUD4 truncation mutants (Figure 3A) with Flag/HA-tagged CD73, respectively, and conducted a pull-down assay of individual V5-tagged OTUD4 mutant, followed by immunoblotting with anti-Flag antibody to detect Flag/HA-CD73. As illustrated in Figure 3B, amino acids stretching from 336 to 550 on OTUD4 mediated the interaction with CD73. We further narrowed down to amino acids stretching from 380 to 500 on OTUD4 that mediated the interaction between OTUD4 and CD73 (Supplemental Figure 6, A and B). Moreover, we engineered a series of OTUD4 mutants, specifically deleting the amino acid segments 380–410, 410–440, and 440–470. We demonstrate that the OTUD4 mutant without the R380–N410 segment lost its ability to bind with CD73, as evidenced by coimmunoprecipitation (Figure 3, C and D). A similar strategy was utilized to identify the molecular motifs on CD73 that are responsible for the interaction with OTUD4. As shown in Figure 3, E and F, the amino acid residues from 275 to 311 on CD73 were pinpointed as the region mediating the binding between CD73 and OTUD4.

We have recently explored and characterized the role of TRIM21 acting as an E3 ligase responsible for the ubiquitin-proteasomal degradation of CD73 (9). To comprehensively address the mechanism by which the interplay of TRIM21-mediated ubiquitylation and OTUD4-guided deubiquitylation regulates CD73 proteolysis, we conducted structural modeling of the interaction between CD73, TRIM21, and OTUD4 by integrated use of experimental data and docking simulations followed by experimental validation. The model in Supplemental Figure 3C features 19 hydrogen bonds (computed using HBplus) (28) and 2 salt bridges between CD73 amino acids E203 and D205 and TRIM21 residues H116 and K104. In addition, the PRY/SPRY domain of TRIM21 interacts with CD73 similar to its interaction with IGG Fc (PDB: 2IWG) (29), as demonstrated in Supplemental Figure 3D, underscoring the propensity of TRIM21 PRY/SPRY domain to engage in molecular associations.

As mentioned above, experiments revealed that OTUD4 residues R380–N410 engaged with CD73 residues V275–D311. Figure 3G displays the model where the interface (of 826.7 Å^2^) exhibited the highest overlap with the experimentally detected regions for the 2 proteins, mainly burying 90.5% and 65.5% of interfacial residues identified experimentally for the respective proteins. The interface is further characterized by 14 hydrogen bonds and 2 salt bridges.

To identify the key amino acids that drive the OTUD4/CD73 interaction, Supplemental Figure 6E details the types and strengths of inter-residue interactions. Predominant interfacial residue pairs include H304(CD73)–S397(OTUD4), the salt bridge E293(CD73)–K403(OTUD4), and the hydrophobic contacts of CD73 V300 and I301 with OTUD4 F404 (lower panel of Figure 3G). Given the significance of hydrophobic contacts at the CD73-OTUD4 interface, we focused on CD73 V300 and I301. The heatmap in Supplemental Figure 6F lists the values for all substitutions of amino acids at positions 300 (ordinate) and 301 (abscissa). Specifically, we selected the V300P and I301Q double mutation that elevates the by 1 kcal/mol, while reducing CD73’s by 5.2 kcal/mol. Comprehensive mutation effects are illustrated in Supplemental Figure 6G. To further confirm if amino acids V300 and I301 on CD73 directly mediate its binding to OTUD4, the results depicted in Figure 3H and Supplemental Figure 6H reveal a marked decrease in the interaction between mutant CD73 and OTUD4 when the amino acids V300 and I301 are mutated in both MDA-MB231 and MDA-MB468 cells. Additionally, we observed a rise in the level of ubiquitin conjugates in the CD73^V300P/I301Q^ double mutant, thereby confirming that V300/I301 on CD73 mediates the interaction and subsequently influences CD73 stability through regulation of deubiquitylation. In addition, in MDA-MB468 cells, while the turnover of ectopically expressed CD73 displayed a half-life of approximately 4 hours, the CD73^V300P/I301Q^ interaction mutant notably accelerated CD73 turnover (Figure 3I).

To further demonstrate that the CD73^V300P/I301Q^ mutant also influences CD8^+^ T cell proliferation via CD73-dependent enzymatic adenosine production, Figure 3J and Supplemental Figure 6, I–K showed that cells harboring CD73^V300P/I301Q^ mutation exhibited substantially lower adenosine levels compared with CD73^WT^ cells. As indicated in Figure 3K and Supplemental Figure 6L, MDA-MB468-CD73^WT^ cells had a decrease in IFN-γ production and proliferation capacity of CD8^+^ T cell population than MDA-MB468 vehicle control cells, and the disruption of the CD73-OTUD4 interaction by mutating the V300/I301 sites restored the IFN-γ production and proliferation capacity of CD8^+^ T cell population comparable to those of the control vector. The data demonstrate the specific interaction between CD73 and OTUD4, dictated through the V300 and I301 residues on CD73, plays a pivotal role in governing CD73 protein stability and subsequent adenosinergic effect, particularly on modulation of T cell activity.

### Stabilization of CD73 by OTUD4 in immune-cold tumors is orchestrated in response to TGF-β signaling.

**Figure 4:**
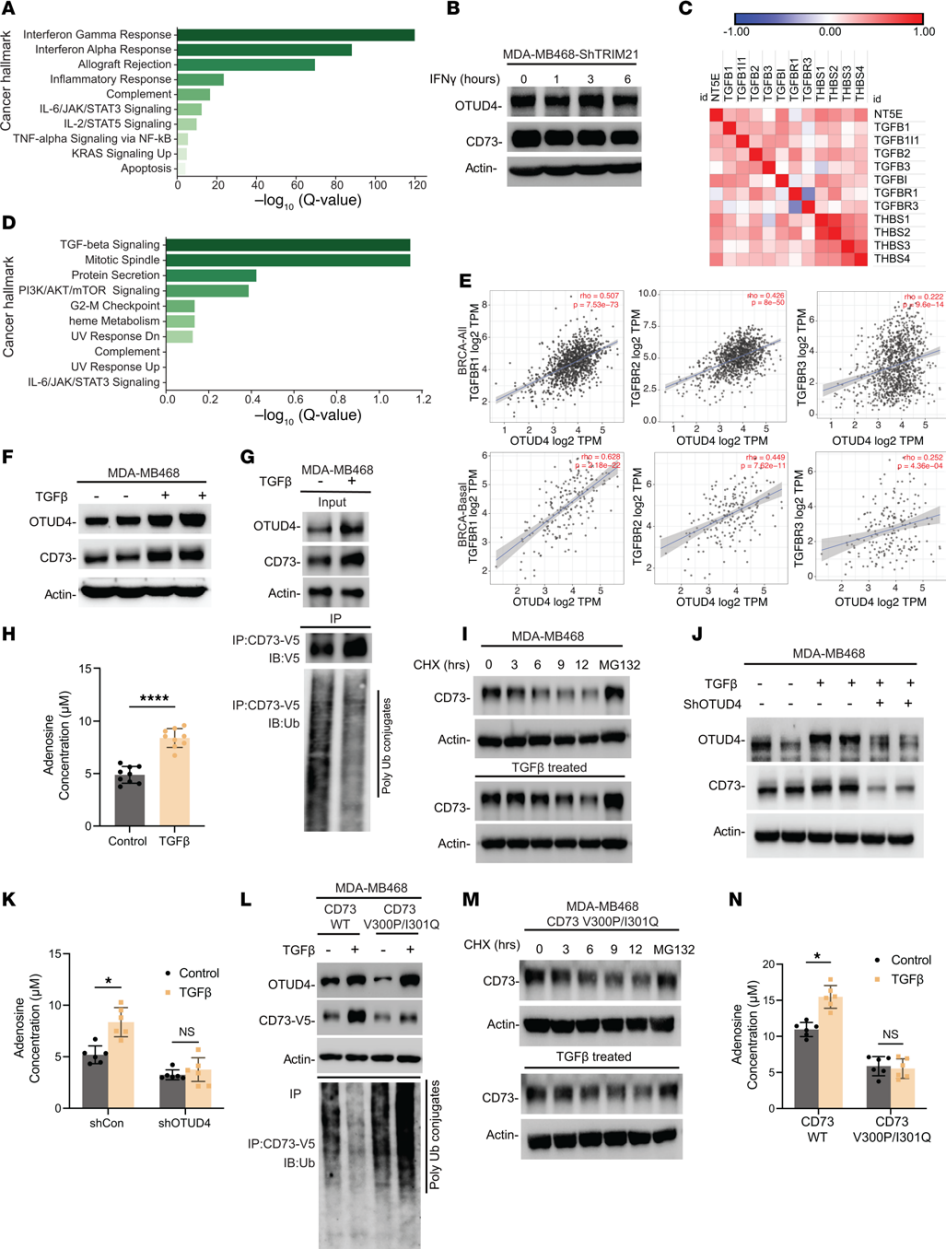
Stabilization of CD73 by OTUD4 in immune-cold tumors is orchestrated in response to TGF-β signaling. (**A**) MSigDB-based pathway enrichment analysis shows that CD73 expression in TCGA TNBC samples is positively correlated with TGF-β signaling hallmark. (**B**) MDA-MB468-ShTRIM21 cells were treated with IFN-γ (100 μg/mL), and OTUD4 and CD73 protein levels were determined. (**C**) Spearman’s rank correlation analysis using CPTAC data set showing the CD73 protein expression is highly positively correlated with TGF-β signaling pathway–related proteins. (**D**) The MSigDB-based pathway enrichment analysis showing that OTUD4 expression in TCGA TNBC samples is positively correlated with TGF-β signaling hallmark. (**E**) Spearman’s correlation analysis showing the positive correlation between OTUD4 expression and TGF-β signaling pathway. (**F**) MDA-MB468 were treated with 3 ng/mL TGF-β, OTUD4, and CD73 protein levels were determined by immunoblotting. (**G**) MDA-MB468-CD73-V5 were treated with 3 ng/mL TGF-β, CD73 immune complexes were immunoprecipitated and ubiquitylation levels were determined. (**H**) MDA-MB468 cells were treated with 3 ng/mL TGF-β, adenosine production levels were determined. (**I**) MDA-MB468 cells were treated with CHX and MG-132. Cell lysates were collected at indicated time points and followed by measuring CD73 protein expression. (**J** and **K**) MDA-MB468-ShCon and MDA-MB468-ShOTUD4 were treated with 3 ng/mL TGF-β, OTUD4 and CD73 protein levels (**J**) and adenosine production (**K**) were determined. (**L**) MDA-MB468-CD73^WT^ and MDA-MB468-CD73^V300P/I301Q^ cells were treated with 3 ng/mL TGF-β, V5-tagged CD73 and CD73^V300P/I301Q^ were immunoprecipitated and ubiquitylation level was determined. (**M** and **N**) CD73 protein turnover rate (**M**) and adenosine production level (**N**) were determined in MDA-MB468-CD73^V300P/I301Q^ and TGF-β–treated MDA-MB468-CD73^V300P/I301Q^ cells. Data (mean ± SEM) are representative of at least 3 independent experiments. *P < 0.05 and ****P < 0.0001, by unpaired *t* test, 1-way ANOVA with Tukey’s multiple comparisons test.

We have shown the TRIM21-mediated ubiquitylation and subsequent degradation of CD73 by a feed-forward mechanism involving IFN-γ (9). Our analysis using TCGA database corroborated this by indicating a positive correlation between high TRIM21 expression and increased gene signatures related to the IFN-γ pathway in patients with TNBC (Figure 4A and Supplemental Figure 7A). This observation raises the question of whether IFN-γ affects the deubiquitylation of CD73 orchestrated by OTUD4. Interestingly, there was no change in OTUD4 protein levels in MDA-MB468-ShTRIM21 cells with IFN-γ treatment, suggesting little contribution of IFN-γ to affect OTUD4 activity in tumor cells (Figure 4B). It is noted that the IFN-γ signaling pathway is often counteracted by the TGF-β pathway in immunosuppressive tumor microenvironments (30). Consistent with the importance of TGF-β signaling pathway for sustaining CD73 expression, TNBC cells with high CD73 expression exhibited increased protein expression related to the TGF-β signaling pathway (Figure 4C). Further, our GSEA analysis and TIMER2.0 analysis in Figure 4, D and E indicated a significant correlation between high OTUD4 expression and increased TGF-β signaling pathway activity. These findings led us to speculate that, in a subset of immunosuppressed breast tumors, upregulated TGF-β signaling may coopt OTUD4 to hyperactively deubiquitylate CD73, thereby enhancing its stability and membrane abundance, immune evasion, and maintaining a protumor microenvironment.

To test this hypothesis, we treated MDA-MB468 (Figure 4F) and MDA-MB231 (Supplemental Figure 8A) breast cancer cells with 3 ng/ml of TGF-β. Distinct from IFN-γ, TGF-β treatment increased the protein levels of OTUD4 together with CD73. Next, we observed a significant reduction in the ubiquitylation of CD73 following TGF-β treatment, as shown in Figure 4G. Additionally, an increase of cytosolic OTUD4 was noted following TGF-β exposure in both TGF-β–treated MDA-MB468 and MDA-MB231 cells, as shown in Supplemental Figure 8B. Moreover, we measured an increase in extracellular adenosine production in both MDA-MB468 and MDA-MB231 cells treated with TGF-β in Figure 4H and Supplemental Figure 8C.

Additionally, a pulse-chase assay in TGF-β treated MDA-MB468 cells and found that TGF-β treatment significantly slowed down CD73 protein turnover (Figure 4I). To further elucidate the influence of TGF-β on the OTUD4/CD73 axis, we treated MDA-MB468 and MDA-MB231 cells with stable KD of OTUD4 with 3 ng/mL TGF-β. As shown in Figure 4J and Supplemental Figure 8D, depletion of OTUD4 resulted in unchanged CD73 levels after TGF-β treatment, indicating OTUD4’s crucial role in maintaining CD73 stability. We also found that the TGF-β–induced increase in adenosine production (Figure 4K and Supplemental Figure 8E) was abolished completely when OTUD4 was knocked down. Additionally, knocking down of TGFBR or loss of TGF-β by LY2109761 led to reduced protein levels of OTUD4 as well as CD73. However, we still observed the interaction between OTUD4 and CD73, although its proportion correlated with the decreased level of OTUD4 and CD73 proteins (Supplemental Figure 8F). Collectively, these results imply that TGF-β may enhance OTUD4 levels, contributing to the stabilization of CD73.

To confirm the role of amino acids V300 and I301 in facilitating the TGF-β–mediated deubiquitylation of CD73 by OTUD4, Figure 4L shows that TGF-β treatment reduced the ubiquitylation level, which in turn stabilized the CD73 protein in MDA-MB-468-CD73^WT^ cells. In contrast, the TGF-β–induced upregulation of CD73 in MDA-MB468 cells was abrogated completely when the interaction sites between CD73 and OTUD4 were disrupted. Additionally, the degradation rate of CD73 protein remained unchanged in MDA-MB-468-CD73^V300P/I301Q^ cells, regardless of TGF-β treatment, as demonstrated by a pulse-chase assay (Figure 4M). In line with these results, an increase in adenosine production was noted only in the CD73^WT^ cells but not in the CD73^V300P/I301Q^ mutant cells (Figure 4N and Supplemental Figure 8G). These findings indicate the importance of a TGF-β–mediated OTUD4/CD73 proteolytic axis for the modulation of tumor immunogenicity.

### Development of a pharmacological inhibitor that blocks OTUD4/CD73 interaction in restoring tumor immune responses in immune-suppressive TNBCs.

**Figure 5:**
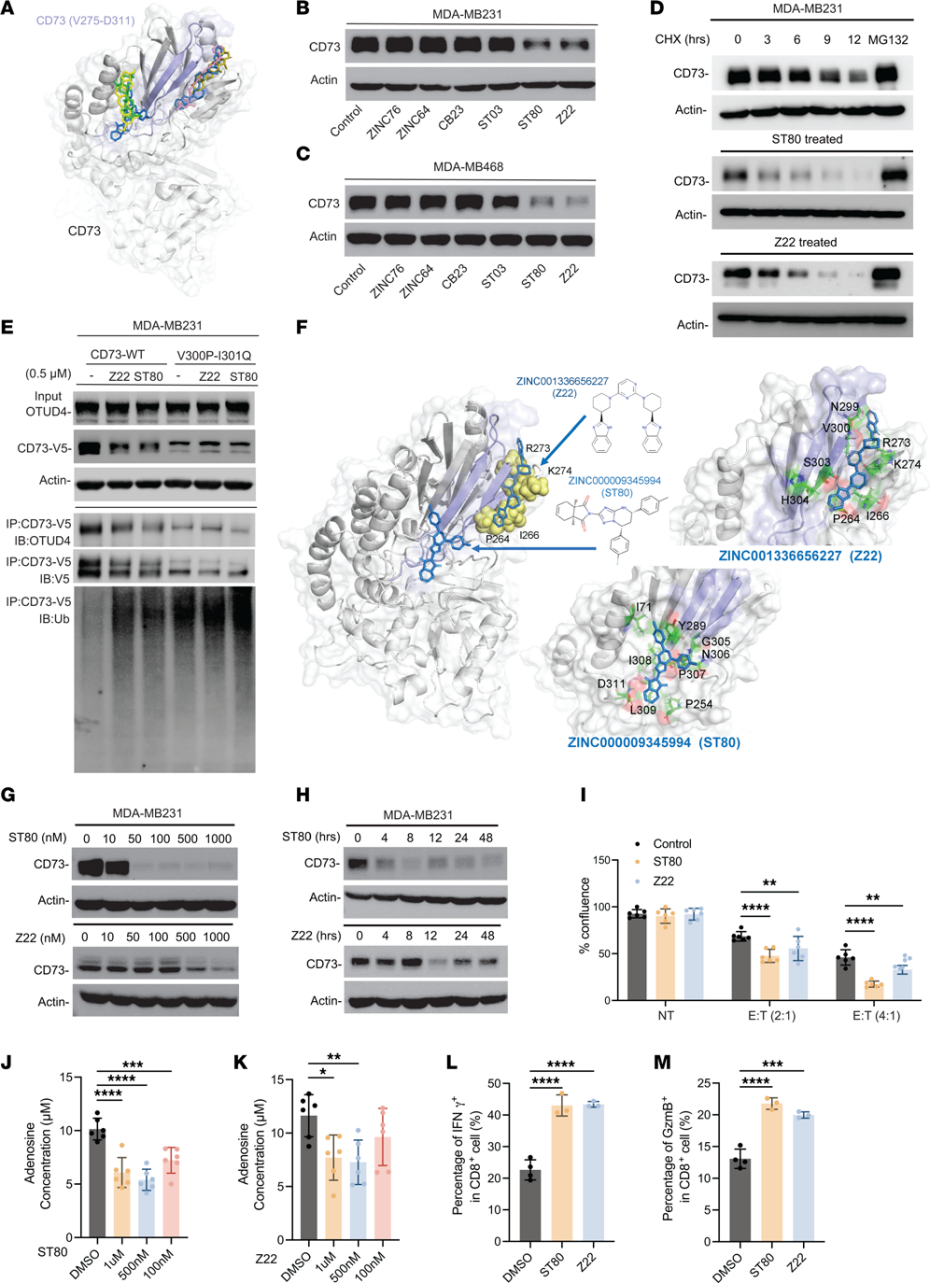
Development of pharmacological inhibitor that blocks OTUD4/CD73 interaction in restoring tumor immune response in immune-suppressive breast cancer. (**A**) Binding sites of the 6 small molecules on CD73. The diagram displays the superposition of all 6 small molecules illustrating that they essentially target 2 distinct sites in the vicinity of the region V275–D311 of CD73 (in indigo) which makes interfacial contacts OTUD4 in the CD73/OTUD4 complex. (**B** and **C**) MDA-MB231(**B**) and MDA-MB468 (**C**) were treated with 0.5 μM screened compounds and CD73 expression levels were determined. (**D**) MDA-MB231 were treated with 0.5 μM ST80 or Z22 and CD73 protein turnover rate was determined by pulse-chase analysis. (**E**) MDA-MB231-CD73^WT^ and MDA-MB231-CD73^V300P/I301Q^ cells were treated with 0.5 μM ST80 and Z22, CD73 immune complexes were immunoprecipitated, OTUD4, CD73, and CD73 ubiquitylation were determined by immunoblotting. (**F**) Coordination of ST80 and Z22. ST80 (marine) and Z22 (blue) are located in and around residues V275–D311 (indigo) of CD73. The yellow spheres correspond to the labelled CD73 residues that predominantly interact with Z22. (**G**) MDA-MB231 cells were treated with different doses of ST80 or Z22 for 24 hours and CD73 protein levels were determined. (**H**) MDA-MB231 cells were treated with 0.5 μM ST80 or Z22, cell lysates were collected at different time points,and CD73 protein levels were determined. (**I**) MDA-MB231 cells were cocultured with human PBMCs at different Effector (E) to Target (T) ratios (2:1 or 4:1) and treated with ST80 and Z22 (0.5 μM), and cell proliferation rate was determined. (**J** and **K**) MDA-MB231 cells were treated with 1 μM, 0.5 μM, and 0.1 μM ST80 (**J**) and Z22 (**K**), adenosine productions were determined. (**L** and **M**) MDA-MB231 cells were cocultured with human PBMCs and treated with 0.5 μM ST80 or Z22. Percentage of IFN-γ^+^ in CD8^+^ T cells (**L**) and percentage of GzmB^+^ in CD8^+^ T cells (**M**) were measured and quantified using flow cytometry. Data (mean ± SEM) are representative of at least 3 independent experiments. *P < 0.05, **P < 0.01, ***P < 0.001, and ****P < 0.0001, by 1-way ANOVA with Tukey’s multiple comparisons test.

We next aimed to determine the therapeutic consequence of blocking the OTUD4/CD73 complex formation toward restoring tumor immune response in immune-suppressive TNBCs. We implemented a triphase methodology to identify small molecules that could block the interaction between CD73 and OTUD4 (Supplemental Figure 9A). Our approach primarily targeted the druggability of the CD73 residues V275-D311 that bind to OTUD4. This rigorous approach led to 9 promising small molecules (Supplemental Figure 9, B and C), 6 of which could be procured due to the unavailability of the other 3. The chemical structures of these 6 molecules are displayed in Supplemental Figure 9D, and their binding positions on CD73 are shown in Figure 5A. Notably, these small molecules target 2 distinct sites, both adjoining the interfacial segment V275-D311.

To verify whether the compounds identified through the above described in silico protocol could bind CD73 to block its interaction with OTUD4 and therefore affect CD73 protein levels, we initially treated MDA-MB231 and MDA-MB468 cells with 0.5 μM of the compounds for 24 hours and then assessed the CD73 protein levels. As shown in Figure 5, B and C, our systematic validation led to the identification of 2 compounds, ZINC000009345994 (ST80) and ZINC001336656227 (Z22), as the most potent inhibitors in suppressing CD73 protein abundance by blocking the OTUD4/CD73 interaction. Additionally, both ST80 and Z22 increased the turnover of the CD73 protein compared with the control in MDA-MB231 cells with 0.5 μM of both ST80 and Z22 (Figure 5D). To confirm whether the inhibitory effects of ST80 and Z22 were attributed specifically to targeting the interaction site of OTUD4 and CD73, we first treated mutant MDA-MB231-CD73^V300P/I301Q^ and MDA-MB468-CD73^V300P/I301Q^ cells with all 6 compounds. As seen in Supplemental Figure 10, A and B, there were no changes in CD73 protein levels. Figure 5E further illustrated that 0.5 μM ST80 and Z22 treatment decreased CD73 protein levels with increased ubiquitylation levels in MDA-MB231-CD73^WT^ cells but not in MDA-MB231-CD73^V300P/I301Q^ cells. These results together suggest that both ST80 and Z22 are potential pharmacological inhibitors that specifically block OTUD4/CD73 interaction to destabilize CD73 protein.

To understand the mechanisms by which ST80 and Z22 disrupt the interaction between OTUD4 and CD73, we illustrated in Figure 5F that ST80 binds to a hydrophobic area defined by residues I71, P254, Y289, P307, I308, and L309. In contrast, Z22 interacts directly with certain residues within the V275–D311 binding region, as well as with residues P264, I266, R273, and K274 of CD73. We further examined the influence of ST80 on the overarching dynamics of CD73: Supplemental Figure 10C displays the distribution of CD73 residue movements along a soft mode of motion, predicted by the Gaussian Network Model (31). This suggests that ST80 binding to this specific site strongly impacts the structural dynamics of CD73. As to the mode of action of Z22, the binding position shown in Figure 5F suggests that it acts as an allosteric inhibitor of the CD73-OTUD4 interaction. The heatmap presented in Supplemental Figure 10D displays the cross-correlation between the residues P264, I266, R273, and K274 that coordinate Z22, suggesting that Z22 binding to that specific pocket allosterically affects the conformation of the binding epitope, thus interfering with complex formation.

To further understand the inhibitory effects of ST80 and Z22 on the regulation of CD73 stability, we performed a series of experiments to determine the optimal dose and treatment duration for ST80 and Z22 to disrupt the OTUD4/CD73 interaction. MDA-MB231 and MDA-MB468 cells were treated with ST80 and Z22 at concentrations ranging from 10 nM to 1 μM for 24 hours. As illustrated in Figure 5G and Supplemental Figure 10E, ST80 significantly reduced CD73 protein levels starting from 50 nM while Z22 reduced CD73 protein levels starting from 500 nM in MDA-MB231 and MDA-MB468 breast cancer cells. We also treated MDA-MB231 and MDA-MB468 cells with various concentrations of ST80 and Z22 in conjunction with a 3 ng/mL administration of TGF-β. As illustrated in Supplemental Figure 10, F and G, ST80 was able to counteract the TGF-β–induced increase in CD73 protein levels starting at a concentration of 500 nM while Z22 required a 1 μM dose to yield comparable inhibitory effects. Further, the inhibitory effects of ST80 were evident as early as 4 hours after treatment and persisted for up to 48 hours in MDA-MB468 cells and MDA-MB231 cells (Figure 5H and Supplemental Figure 10H). In contrast, Z22 exerted its effects starting from 12 hours after treatment but only lasted 24 hours.

To determine the subsequent effects of ST80 and Z22 in cancer cell proliferation and tumor immune response, MDA-MB231 and MDA-MB468 cells were treated with 0.5 μM of ST80 and Z22 for 24 hours, followed by a clonogenic survival assay. As illustrated in Supplemental Figure 10I, there was no direct cytotoxicity of ST80 and Z22 against both cell lines. Conversely, when we cocultured MDA-MB231 and MDA-MB468 cells with human PBMC at varying effector-to-target (E:T) ratios, the cytotoxic effects of PBMC were more pronounced with the treatment of ST80 and Z22. Figure 5I and Supplemental Figure 10J highlighted that these compounds markedly enhanced the growth inhibition of cancer cells induced by PBMC, even at a low E:T ratio of 2:1. To further determine the impact of ST80 and Z22 on tumor immune responses, we demonstrated in Figure 5, J and K and Supplemental Figure 10, K and L that ST80 reduced adenosine production starting at concentrations as low as 100 nM in MDA-MB231 and MDA-MB468 cells. In contrast, Z22’s effects become evident at either 100 nM or 500 nM concentrations, depending on the cell line. Further, upon coculturing MDA-MB231 cells with PBMCs, both compounds resulted in an increase in expression of IFN-γ (Figure 5L), GzmB (Figure 5M), Ki67 (Supplemental Figure 10M), and TNF-α (Supplemental Figure 10N) in t he CD8^+^ T cell population. To determine if ST80 could exert a broad therapeutic effect in a spectrum of cancers, we found that ST80 treatment led to a pronounced reduction in CD73 protein levels at a concentration of 500 nM in HCC827 lung cancer cells (Supplemental Figure 11A). Moreover, ST80 treatment appeared to enhance the cytotoxic function of T cells, leading to increased cytotoxic effects of PBMC in multiple cancer types, including HCC827 lung cancer cells, SKOV3 ovarian cancer cells, and HCT116 colon cancer cells (Supplemental Figure 11, B–K). These findings support our hypothesis that ST80 and Z22 specifically disrupt the OTUD4/CD73 protein interaction, thereby reducing CD73 protein stability and ultimately boosting tumor immune responses.

### Modulation of CD73 proteolysis by OTUD4 affects tumor growth and capacity to elicit antitumor CD8^+^ T cell responses.

**Figure 6:**
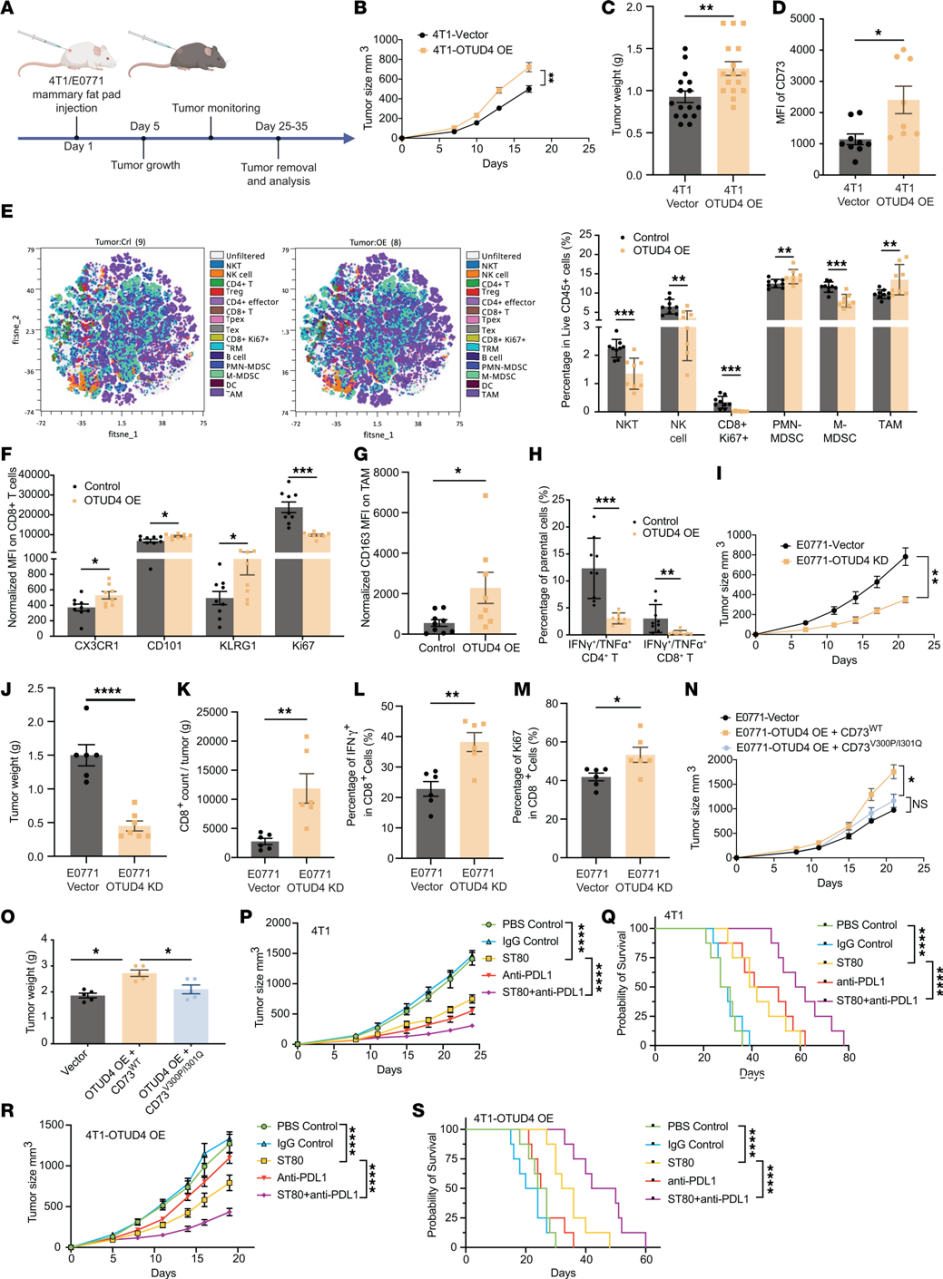
OTUD4-mediated deubiquitylation of CD73 orchestrates tumor immune evasion in vivo. (**A**) Schematic diagram of 4T1 control and EO771 breast cancer cells were orthotopically injected into the right fourth mammary gland of the BALB/c WT (WT) mice. PBS was used in the control mouse group. Tumor weight was measured at the endpoint, and the tumor growth curve was plotted. (**B**–**D**) 4T1 control and 4T1-OTUD4 overexpression tumors were harvested 21 days after tumor challenge, tumor growth curve was plotted (**B**), and tumor weight was measured at the endpoint (**C**). Membrane-bound CD73 was analyzed by flow cytometry (**D**). (**E**) Exemplified tSNE visualization of overlaid main immune cell population composition within tumor infiltrates. Summarized frequencies of total infiltrated main immune populations among live CD45^+^ cells were compared in 4T1 control tumor and 4T1-OTUD4 overexpression tumor at 18 days after tumor challenge. (**F**) The expression levels of CX3CR1, CD101, KLRG1, and Ki67 were measured in the tumor-infiltrating CD8^+^ T cells between 4T1-control tumors and 4T1-OTUD4–overexpression tumor. (**G**) The expression levels of CD163 were compared in tumor associated macrophages (TAMs) between 4T1 control tumors and 4T1-OTUD4 overexpression tumors. (**H**) Comparison of the percentage of IFN-γ^+^/TNF-α^+^ among tumor-infiltrated CD4^+^ or CD8^+^ T cells between 4T1 control tumor and 4T1-OTUD4 overexpression tumor. (**I** and **J**) EO771-control and EO771-OTUD4–KD tumors were harvested 21 days after tumor challenge and analyzed. Tumor growth curve was plotted (**I**) and tumor weight (**J**) was measured at the end point. (**K**) Tumor-infiltrating CD8^+^ T cells in EO771 control and EO771-OTUD4–KD tumors were determined by flow cytometry. (**L**) Percentage of IFN-γ^+^ in tumor-infiltrating CD8^+^ T cells in EO771-control and EO771-OTUD4–KD tumors was shown. (**M**) T cell proliferation was determined by Ki67 staining using flow cytometry. (**N** and **O**) EO771, EO771-OTUD4-CD73^WT^, and EO771-OTUD4-CD73^V300P/I301Q^ breast cancer cells were orthotopically injected into the right fourth mammary gland of the C57BL/6 WT mice. PBS was used in control group. Tumor growth curve was plotted (**N**) and tumor weight was measured at the end point (**O**). (**P**–**S**) 4T1-hPD-L1 and 4T1-hPDL1-OTUD4, where the basal mouse PD-L1 was replaced with a human counterpart, and 4T1-hPD-L1 or 4T1-hPDL1-OTUD4 cells were orthotopically injected into the right fourth mammary fat pad and allow to grow around 100 mm^3^, followed by injection of ST80 (5 mg/kg, i.p.) 2 times/week, and PD-L1 antibody durvalumab (5 mg/kg, i.p.) 3 times. PBS and IgG control were used in control groups. Tumor growth (**P** and **R**) and survival curves (**Q** and **S**) of the mice were plotted. Data (means ± SEM) are representative of at least 2 independent experiments with 5 to 10 independently analyzed mice per group. *P < 0.05, **P < 0.01, ***P < 0.001, and ****P < 0.0001. For tumor growth statistical analysis, 2-way ANOVA followed by multiple unpaired *t* tests were performed.

To evaluate the role of OTUD4 in tumor growth in vivo, we created murine cell lines with modified OTUD4 expression. We developed OTUD4 OE and OTUD4 KD versions in both 4T1 and EO771 cells. The affect on tumor growth was then assessed using a syngeneic mouse model (Figure 6A). Engineered 4T1 control and 4T1 OTUD4–OE breast cancer cells were subcutaneously injected into the mammary fat pad of female BALB/C mice. A significant increase in both the tumor growth rate and tumor weight was observed 21 days postinjection of the tumor cells (Figure 6, B and C). Furthermore, OTUD4 overexpression resulted in an increase in membrane-bound expression levels of CD73 on tumor cells from tumor-bearing mice (Figure 6D). The data support a tumor-promoting role of the OTUD4/CD73 proteolytic axis in vivo.

Previous studies have indicated that elevated levels of CD73 are often associated with an increase in immunosuppressive cell populations such as Tregs, MDSCs, and tumor-associated macrophages (TAMs) (32, 33). To analyze the affect of OTUD4 in modulating tumor immunity, we examined the tumor immune infiltrates using a high-dimensional spectral flow cytometry (CyTEK) panel that incorporated hallmark markers for all major immune populations as described previously (34). The dimensionality reduction tool viSNE was employed to compare 4T1 control and 4T1 OTUD4–OE tumors. Live intact single cells gated from CD45^+^ tumor infiltrates could be clearly grouped into distinct subsets, including CD4^+^ T cells (CD3^+^CD4^+^), CD8^+^ T cells (CD3^+^CD8^+^), proliferating CD8^+^ T cells (CD3^+^CD8^+^Ki67^+^), CD8^+^ tissue resident memory T cells (CD69^+^CD103^+^, TRM), exhausted CD8^+^ T cells (PD-1^+^TIM3^+^, T_EX_), stem-like progenitors of exhausted CD8^+^ T cells (CD69^+^Ly108^+^, T_PEX_), Treg (CD3^+^CD4^+^ Foxp3^+^CD25^+^), non-Treg CD4^+^ T cells (CD3^+^CD4^+^ Foxp3−), B cells (CD19^+^ CD3−), dendritic cells (MHC-II^+^CD11c^+^CD11b^+^, DC), TAMs (Gr1− F4/80^+^CD11b^+^, TAM), polymorphonuclear MDSCs (Ly6G^+^ Ly6C^lo^CD11b^+^, PMN-MDSC), monocytic MDSC (Ly6G^lo^ Ly6C^+^CD11b^+^, M-MDSC), NK (CD3− NKp46^+^), and NKT (CD3^+^NKp46^+^). Frequencies of proliferating CD8^+^ T cells, NK, and NKT among CD45^+^ tumor infiltrates in 4T1 OTUD4–OE tumor-bearing mice were significantly decreased compared with those in the control mice (Figure 6E and Supplemental Figure 12A). By contrast, the frequency of immunosuppressive TAM and PMN-MDSC was increased (Figure 6E). Although there was no statistical difference in the frequency of CD8^+^ TRM, TEX, or TpEX between 4T1 control and OTUD4-OE tumors (Supplemental Figure 12B), terminal effector-like TEX were more abundant in OTUD4-OE tumors, as indicated by increased expression levels of CX3CR1, KLRG1, and CD101 with decreased levels of Ki67 (Figure 6F). We also found that OTUD4 OE in tumor cells enhanced a tumor-promoting M2-like phenotype in TAMs, usually defined by the expression of CD163 (Figure 6G). Further, there was a significant reduction in IFN-γ and TNF-α secretion by infiltrating CD8^+^ and CD4^+^ T cells in OTUD4 OE tumors (Figure 6H and Supplemental Figure 12C).

Conversely, OTUD4 KD in either 4T1 (Supplemental Figure 13, A and B) or EO771 (Figure 6, I and J) murine breast cancer cell lines hindered tumor development compared with the control group. Additionally, there was increased tumor infiltration of CD8^+^ T cells (Figure 6K) expressing higher levels of IFN-γ (Figure 6L) and Ki67 (Figure 6M) in EO771-OTUD4 KD tumor-bearing mice compared with those in the control mice. To evaluate the importance of the physical interaction between OTUD4 and CD73 for tumor growth, we overexpressed both OTUD4 and CD73^WT^ (OTUD4 OE + CD73^WT^) or OTUD4 and CD73^V300P/I301Q^ with mutated interaction sites (OTUD4 OE + CD73^V300P/I301Q^) in EO771 cells. While there was no significant difference in tumor growth between mice with EO771-OTUD4-CD73^V300P/I301Q^ and EO771 vehicle control, tumor growth was accelerated in EO771-OTUD4-CD73^WT^ tumor-bearing mice (Figure 6, N and O). The results underscore the importance of OTUD4-mediated deubiquitylation of CD73 for tumor growth.

### Pharmacological blockade of interaction of OTUD4 and CD73 promotes tumor immunogenicity and inhibits tumor progression in immune-cold breast cancer.

To determine the therapeutic relevance of our newly developed inhibitor ST80, we examined the antitumor effect of ST80 by single treatment or in combination with anti-hPD-L1 drug durvalumab, using a TNBC 4T1 model where the endogenous mouse PD-L1 was knocked out and replaced with the human counterpart (4). As illustrated in Supplemental Figure 13, C and D and Figure 6, P and Q, combining ST80 with durvalumab led to enhanced suppression in tumor growth and prolonged survival without evident mouse body weight changes. Further, similar therapeutic benefits of combination treatment were observed in mice bearing 4T1-hPDL1-OTUD4–OE tumors (Figure 6, R and S), where durvalumab monotherapy failed to suppress tumor growth compared with IgG control. These findings indicate a therapeutic value of pharmacologically targeting the proteolytic interaction of OTUD4 and CD73 to overcome the resistance of immune checkpoint inhibitors (e.g., anti-PD-L1 antibodies), especially in high CD73-expressing, immunologically suppressive TNBC. Next, we compared the antitumor efficacy of ST80 with the anti-CD73 monoclonal antibody and found that both ST80 and anti-CD73 antibodies significantly suppressed tumor progression (Supplemental Figure 13, E and F). Further, depletion of CD8^+^ T cells completely abrogated the efficacy of ST80, indicating an essential role of CD8^+^ T cells in ST80-mediated antitumor effect (Supplemental Figure 13G). To investigate whether ST80 treatment affected immunosuppressive cells like Tregs, TAMs, and MDSCs, we performed immune profiling analysis on ST80-treated E0771 tumor infiltrates, which showed that ST80 treatment caused a significant reduction of infiltration of immunosuppressive Tregs (Supplemental Figure 13H), TAMs (Supplemental Figure 13I), and PMN-MDSCs (Supplemental Figure 13J), accompanying increased infiltration of M-MDSCs (Supplemental Figure 13K). Further, ST80 treatment enhanced tumor infiltration of IFN-γ^+^ CD8^+^ T cells (Supplemental Figure 13L) and decreased their expression of PD-1 (Supplemental Figure 13M). These results were consistent fully with those from the immune characterization of OTUD4-KD (Figure 6, K–M) and -OE (Figure 6, E–H) tumor infiltrates. In addition, ST80 treatment decreased surface expression levels of CD73 and PD-L1 on tumor cells from tumor-bearing mice (Supplemental Figure 13N). The data together support a great potential of pharmacological targeting of the proteolytic interaction of OTUD4 and CD73 to mitigate tumor immune evasion and revive antitumor T cell immunity.

**Figure 7:**
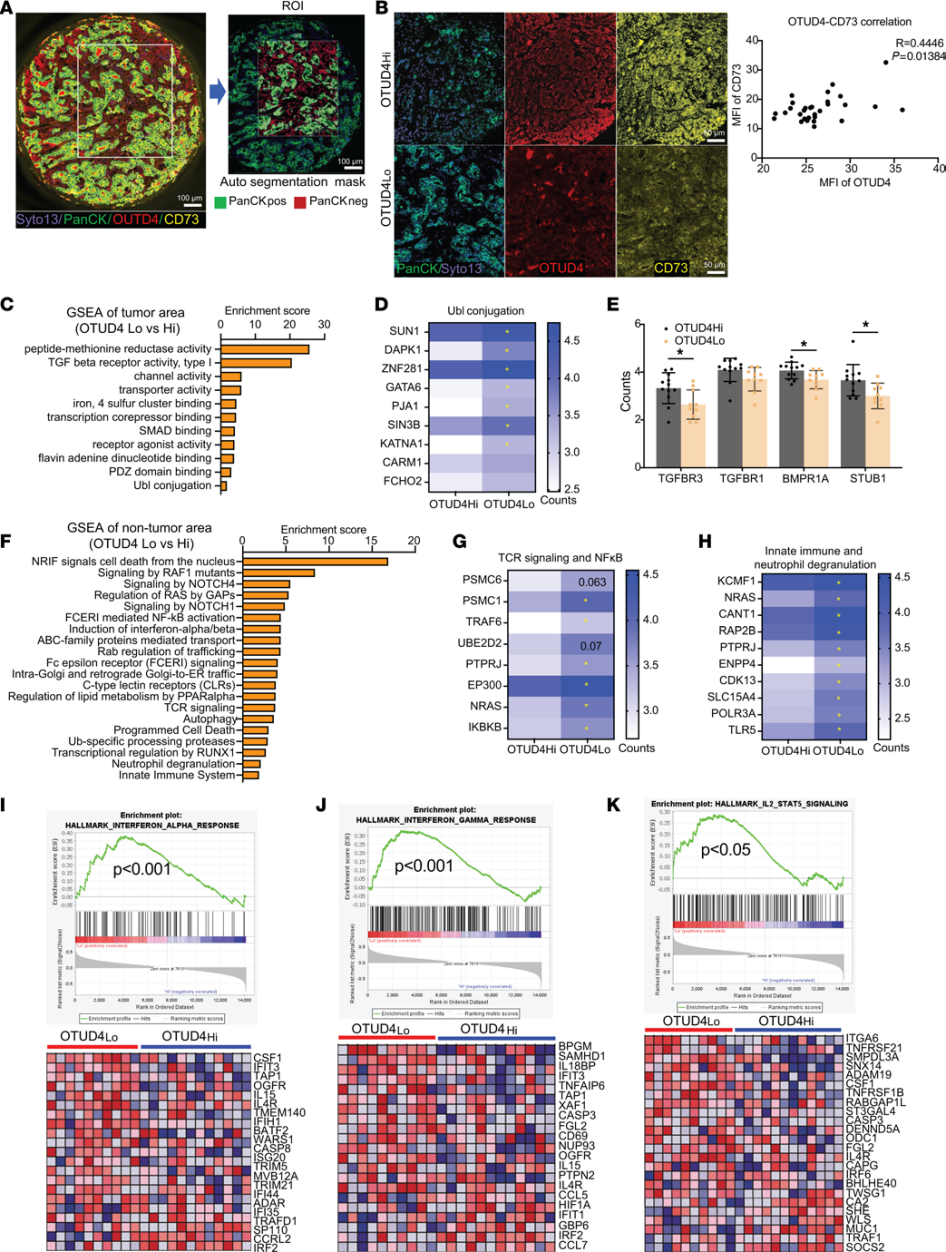
Spatially resolved signatures pertaining to OTUD4^hi^/CD73^hi^ are associated with unfavorable immune responses. (**A**) Representative staining of OTUD4, CD73 and PanCK, followed by the selection of regions of interest (ROI) and subsequent autosegmentation in TNBC tissue sections (*n* = 30) using DSP. Scale bars: 100 μm**.** (**B**) The analysis of OTUD4 and CD73 protein expression on TNBC (*n* = 30) using ImageJ showing the positive correlation between OTUD4 with CD73. Scale bars: 50 μm**.** (**C–E**) DAVID functional gene onology (GO) analysis of molecular function (MF) on the DEGs (different expressional genes) in tumor epithelial compartment (PanCK^+^) between OTUD4-high expression cases and OTUD4-low expression cases (**C**), revealing decreased Ubl conjugation (**D**) and increased TGF-β signaling activity (**E**) in OTUD4-high expression tumors; (**F**–**H**) DAVID REACTOME analysis of DEGs in nontumor areas between OTUD4-high expression cases and OTUD4-low expression cases (**F**), highlighting increased TCR signaling, NFκB activation (**G**) and induction of IFN-α/β (**H**) in patients with OTUD4-low expression; (**I–K**) Gene set enrichment analysis (GSEA) demonstrating positive enrichment of hallmark curated gene sets for IFN-α response (**I**), IFN-γ response (**J**), and IL-2-STAT5 signaling (**K**) in nontumor areas of OTUD4-low expression cases compared with OTUD4-high expression cases. Data (mean ± SEM) are representative of at least 3 independent experiments. *P < 0.05, by 1-way ANOVA with Tukey’s multiple comparisons test.

To validate the spatially correlated expression of OTUD4 and CD73 in tumor cells with immune signaling programs in the tumor microenvironment, we performed spatially indexed transcript profiling (GeoMx digital spatial profiling) of tumor tissue sections from 6 patients with TNBC. We profiled 30 regions of interest (ROIs) per tissue section and further segmented each region into epithelial versus nonepithelial areas using pan-CK staining (Supplemental Figure 14A and Figure 7A). Costaining of OTUD4 and CD73 was also used for spatial segmentation analysis. We observed a positive correlation between OTUD4 and CD73 expression in malignant epithelial areas across all regions per tumor (Figure 7B). Using gene pathway analysis (Kyoto Encyclopedia of Genes and Genomes [KEGG]/Reactome) (Figure 7, C–E) based on spatial transcriptomes, we found Ubl conjugation, TGF-β receptor activity, and SMAD binding pathways pertaining to CD73 PTM modulation were particularly enriched in OTUD4^lo^ compared with OTUD4^hi^ tumor areas, further supporting potential protein interactions between OTUD4 and CD73 in tumor cells.

Subsequently, using gene pathway analysis (Figure 7, F–H), we found TCR signaling and NFκB activation, and induction of IFN-α/γ pathways related closely to the tumor immune response were significantly enriched in the nontumor compartment from OTUD4^lo^ areas compared with those from OTUD4^hi^ areas. Likewise, Gene Set Enrichment Analysis (Figure 7, I–K) revealed the significantly enriched pathways of IFN-α response (Figure 7I), IFN-γ response (Figure 7J), and IL-2-STAT5 signaling (Figure 7K) in nontumor compartments from OTUD4^lo^ areas compared with those from OTUD4^hi^ areas. Similar results were also obtained from a comparison between CD73^lo^ and CD73^hi^ areas (Supplemental Figure 14B) or OTUD4^lo^/CD73^lo^ and OTUD4^hi^/CD73^hi^ areas (Supplemental Figure 14, C–F) in nontumor compartments across all ROIs. These findings indicate distinct immune features of the OTUD4^hi^/CD73^hi^ areas are implicated in the suppression of antitumor responses and promotion of tumor growth, supporting the role of the OTUD4/CD73 proteolytic axis in tumor immune evasion.

To determine the clinical significance of the CD73 proteolytic regulation in TNBC’s immune response and prognosis, CIBERSORT analysis (Supplemental Figure 15, A and B) showed breast tumors with heightened TRIM21 expression were characterized by an increased CD8^+^ T cell population and showed an increase in lymphocyte infiltration accompanied by an IFN-γ signature. Conversely, elevated OTUD4 expression in patients with TNBC (sourced from the GSE31519 and TCGA databases) correlated with poorer survival outcomes, including overall and event-free survival, even in the presence of abundant cytotoxic T lymphocytes (Supplemental Figure 15C). Further, we observed that patients with pronounced OTUD4 expression had a dampened IFN-γ gene signatures (Supplemental Figure 15D). Additionally, we deepened our investigation into the expression of immune-related proteins in immune-suppressive cohorts of patients with TNBC. Utilizing Spearman correlation and hierarchical clustering analyses, we found a positive correlation between OTUD4 and CD73 with PD-L1 expression. (Supplemental Figure 16).

## Discussion

This study integrates multiomic analyses to comprehensively define a distinct immunosuppressive signature in a subset of TNBCs, followed by in-depth mechanistic dissection with multidisciplinary approaches. We identified an unfavorable immune profile of tumors with CD73 high expression correlating with high OTUD4 expression in an immune-suppressive subset of TNBCs. Mechanistically, we have demonstrated that stabilization of CD73 catalyzed by OTUD4 suppresses cytotoxic CD8^+^ T cell function, leading to tumor immune evasion. We further revealed a role of TGF-β signaling in orchestrating the OTUD4/CD73 proteolytic axis that promotes tumor progression. More importantly, we have developed a pharmacologic inhibitor, ST80 that is potent to specifically inhibit the physical interaction of OTUD4 and CD73 to promote ubiquitylation of CD73, thereby causing CD73 degradation and revival of CD8^+^ T cell function. Notably, treatment with ST80 sensitized TNBC tumors to anti-PD-L1 therapy even in the case of tumors with high expression of OTUD4 and CD73. This study introduces a therapeutic paradigm by specific targeting of a proteolytic interaction for improving tumor immunogenicity and circumventing resistance to anti-PD-L1 therapy for immunotherapy nonresponsive TNBC.

### Yin-yang regulation of CD73 by ubiquitylation and deubiquitylation orchestrates tumor immunosuppression.

CD73 is well characterized as a transmembrane protein with nucleotidase enzymatic activity that catalyzes production of immunosuppressive adenosine in association with CD39 (15). CD73 has been reported to be in the plasma membrane, cytosol, and nucleus (35, 36). We also visualized cytosolic CD73 abundance within tumor cells. Regulation of CD73 has been so far limited to the control on a transcriptional level, whereas fluctuation of CD73 is achieved in response to several signaling molecules, including STAT3, HIF-1α, TGF-β, TNF-α, and interferons (35–38). Nevertheless, little is known about CD73 expression regulated by PTM. This study demonstrates a sophisticated yin-yang regulation of CD73 by a specific proteolytic system with TRIM21-mediated destruction and OTUD4-catalyzed stabilization. Our results suggest the membrane abundance of CD73 is determined by a capacity of cytosolic reservoir. Yin-yang regulation of CD73 by E3 ligase TRIME21 and deubiquitinase OTUD4 serves as a bottle neck, buffering the stream of CD73 that localizes to the cellular membrane. Increased CD73 deubiquitylation by OTUD4 due to etiological factors enhances function for membrane-bound CD73, conferring tumor immune evasion. Thus, inhibiting CD73 deubiquitylation could decrease the intracellular CD73 reservoir in tumor cells, therefore restricting the adenosinergic effects through the shrinking of the CD73 bottle neck. Our molecular dynamic analyses create a 3D model for featuring a coordination between TRIM21-mediated CD73 ubiquitylation and OTUD4-guided deubiquitylation. The underlying details of interplay between TRIM21 and OTUD4 in determining cellular CD73 abundance modulating tumor immune responses in the context of tumor progression awaits further investigation.

### Hijack of OTUD4 by TGF-β antagonizes IFN-γ/TRIM21 cascade, stabilizing CD73 and inhibiting tumor immunogenicity.

The homeostatic status of CD73 expression is important in maintaining an immunosuppressive response. This study demonstrates that CD73 is a fast-turnover protein whose half-life is governed by the ubiquitin-proteosome system. The equilibrium of basal cellular levels of CD73 protein is dictated by E3 ligase TRIM21. Under aberrant circumstances, uncontrolled OTUD4 counteracts TRIM21-mediated ubiquitylation by removing ubiquitin chains from CD73, thereby stabilizing CD73. Our bioinformatic analyses revealed IFN-γ and TGF-β are a pair of upstream nodes counteracting signaling that coordinate balance of CD73 in immunosuppression. Results from TCGA analyses uncovered a positive correlation between high TRIM21 expression and increased gene signatures related to the IFN-γ pathway in patients with TNBC, consistent with the laboratory observation of elevated TRIM21 activity in response to IFN-γ. Intriguingly, previous studies have shown that an IFN-γ effect could be compromised by the TGF-β signaling in immunosuppressive environments (39–41). Tumor microenvironment often exhibits increased TGF-β levels, which suppresses antitumor immune responses by inhibiting the activities of effector T cells and NK cells while enhancing functions of Tregs and MDSCs (42, 43). Further data analyses indicated a signature of high CD73 expression with increased TGF-β signaling in the context of TNBC. Further, our GSEA analyses showed correlation between high OTUD4 expression and increased TGF-β signaling activity, implying a cascade of TGF-β/OTUD4/CD73 in maintaining a tumor-promoting immune microenvironment. This study suggests that hijack of OTDU4 by TGF-β antagonizes the IFN-γ/TRIM21cascade, thereby stabilizing CD73 and inhibiting tumor immunogenicity.

### ST80, a potent pharmacologic inhibitor, blocks OTUD4/CD73–mediated adenosine production and restores CD8^+^ cytotoxic T cell function in immune-suppressive TNBCs.

CD73 has been considered as a potential target for cancer therapy in recent years. Several strategies have been developed to target CD73 in cancer treatment, including antibody blockade as well and inhibition of its receptor A2R2 (36, 44). Currently, numerous anti-CD73 antibodies are in various stages of preclinical and clinical development, and some have shown promising results in early phase clinical trials (45, 46). However, each approach has its own set of challenges and limitations. In this context, targeting the protein-protein interactions between CD73 and its upstream deubiquitinase OTUD4 in destabilizing CD73 and reducing its cell surface abundance provides a therapeutic rationale. In addition, targeting interaction between OUTD4 and CD73 largely decreases the issue of specific targeting for deubiquitinase and potential of drug resistance for CD73. Here, we developed ST80 as a potent small molecule inhibitor that efficiently intercepts binding between OUTD4 and CD73, significantly minimizing both cytosolic and membrane-bound CD73 abundance. In both in vitro and in vivo settings, ST80 inhibits immune-suppressive TNBC cell and tumor growth by reviving cytotoxic CD8^+^ T cell responses. Comprehensive characterization of ST80 in improving current cancer immunotherapeutics awaits further investigation.

### The antitumor effects of combined treatment with PD-L1 blockade are exacerbated by ST80.

A critical challenge in cancer immunotherapy research is to develop more accurate preclinical models that may translate to humans. In this work, to evaluate the antitumor effects of combined ST80 treatment in combination with an FDA-approved anti-hPD-L1 antibody (i.e., durvalumab), we have utilized a mouse orthotopic tumor model of 4T1-hPD-L1, where the basal mouse PD-L1 gene was replaced with a functional human counterpart. Ideally, a “humanized” mouse model, by knocking in human immunologic genes is a suitable tool to evaluate novel cancer therapies in combination with human specific immunotherapeutics. However, this “humanized” system has drawbacks resulting from incompatibilities between the 2 species, including immune responses against xenoantigens. Our results thus need to be further validated by more physiologically relevant models for future translation. In summary, this study reveals what we believe to be a previously unidentified role for the OTUD4/CD73 proteolytic axis in determining tumor immune evasion in TNBC. CD73 protein stability is regulated by a reciprocal interplay between ubiquitination and deubiquitylation, which subsequently dictates tumor immune responses. Targeting a specific interaction between tumor OTUD4 and CD73 to efficiently destabilize CD73 is an effective strategy for TNBC treatment, especially when combined with PD-L1 blockade, even in the case with abundant expression of tumor CD73 and OTUD4.

## Methods

### Sex as a biological variable.

Our study exclusively examined female mice because the disease modeled is only relevant in females.

### Animal models.

BALB/C and C57BL/6 mice (8-week-old female) were purchased from Charles River Laboratories. Engineered 4T1 EO771 control and related ShOTUD4 or OTUD4 stable expression breast cancer cells (2 × 10^6^) were injected subcutaneously into the mammary fat pad of female BALB/C mice on day 0. EO771-OTUD4, EO771 OTUD4-CD73, and EO771 OTUD4-CD73-V300P/I301Q breast cancer cells (2 × 10^6^) were injected subcutaneously into the mammary fat pad of female C57BL/6 mice on day 0. Tumor volumes were measured along 3 orthogonal axes (a, b, and c) and calculated as (a×b×c)/2 every 2–4 days. The tumor weight was determined at endpoint.

### Cell lines and chemical reagents.

HEK-293T, Human Mammary Epithelial Cells, 15 human breast cancer cell lines [American Type Culture Collection Breast Cancer Cell Panel (ATCC 30–4500 K)], 4T1, EO771, HCC827, SKOV3, and HCT116 were obtained from the ATCC. Normal (nonimmortalized) human mammary epithelial cells (passage 2–5) were cultured in Mammary Epithelial Cell complete medium (basal medium plus growth kit) and maintained in a humidified 37°C incubator with 5% O_2_, CO_2_, and N_2_. Other cancer cell lines were maintained as per the manufacturer’s protocol. For TGF-β stimulation, cancer cell lines were treated with TGF-β (3 ng/mL) at different time points. All chemical reagents and antibodies used in this study are listed in Supplemental Table 1.

### Statistics.

Unless specified, results are expressed as mean ± SEM. Experiments were performed at least twice unless otherwise specified. Statistical analyses were performed using GraphPad Prism (ver.8, GraphPad Software) or R software (version, 3.3.3). The significance of the differences in the assays was analyzed by Student’s *t* test or 1 or 2-way ANOVA, followed by Tukey’s multiple comparisons test. Comparison of survival curves or tumor-free curve was performed using Log-rank (Mantel-Cox) test. Pearson correlation or Spearman correlation was used to study the correlation between 2 molecules. A value of *P* < 0.05 was considered significant.

### Study approval.

All experimental procedures for animals were approved by IACUC of Emory university and IACUC of Northwestern University.

### Data availability.

All data associated with this study are presented in the paper or in the Supplemental Materials. The proteomic data discussed in this publication are publicly accessible through Breast cancer proteomic data can be accessed through National Cancer Institute Clinical Proteomic Tumor Analysis Consortium (https://proteomic.datacommons.cancer.gov/pdc/; accession number: PDC000173). TNBC immune-related mRNA data can be accessed through Gene Expression Omnibus database (accession number: GSE88847). The single-cell BC data Gene can be access through Expression Omnibus database (accession number: GSE173634). Immune analysis using CAMOIP, TIMER2 and TIDE online platform are available at https://www.camoip.net, http://timer.cistrome.org and http://tide.dfci.harvard.edu/login/ A Supporting Data Values file is also provided.

## Author contributions

YZ was responsible for conceptualization, formal analysis, supervision, investigation, methodology, drug discovery, and writing, review, and editing of the manuscript. AB was responsible for structural modeling and docking simulation analyses, virtual screening, and writing, review, and editing of the manuscript. PX was responsible for study of preclinical models and writing, review, and editing of the manuscript. AAI was responsible for bioinformatic analyses and writing, review, and editing of the manuscript. AU was responsible for drug discovery and preclinical diseases models. QJ was responsible for construction for plasmids, protein analysis, and writing, review, and editing of the manuscript. JJC was responsible for tumor-immune cell coculture analyses. LZ was responsible for immune-staining and colocalization. JYL was responsible for structural modeling and docking simulation analyses and virtual screening. YX was responsible for bioinformatics. XL was responsible for bioinformatics. MC was responsible for clinical data analysis and writing, review, and editing of the manuscript. WJG was responsible for clinical data analysis and writing, review, and editing of the manuscript. CJH was responsible for tumor immune response analysis. TWG was responsible for writing, review, and editing of the manuscript. MAB was responsible for clinical data analysis and writing, review, and editing of the manuscript. KK was responsible for clinical data analysis and writing, review, and editing of the manuscript. HF was responsible for bioinformatics, drug validation, and writing, review, and editing of the manuscript. IB was responsible for conceptualization, molecular dynamic analyses, drug development, and writing, review, and editing of the manuscript. BZ was responsible for conceptualization, data curation, supervision, funding acquisition, and writing, review, and editing of the manuscript. YW was responsible for conceptualization, drug discovery, data curation, supervision, funding acquisition, project administration, and writing, review, and editing of the manuscript. YZ, AB, and PX contributed equally to this work.

## Supplementary Material

Supplemental data

Unedited blot and gel images

Supporting data values

## Version 1 03/26/2024

In-Press Preview

## Version 2 05/15/2024

Electronic publication
