# Supplementary material for: Pharmacological suppression of the OTUD4/CD73 proteolytic axis revives antitumor immunity against immune-suppressive breast cancers
Source: J Clin Invest. 2024 Mar 26;134(10):e176390. doi: 10.1172/JCI176390 (PMC11093616; doi:10.1172/JCI176390)
Supplement: Supplemental data [file jci-134-176390-s093.pdf]

Pharmacological Suppression of the OTUD4-CD73 Proteolytic Axis Revives Antitumor Immunity against Immune-Suppressive Breast Cancers

Yueming Zhu[1,2†], Anupam Banerjee[3,4†], Ping Xie[5†], Andrey A. Ivanov[1,2,6], Amad Uddin[1], Qiao Jiao[1], Junlong Chi[1,7], Lidan Zeng[1], Ji Young Lee[3], Yifan Xue[8], Xinghua Lu[8], Massimo Cristofanilli[9], William J Gradishar[5], Curtis Henry[2,10], Theresa W. Gillespie[2,11,12], Manali Ajay Bhave[2,12], Kevin Kalinsky[2,12], Haian Fu[1,2,6], Ivet Bahar[3,4*], Bin Zhang[5*] and Yong Wan[1,2,12*]

Supplementary Figures1-16

Supplementary Methods

Figure S1. Elevated expression of CD73 in TNBC correlates with a poor tumor immunity.

(A) mRNA analysis of immune-relevant proteins in 37 TNBC patient specimens from the GEO database, the genes (rows) are sorted according to the difference between the average mRNA levels in breast cancer types. **(B-D)** Single-cell CD73 expression profiles of 35,276 cells from 32 cell lines (B) color-coded according to cancer subtype (C) (luminal A, luminal B, Her2, TNBC-A, TNBC-B) with brown indicating CD73 expression levels in the atlas (D).

Figure S2. Elevated expression of CD73 in TNBC correlates with a poor tumor immunity. **(A)** Go-BP pathway analysis using TCGA dataset identifying the most affected pathways between CD73 high vs CD73 low breast cancer group. **(B)** CIBERSORT analysis using TCGA-BRCA dataset showing that high CD73 levels correlate with decreased $CD8^+$ T cell populations. **(C-D)** Breast cancer specimens TMA stained for CD73 and CD8 (C) and quantified staining intensity correlation between CD73 and CD8 (D). **(E-G)** Gene Set Enrichment Analysis (GSEA) enrichment score curves of NT5E (CD73) high vs NT5E (CD73) low. The y-axis represents enrichment score (ES) and on the x-axis are genes (vertical black lines) represented in gene sets. The green line connects points of ES and genes. ES is the maximum deviation from zero as calculated for each gene going down the ranked list and represents the degree of over-representation of three gene sets: negative regulation of immune response (E), negative regulation of T cell activation (F) and negative regulation of T cell proliferation (G) at the top or the bottom of the ranked gene list. *$p < 0.05$, ***$p < 0.001$, and ****$p < 0.0001$, by multiple unpaired T-test.

Figure S3. Elevated expression of CD73 in TNBC correlates with a poor prognosis.

(A-B) Immune Score analysis using breast cancer TCGA cohort showing NT5E (CD73) positively correlated with decreased tumor immunity (A) related pathway and genes (B). **(C)** Univariable cox regression analysis showing high NT5E (CD73) expression as an increased survival hazard in breast cancer patient overall survival. **(D)** Increased accumulation of CD73 levels correlates with poor outcomes in patients with TNBC overall survival with Kaplan-Meier plotting. ****p < 0.0001, by multiple unpaired T-test.

Figure S4. OTUD4-guided CD73 deubiquitylation leads to CD73 stabilization.

(A-D) Validation of biochemical interaction between CD73 and OTUD4 in HCC827 (A-B), SKOV3 (C), and HCT116 (D) cells by coimmunoprecipitation of endogenous CD73 and by co-immunoprecipitation of endogenous OTUD4. **(E)** Triple immunofluorescence staining CD73 with OTUD4 in MDA-MB231, MDA-MB468, HCC827, and HCT116 cells followed by the confocal microscope. Colocalization between CD73 and OTUD4 and DAPI was measured by immunofluorescence staining, showing a principal overlap of CD73 (green) and OTUD4 (red) in the cytosol. **(F)** Slides of MDA-MB231 cells were incubated with mouse anti-CD73 and rabbit anti-OTUD4 antibodies or antibody diluent alone. Duolink PLA was then performed and red dots indicate the interaction of the two proteins. **(G)** The MFI of membrane expressed CD73 was determined by flow cytometry in both MDA-MB231 and MDA-MB231-OTUD4 cells. **(H)** immunofluorescence staining CD73 in MDA-MB468-OTUD4 cells followed by the confocal microscopy. **(I)** The MFI of membrane expressed CD73 was determined by flow cytometry in both MDA-MB468 and MDA-MB468-ShOTUD4 cells. Data (mean ± SEM) are representative of at least three independent experiments. $^{**}p < 0.01$, $^{***}p < 0.001$, by unpaired T-test.

Figure S5. OTUD4-guided CD73 deubiquitylation affects tumor immune response.

(A) The adenosine levels were determined in MDA-MB231-CD73^{WT}, MDA-MB231-CD73^{WT}-OTUD4 and MDA-MB231-CD73^{WT}-shOTUD4 cells. ****p < 0.0001. **(B-C)** The AMP levels were determined in MDA-MB468-ShOTUD4 (B) and MDA-MB468-OTUD4 OE (C). **(D-E)** The ADA activities were determined in MDA-MB468-ShOTUD4 (D) and MDA-MB468-OTUD4 OE (E). **(F-G)** MDA-MB468, MDA-MB468- ShOTUD4 (F), and MDA-MB468-OTUD4 OE (G) were cocultured with human PBMCs with or without APCP treatment, and CD8+ IFNγ+ T cell population was measured and quantified using flow cytometry. Represented flow cytometry histograms of the percentage of CD8+ T cells expressing IFN-γ were shown. **(H-I)** MDA-MB231, MDA-MB231-ShOTUD4 and MDA-MB231-OTUD4 OE were cocultured with human PBMC with or without APCP treatment, Ki67 in CD8+ cell population was measured and quantified using flow cytometry. Data (mean ± SEM) are representative of at least three independent experiments. *p < 0.05 and ****p < 0.0001, by 1-way ANOVA with Tukey's multiple comparisons test and unpaired T-test.

Figure S6. Intermolecular inter-residue interactions between CD73 with OTUD4 regulate tumor immune response.

(A-B) Schematic diagram of human OTUD4 domains and strategy to engineer a series of OTUD4 deletion mutants. Mapping of the molecular domain on OTUD4 involving in the interaction with CD73. The interactions between CD73 and OTUD4 fragments were examined by co-IP experiments in HEK-293T cells. Amino acid stretches from 385-449 on OTUD4 (B) were identified as the region that mediates the interaction between OTUD4 and CD73. **(C)** CD73 (green) interacts with the PRY/SPRY domain of TRIM21 (cyan) at interface spanning residues G175-M225 (red). The top inset shows the coevolving residues V80 (red spheres)-T198 (cyan spheres) and H116 (cyan spheres) - I215 (red spheres) at the TRIM21-CD73 interface. The bottom right panel shows two salt bridges at the interface: K104 -D205 and H116 - E203. The complex selected from amongst 130 docked complexes (90 generated using ClusPro1 and 40 generated using HADDOCK2) has an interaction interface of 1,472.2 Å2 as computed using NIP/NSC3, and an interaction energy of -17.0 kcal/mol as computed by PRODIGY and has 19 hydrogen bonds5 at the interacting interface). **(D)** TRIM21 PRY/SPRY domain share the same interface (yellow spheres) with CD73 as it shares with IGG FC6 (green). **(E)** Ensemble analysis of CD73-OTUD4 complexes generated by docking simulations. Most frequent interfacial interactions occur between residues Y286-D311 of CD73 and R380-N410 of OTUD4 based on the analysis of 35 shortlisted complexes. These complexes have at least 50% of CD73 residues V275-D311 and 50% of OTUD residues R380-N410 lying at the interface. The individual numbers of occurrence of residues of CD73 and OTUD4 at the interface and associated numbers of hydrogen bonds, salt bridges and hydrophobic interactions are indicated along the vertical (CD73) and horizontal (OTUD4) arrays. **(F)** Heat map showing the interaction energy between CD73 and OTUD4 for all possible single amino acid substitutions at V300 (ordinate) and I301 (abscissa). The binding energy corresponding to the WT proteins is highlighted in green. The most destabilizing coupled mutations at V300 and I301 are highlighted in yellow. The table underneath summarizes the ΔG_{B}

and $\Delta\Delta G_f$ (corresponding to the respective changes in binding and folding free energies) predicted for the most destabilizing double mutations at V300 and I301. **(G)** Heat map showing the difference in folding free energy ($\Delta\Delta Gf$) of CD73 as predicted by FoldX for all possible single amino acid substitutions at V300 (ordinate) and I301 (abscissa). The $\Delta\Delta Gf$ corresponding to the wt protein is taken as 0 and highlighted in *green*. The $\Delta\Delta Gf$ of CD73 for the coupled mutations at V300 and I301 is -5.2 kcal/mol and is highlighted in *yellow*. **(H)** Validation of interaction domains between OTUD4 with CD73 by coimmunoprecipitation of ectopic V5-tagged CD73 and V5 tagged CD73 V300P/I301Q mutant in MDA-MB468 cells. The ubiquitylation status of CD73 and CD73 V300P/I301Q mutant were also determined using an antibody against ubiquitin. **(I)** The adenosine levels were determined in MDA-MB231-CD73^{WT}, MDA-MB231- CD73$^{V300P/I301Q}$ cells. **(J)** The adenosine levels were determined in 4T1-CD73^{WT}, 4T1- CD73$^{V300P/I301Q}$ cells. **(K)** The adenosine levels were determined in EO771-CD73^{WT}, EO771- CD73$^{V300P/I301Q}$ cells. **(L)** MDA-MB468, MDA-MB468-CD73^{WT} and MDA-MB468-CD73$^{V300P/I301Q}$ were cocultured with human PBMCs and percentage of Ki67$^+$ in CD8$^+$ T cell population quantified using flow cytometry. Data (mean ± SEM) are representative of at least three independent experiments. *p < 0.05, **p < 0.01, ***p < 0.001 and ****p < 0.0001, by multiple unpaired T-test.

Figure S7. IFN-γ and TGF-β signaling highly correlated with CD73 proteasomal-ubiquitylation degradation.

(A) Gene Set Enrichment Analysis (GSEA) enrichment score curves of TRIM21 high vs TRIM21 low. The y-axis represents enrichment score (ES) and on the x-axis are genes (vertical black lines) represented in gene sets. The green line connects points of ES and genes. ES is the maximum deviation from zero as calculated for each gene going down the ranked list and represents the degree of over-representation of three gene sets.

Figure S8. TGF-β signaling modulating CD73 proteasomal-ubiquitylation degradation.

(A) MDA-MB231 were treated with 3ng/mL TGF-β, OTUD4 and CD73 protein levels were determined by immunoblotting. **(B)** MDA-MB468 and MDA-MB231 were treated with 3ng TGF-β, OTUD4 levels were determined by immunofluorescence staining, quantification of the fluorescence intensity was calculated and present on the right. **(C)** MDA-MB231 cells were treated with 3ng TGF-β, adenosine production was determined. **(D)** MDA-MB468-ShOTUD4 and MDA-MB231-ShOTUD4 cells were treated with 3ng/mL TGF-β, OTUD4 and CD73 protein levels were determined by immunoblotting. **(E)** MDA-MB231-ShCon and MDA-MB231-ShOTUD4 cells were treated with 3ng/mL TGF-β, adenosine production was determined. **(F)** In MDA-MB468 cells, TGFBR1 was knocked down, or the cells were treated with LY2109761. The interaction between OTUD4 and CD73 was determined by immunoprecipitation. **(G)** MDA-MB231-CD73^{WT} and MDA-MB231-CD73$^{V300P/I301Q}$ cells were treated with 3ng/mL TGF-β, adenosine production was determined. Data (mean ± SEM) are representative of at least three independent experiments. $^{**}p < 0.01$ and $^{****}p < 0.0001$, by unpaired T-test, 1-way ANOVA with Tukey's multiple comparisons test.

Figure S9. Detailed flowchart illustrating the small molecule selection framework.

(A) The pipeline comprises three major steps: druggability simulations using DruGUI (top left), identification of high affinity binding sites of probes (*top middle*) and pharmacophore modeling (*top right*) using Pharmmaker, extending pharmacophore modeling (bottom right) and virtual screening using Pharmit[11] (*bottom middle and bottom left*). **(B-C)** Chemical structures of the 9 small molecules identified by druggability simulations (B) and pharmacophore modeling and their binding poses in CD73 (C). **(D)** Schematic diagram of the small molecule selection framework: druggability simulations using DruGUI, identification of high affinity binding sites of probes and pharmacophore modeling using Pharmmaker and, virtual screening using Pharmit. Chemical structures of the 6 candidate small molecules identified by druggability simulations and pharmacophore modeling.

Figure S10. Protein-protein interaction inhibitor ST80 and Z22 disrupted OTUD4-CD73 interaction promote tumor immune response.

(A-B) MDA-MB231-CD73$^{V300P/I301Q}$ (A) and MDA-MB468-CD73$^{V300P/I301Q}$ (B) were treated with 0.5μM screened compounds and CD73 expression levels were determined by immunoblotting. **(C)** Mechanisms of inhibition of ST80. ST80 binds a global hinge region of CD73 interfering with the conformational mechanics of the enzyme. The curve displays the normalized displacements of CD73 residues along a soft mode predicted by the GNM. The red stars indicate global hinge sites, which overlap or closely approximate the residues that closely coordinate ST80 (3.5Å cutoff between all pair atom distance). **(D)** Heatmap showing the cross-correlation between P264, I266, R273 and K274, coordinating Z22 and those at the binding epitope of OTUD4 (V275-D311), as predicted by the softest three ANM modes. The row and column averages are shown along the two axes. P264 and I266 exhibit strong correlations with residues at the binding epitope with OTUD4 suggesting a potential allosteric effect upon complex formation. **(E)** MDA-MB468 were treated with 0.5μM ST80 or Z22 and CD73 protein turnover rate was determined by pulse-chase analysis with CHX and MG132 administration. **(F-G)** MDA-MB231 cells (F) and MDA-MB468 cells (G) were treated with different dose of ST80 or Z22 (10nM, 50nM, 100nM, 500nM and 1000nM) together with 3ng TGF-β for 24 hours and CD73 protein levels were determined by immunoblotting. **(H)** MDA-MB468 cells were treated with 0.5μM ST80 or Z22, cell lysates were collected at different time point (4, 8, 12, 24 and 48 hours) and CD73 protein levels were determined by immunoblotting. **(I)** MDA-MB231 cells and MDA-MB468 cells were treated with 0.5μM ST80 or Z22 for 24 hours and cells were counted and replating for clonogenicity survival. **(J)** MDA-MB468 cells were cocultured with human PBMCs at different Effector (E) to target (T) ratio (2:1 or 4:1) and treated with ST80 and Z22 (0.5 μM), cell proliferation rate was measured by % confluence. **(K-L)** MDA-MB468 cells were treated with 1μM, 0.5μM, and 0.1 μM ST80 (K) and Z22 (L), adenosine productions were determined. **(M-N)** MDA-MB231 cells were cocultured with human PBMCs and

treated with 0.5μM ST80 or Z22, percentage of Ki67$^+$ in CD8$^+$ T cells (M) and percentage of TNFα^+ in CD8$^+$ T cells (N) were measured and quantified using flow cytometry. Data (mean ± SEM) are representative of at least three independent experiments. *$p < 0.05$, **$p < 0.01$, ***$p < 0.001$ and ****$p < 0.0001$, by 1-way ANOVA with Tukey's multiple comparisons test.

Figure S11. ST80 disrupted OTUD4-CD73 interaction enhancing anti-tumor immune responses across various tumor contexts.

(A) HCC827 cells were treated with different dose of ST80 (10nM, 50nM, 100nM, 500nM and 1000nM) together for 24 hours and CD73 protein levels were determined by immunoblotting. **(B-J)** HCC827 (B-D), SKOV3 (E-G) and HCT116 (H-J) cells were cocultured with human PBMCs and treated with 0.5μM ST80, percentage of $IFN\gamma^+$ in $CD8^+$ T cells, percentage of $TNF\alpha^+$ in $CD8^+$ T cells, and percentage of $Ki67^+$ in $CD8^+$ T cells were measured and quantified using flow cytometry. **(K)** MDA-MB468 cells were cocultured with human PBMCs at different Effector (E) to target (T) ratio (3:1) and treated with ST80 (0.5μM), cell proliferation rate was measured by %confluence. Data (mean ± SEM) are representative of at least three independent experiments. $^*p < 0.05$, $^{***}p < 0.001$ and $^{****}p < 0.0001$, by unpaired T-test and 1-way ANOVA with Tukey's multiple comparisons test.

Figure S12. Gating strategies.

(A) Gating strategies for the panels of CyTEK spectral flow cytometry and assessment of cytokines in tumor infiltration lymphocytes. **(B)** Summarized frequencies of total infiltrated main immune populations among live CD45+ cells were compared in 4T1 control tumor and 4T1 tumor with OTUD4 over-expression at 18 days after tumor challenge. **(C)** Gating strategies for the panels of CyTEK spectral flow cytometry and assessment exhaustion in tumor infiltration lymphocytes. Data (mean ± SEM) are representative of at least three independent experiments.

Figure S13. OTUD4-mediated deubiquitylation of CD73 regulates tumor immune evasion and affects therapeutic efficacy *in vivo.*

(A-B) 4T1 control and 4T1-OTUD4 KD tumors were harvested 21 days after tumor challenge and analyzed. Tumor growth curve was plotted (A) and tumor weight (B) was measured at the end point. **(C-D)** Schematic diagram of 4T1-hPD-L1 and 4T1-hPDL1-OTUD4, where the basal mouse PD-L1 was replaced with a human counterpart, and 4T1-hPD-L1 or 4T1-hPDL1-OTUD4 cells were orthotopically injected into the left fourth mammary fat pad and allow to grow around 100 mm^3, followed by injection of ST80 (5 mg/kg, i.p.) for two times/week, and PD-L1 antibody durvalumab (5 mg/kg, i.p.) for 3 times. PBS and IgG control were used in control groups (C). The mice body weights were monitored (D). **(E-F)** Tumor growth curves (E) and tumor weight analysis (F) of EO771 tumors comparing the effects of ST80 treatment with anti-CD73 antibody treatment in C57BL/6 mice. **(G)** Effect of ST80 treatment with or without concurrent depletion of CD8 T cells by anti-CD8 antibodies in EO771-bearing C57BL/6 mice. (**H-N**) Immune profiling analysis on ST80-treated EO771 tumor infiltrates by flow cytometry. The percentage of Treg (H), TAM (I), and PMN-MDSCs (J), M-MDSCs (K), IFNγ+ CD8 T cells (L), PD-1 (M) and surface expression levels of CD73 and PD-L1 on tumor cells (N) were determined. Data (means ± SEM) are representative of at least two independent experiments with five to ten independently analyzed mice per group. *P < 0.05, **P < 0.01, ***P < 0.001, and ****P < 0.0001. For tumor growth statistical analysis, two-way ANOVA followed by multiple unpaired T test were performed.

Figure S14. The signature of OTUD4^{low}CD73^{low} is associated with favorable immune response.

(A) The experimental flow diagram of spatial profiling using GeoMx® Digital Spatial Profiler (DSP). **(B)** Gene set enrichment analysis (GSEA) showing the Hallmark IL-2 STAT5 signaling, Hallmark inflammatory response and Hallmark TNFα Signaling Via NF-κB are positively correlated with CD73-low expression in the breast cancer cases. **(C-D)** DAVID REACTOME analysis of DEGs in the non-tumor compartment between OTUD4-High plus CD73-High expression cases and OTUD4-low plus CD73 low expression cases showing increased downstream TCR signaling and interferon signaling in patients with low expression of both OTUD4 and CD73; **(E-F)** Gene set enrichment analysis (GSEA) showing positive enrichment of Hallmark_IL-2_STAT5 signaling (E) and Reactome interleukin 15 signaling (F) in non-tumor compartments of OTUD4-low plus CD73-low expression cases as compared to OTUD4-high plus CD73-high expression. Data (mean ± SEM) are representative of at least three independent experiments.

Figure S15. The signature of OTUD4^{high}/CD73^{high} is associated with unfavorable immune response.

(A) CIBERSOFT analysis using TCGA-BRCA dataset showing that high TRIM21 levels correlates with favorable immune response as measured by tumor lymphocytes levels. **(B)** Immune Score analysis using breast cancer TCGA cohort showing TRIM21 positively correlated with tumor immunity. **(C)** Kaplan-Meier curves using multivariable Cox proportional hazard model for the corresponding $CD8^+$ T cells and OTUD4 expression. The infiltration and expression level are divided into low and high levels in TCGA breast cancer patient cohort. **(D)** Correlation analysis of TRIM21-high expression vs OTUD4-high TNBC expression breast cancer samples in interferon gamma pathway analysis. The correlation coefficient is labeled from blue to red. Blue represents negative correlation, and red represents positive correlation. *$p < 0.05$, **$p < 0.01$, ***$p < 0.001$, and ****$p < 0.0001$, by multiple unpaired T-test.

Figure S16. The OTUD4-CD73 axis is positively associated PDL1 expression in a subgroup of TNBC patients.

Heatmap displaying hierarchical clustered Pearson's correlation of 309 immune related protein expression with each other in CPTAC TNBC patient cohort. Dark-red fields denote strong positive and dark-blue fields strong negative correlations between proteins.

SUPPLEMENTAL METHODS

Datasets and Bioinformatics Analysis

We analyzed several public datasets related to breast cancer using established bioinformatics methods. Proteomics data were provided by TCGA BRCA samples (1). The samples are grouped by their PAM50 subtypes. The genes were clustered according to the difference between the average proteome profiles in TNBC subtypes and those in other samples. GEOquery package was used with the accession number GSE88847 (for bulk tumor gene expression) in the GEO database to retrieve data on 37 treatments naïve TNBC patients. The TNBC subgroups (referred to as TIME subtypes) based on differential localization of $CD8^+$ T cells were requested from the author (2). Gene expression levels corresponding to the listed immune-related proteins were analyzed and heatmap were generated. We evaluated Pearson's correlation coefficients to assess the significance of the correlations between gene expression values in TNBC samples. For the hierarchical clustering of enriched terms, we employed the average linkage method and visualized the correlation coefficients in a heatmap. Patient survival analysis and expression analysis were conducted in Python based on the TCGA PacCancer Atlas data sets available through the NCI GDC Data Portal. The immune infiltration analysis, immunogenicity analysis and pathway enrichment analysis were done by using TIMER2.0, TIDE or CAMOIP web-based analysis servers (3-5). Pathway and functional enrichment analyses were performed using the Molecular Signature Database (MSigDB) (6), KEGG pathway (7), GO (8), and Reactome gene sets (9). All software used in the present study are available in the Python Package Index (PyPI) (https://pypi.org/) and/or other relevant Python repositories.

Plasmids and Transfection

The full-length and deletion mutant constructs of CD73 were generated by PCR amplification of the full-length or partial coding sequence of human CD73 and subsequently cloned into mammalian expression vectors with Flag or hemagglutinin (HA) tag. OTUD4 full-length and

deletion mutant constructs were generated by PCR amplification and then cloned into mammalian expression vectors with V5-tag. The OTUD4 deletion mutant constructs were generated using the site-directed mutagenesis kit (Agilent) as described previously (10) and cloned into mammalian expression vectors with V5-tag. Three OTUD4 constructs with respective amino acids (AA) deletions at the sequence positions 380-410, 410-440, and 440-470 were constructed as follows: (AA380-410 deletion mutant: Forward: 5'-atcagcactacctcctcttagccggacacctt-3' Reverse: 5'-aaggtgtccggctaagaggagg tagtgctgat-3' ; AA410-440 deletion mutant: Forward: 5'-ctggggaaaggcctttgtgctcactggagaat-3', Reverse: 5'-attctccagtgagcacaaaggcctttccccag-3'; AA440-470 deletion mutant: Forward: 5'-tggaaagggctggatagttagattctcgacttgtgtga-3', Reverse: 5'-tggaaagggctggatagttagattctcgacttgtgtga-3'. ShRNAs of OTUD4 were purchased from Sigma and listed as follows, for human: pLKO.1-sh-hOTUD4-1# (TRCN0000134411) and pLKO.1-sh-hOTUD4-2# (TRCN0000138670); for mouse: pLKO.1-sh-mOTUD4-1# (TRCN0000252253) and pLKO.1-sh-mOTUD4-2# (TRCN0000252254). CD73 plasmids and blank vector were purchased from Sino biological (Human: HG10904-UT, Mouse: MG50231-UT). Human OTUD4 plasmid was purchased from Addgene (22594). Mouse OTUD4 plasmid was purchased from Genscript (OMu41750). HEK-293T, MDA-MB231, MDA-MB468 cells were transfected with plasmids at a confluence of 50%-70% using Lipofectamine 2000 (Invitrogen).

Purification of CD73 Complex and Mass Spectrometry

MDA-MB468 cells stably expressed Flag/HA-tagged CD73 were washed twice with PBS and lysed by NP-40 buffer (1% NP40, 10% glycerol, 25 mM Tris-HCl [pH 7.9] and protease inhibitor cocktails). CD73-interacting proteins were purified by immunopurification with Flag-M2 beads and washing four times with TBST buffer (137 mM NaC1, 20 mM Tris-HCl [pH 7.6], 0.1% Tween-20). The complex was eluted with 3×Flag peptide in TBS buffer. The eluted pellet was then separated on SDS-PAGE followed by silver staining as manufacture's protocol. The interest bands were cut

out for mass spectrum analysis (11).

Measurement of CD73 Activity

The supernatant of cell culture media was collected and centrifuged at 10,000 rpm for 5 min to remove insoluble particles. The concentration of adenosine in culture media was detected as the manufacture's protocol (Cell biolabs, San Diego, USA). Briefly, the supernatant was mixed with reaction mix in which way adenosine was converted into inosine by adenosine deaminase (ADA). Then inosine was converted into hypoxanthine by purine nucleoside phosphorylase (PNP). Finally, hypoxanthine was converted to xanthine and hydrogen peroxide by xanthine oxidase (XO). The resulting hydrogen peroxide was then detected with a highly specific fluorometric probe. Horseradish peroxidase catalyzes the reaction between the probe and hydrogen peroxide, which binds in a 1:1 ratio. Samples are compared to a known concentration of adenosine standard within the 96-well microtiter plate format. Samples and standards are incubated for 15 minutes and then read with a standard 96-well fluorometric plate reader.

Structural Modelling of CD73-OTUD4 and CD73-TRIM21 Complexes

We used MobiDB-lite (12) to identify the intrinsically disordered stretches in OTUD4 (UniProt ID: Q01804). Due the unavailability of experimentally resolved structures, we considered the structure predicted by AlphaFold2 (13) (AlphaFold DB ID: AF-Q01804-F1), which reported low confidence (pLDDT < 50) for large stretches of the protein; and we utilized RoseTTAFold (14), I-TASSER (15) and C-I-TASSER (16) to predict the optimal structural models for OTUD4. The conformations with the lowest error estimate of 7.68 ± 6.81 Å from RoseTTAFold and highest confidence score from C-I-TASSER were chosen (with c-score of 0.48). The conformation chosen from I-TASSER had a c-score of -2.47. The binding of OTUD4 to CD73 was examined by performing a series of guided docking simulations of these three conformers onto the experimentally resolved CD73 structure (PDB: 6TVG) (17) using the ClusPro (18) and Haddock

(19) servers in the presence of the constraints for interfacial regions deduced from transfection experiments described above. A total of 363 conformations for the OTUD4-CD73 complex were generated (275 by ClusPro and 88 by Haddock) for further analysis. Similarly, docking simulations of CD73 (PDB: 6TVG) onto TRIM21 (PDB: 2IWG) (20) yielded 130 conformations for the CD73-TRIM21 complex (90 from ClusPro and 40 from Haddock).

Druggability Simulations and Pharmacophore Modelling

Druggability simulations were performed on CD73 (PDB: 6TVG) in the presence of drug-like probe molecules -benzene, isobutane, imidazole, acetamide, isopropanol, isopropylamine, and acetate, using the MD simulation package NAMD (21) with the CHARMM force field (22) for proteins, along with the TIP3P water model (23), and the CGenFF force field (24) for the probes. Three independent runs of 40 ns were performed, as recommended. The trajectories were analyzed using the DruGUI (25) module of *ProDy* application programming interface (API) (26, 27). The reference structure (wt CD73 in PDB: 6TVG) was used to align all MD snapshots, employing C^{α} atoms and a cubic grid-based spatial representation with a grid edge size of 0.5 Å. We identified probe molecules as interacting with the protein when their non-hydrogen atoms were within a 4.0 Å distance from the protein atoms. We employed our Pharmmaker (28) framework to identify the residues engaged in high affinity interactions with individual probe types, concurrently identifying highly druggable spots for each probe. We ranked the interactions between residues and probes by their frequency of occurrence within and around the segment V275-D311 of CD73. We chose MD snapshots with the highest number of the most frequently occurring interaction pairs as templates for building the pharmacophore models (PMs). The PMs were used to screen the ZINC (29) and MolPort libraries of small molecules using Pharmit (30). Pharmit was also employed for extended PM construction, through iterative reutilization of the chemical groups belonging to hit molecules. The highest-ranking small molecules predicted by Pharmit were considered for further screening.

Elastic network model (ENM) Analysis of collective modes of motions

We assessed the impact of small molecule binding on the conformational dynamics of CD73 using the elastic network model (ENM) analyses implemented in *ProDy* API. First, we used the Gaussian Network Model (GNM) (31) to determine the soft (lowest frequency) modes of motion, also called global modes, accessible to CD73. The GNM provides a spectrum of *N*-1 normal modes for a protein of *N* residues. Each mode is characterized by a frequency and a mode shape (distribution of residue motions along the mode axis) described by the respective eigenvalues and eigenvectors of the network connectivity matrix. We used the default cutoff distance of 10 Å for defining the inter-residue contacts. The analysis of the three softest modes elucidated the residues involved in hinge sites that mediate these collective modes. Second, using the *ProDy* API, we carried out an Anisotropic Network Model (ANM) (32, 33) analysis with a default cutoff distance of 15 Å. We analyzed the cross-correlations driven by the softest three ANM modes between two sets of residues, residues P264, I266, R273 and K274 that coordinate the small molecule inhibitor Z22 and those (V275-D311) at the binding epitope of OTUD4, to quantify the allosteric effects potentially induced upon small molecule binding.

DSP (Digital Spatial Profiler) on Human Triple Negative Breast Cancers

TNBC tissue microarray (TMA) was baked at 60℃ for one hour, followed by deparaffinization, rehydration and digestion with proteinase K on the Leica BOND-RX Autostainer. Subsequently, the TMA underwent *in situ hybridization* with the GeoMx Whole Transcriptome Atlas (Human RNA probes for NGS (next generation sequence)) overnight at 37℃. After stringent washes with 2x SSC plus 50% formamide, the TMA was blocked with buffer R (Nanostring) and stained with morphology markers, including mouse-anti-PanCK-AF532, mouse-anti-CD73-CoraLite594, rabbit-anti-hu-OTUD4 plus anti-rabbit-IgG-AF647, and syto13 (nuclear). The stained the TMA slide was subsequently scanned on the *GeoMx-NGS DSP* instrument, and twenty-two ROIs

(Region of Interest) were selected from different cases based on the expression level of OTUD4 and CD73. The selected ROIs underwent auto-segmentation using PanCK staining positive (tumor segment) and PanCK staining negative (non-tumor segment). The oligo probes on tumor and non-tumor segments of the ROIs were separately cleaved by UV light and aspirated into individual plate wells with the collection tip. The collected probes were used to generate the next-generation sequence (NGS) library for RNA sequence following the manufacturer's instruction (GeoMx, Nanostring). Differentially expressed genes (DEGs) between OTUD4 (or CD73) high expression with OTUD4 (or CD73) low expression in tumor compartment and non-tumor compartment were subject to gene pathway analysis (KEGG/Reactome) and Gene Set Enrichment Analysis (GSEA).

T Cell Suppression Assay

Human breast cancer cells MDA-MB231, MDA-MB468, as well as their corresponding OTUD4 or CD73 knock-in and knock-down cells, were treated with100 μM APCP for 24 hours. They were washed with fresh culture media three times. Human PBMCs stimulated with anti-CD3 (30 ng/ml; Miltenyi Biotech) were co-cultured with tumor cells at the ratios of 1:10 (PBMCs: tumor cells) for additional three days, following the procedure described previously (10). PBMCs were collected for the staining with the markers associated with the activation, proliferation, and cytokine secretion, and were analyzed using flow cytometry.

REFERENCES

1. Mertins P, Mani DR, Ruggles KV, Gillette MA, Clauser KR, Wang P, et al. Proteogenomics connects somatic mutations to signalling in breast cancer. *Nature.* 2016;534(7605):55-62.
2. Gruosso T, Gigoux M, Manem VSK, Bertos N, Zuo D, Perlitch I, et al. Spatially distinct tumor immune microenvironments stratify triple-negative breast cancers. *J Clin Invest.* 2019;129(4):1785-800.
3. Lin A, Qi C, Wei T, Li M, Cheng Q, Liu Z, et al. CAMOIP: a web server for comprehensive analysis on multi-omics of immunotherapy in pan-cancer. *Brief Bioinform.* 2022;23(3).
4. Li T, Fu J, Zeng Z, Cohen D, Li J, Chen Q, et al. TIMER2.0 for analysis of tumor-infiltrating immune cells. *Nucleic Acids Res.* 2020;48(W1):W509-W14.
5. Jiang P, Gu S, Pan D, Fu J, Sahu A, Hu X, et al. Signatures of T cell dysfunction and exclusion predict cancer immunotherapy response. *Nat Med.* 2018;24(10):1550-8.
6. Liberzon A, Birger C, Thorvaldsdottir H, Ghandi M, Mesirov JP, and Tamayo P. The Molecular Signatures Database (MSigDB) hallmark gene set collection. *Cell Syst.* 2015;1(6):417-25.
7. Kanehisa M, Furumichi M, Tanabe M, Sato Y, and Morishima K. KEGG: new perspectives on genomes, pathways, diseases and drugs. *Nucleic Acids Res.* 2017;45(D1):D353-D61.
8. The Gene Ontology C. Expansion of the Gene Ontology knowledgebase and resources. *Nucleic Acids Res.* 2017;45(D1):D331-D8.
9. Fabregat A, Jupe S, Matthews L, Sidiropoulos K, Gillespie M, Garapati P, et al. The Reactome Pathway Knowledgebase. *Nucleic Acids Res.* 2018;46(D1):D649-D55.
10. Fu Z, Chen S, Zhu Y, Zhang D, Xie P, Jiao Q, et al. Proteolytic regulation of CD73 by TRIM21 orchestrates tumor immunogenicity. *Sci Adv.* 2023;9(1):eadd6626.
11. Hu D, Gur M, Zhou Z, Gamper A, Hung MC, Fujita N, et al. Interplay between arginine methylation and ubiquitylation regulates KLF4-mediated genome stability and carcinogenesis. *Nat Commun.* 2015;6:8419.

12. Piovesan D, Necci M, Escobedo N, Monzon AM, Hatos A, Mičetić I, et al. MobiDB: intrinsically disordered proteins in 2021. *Nucleic Acids Res.* 2021;49(D1):D361-d7.

13. Jumper J, and Hassabis D. Protein structure predictions to atomic accuracy with AlphaFold. *Nat Methods.* 2022;19(1):11-2.

14. Baek M, DiMaio F, Anishchenko I, Dauparas J, Ovchinnikov S, Lee GR, et al. Accurate prediction of protein structures and interactions using a three-track neural network. *Science.* 2021;373(6557):871-6.

15. Yang J, Yan R, Roy A, Xu D, Poisson J, and Zhang Y. The I-TASSER Suite: protein structure and function prediction. *Nat Methods.* 2015;12(1):7-8.

16. Zheng W, Zhang C, Li Y, Pearce R, Bell EW, and Zhang Y. Folding non-homologous proteins by coupling deep-learning contact maps with I-TASSER assembly simulations. *Cell Rep Methods.* 2021;1(3).

17. Bhattarai S, Pippel J, Scaletti E, Idris R, Freundlieb M, Rolshoven G, et al. 2-Substituted alpha,beta-Methylene-ADP Derivatives: Potent Competitive Ecto-5'-nucleotidase (CD73) Inhibitors with Variable Binding Modes. *J Med Chem.* 2020;63(6):2941-57.

18. Kozakov D, Hall DR, Xia B, Porter KA, Padhorny D, Yueh C, et al. The ClusPro web server for protein-protein docking. *Nat Protoc.* 2017;12(2):255-78.

19. van Zundert GCP, Rodrigues J, Trellet M, Schmitz C, Kastritis PL, Karaca E, et al. The HADDOCK2.2 Web Server: User-Friendly Integrative Modeling of Biomolecular Complexes. *J Mol Biol.* 2016;428(4):720-5.

20. James LC, Keeble AH, Khan Z, Rhodes DA, and Trowsdale J. Structural basis for PRYSPRY-mediated tripartite motif (TRIM) protein function. *Proc Natl Acad Sci U S A.* 2007;104(15):6200-5.

21. Phillips JC, Hardy DJ, Maia JDC, Stone JE, Ribeiro JV, Bernardi RC, et al. Scalable molecular dynamics on CPU and GPU architectures with NAMD. *J Chem Phys.* 2020;153(4):044130.

22. Best RB, Zhu X, Shim J, Lopes PE, Mittal J, Feig M, et al. Optimization of the additive CHARMM all-atom protein force field targeting improved sampling of the backbone φ, ψ and side-chain χ(1) and χ(2) dihedral angles. *J Chem Theory Comput.* 2012;8(9):3257-73.

23. Nayar D, Agarwal M, and Chakravarty C. Comparison of Tetrahedral Order, Liquid State Anomalies, and Hydration Behavior of mTIP3P and TIP4P Water Models. *J Chem Theory Comput.* 2011;7(10):3354-67.

24. Vanommeslaeghe K, and MacKerell AD, Jr. CHARMM additive and polarizable force fields for biophysics and computer-aided drug design. *Biochim Biophys Acta.* 2015;1850(5):861-71.

25. Bakan A, Nevins N, Lakdawala AS, and Bahar I. Druggability Assessment of Allosteric Proteins by Dynamics Simulations in the Presence of Probe Molecules. *J Chem Theory Comput.* 2012;8(7):2435-47.

26. Bakan A, Meireles LM, and Bahar I. ProDy: protein dynamics inferred from theory and experiments. *Bioinformatics.* 2011;27(11):1575-7.

27. Zhang S, Krieger JM, Zhang Y, Kaya C, Kaynak B, Mikulska-Ruminska K, et al. ProDy 2.0: increased scale and scope after 10 years of protein dynamics modelling with Python. *Bioinformatics.* 2021;37(20):3657-9.

28. Lee JY, Krieger JM, Li H, and Bahar I. Pharmmaker: Pharmacophore modeling and hit identification based on druggability simulations. *Protein Sci.* 2020;29(1):76-86.

29. Sterling T, and Irwin JJ. ZINC 15--Ligand Discovery for Everyone. *J Chem Inf Model.* 2015;55(11):2324-37.

30. Sunseri J, and Koes DR. Pharmit: interactive exploration of chemical space. *Nucleic Acids Res.* 2016;44(W1):W442-8.

31. Bahar I, Atilgan AR, and Erman B. Direct evaluation of thermal fluctuations in proteins using a single-parameter harmonic potential. *Fold Des.* 1997;2(3):173-81.

32. Atilgan AR, Durell SR, Jernigan RL, Demirel MC, Keskin O, and Bahar I. Anisotropy of

fluctuation dynamics of proteins with an elastic network model. *Biophys J.* 2001;80(1):505-15.

33. Doruker P, Atilgan AR, and Bahar I. Dynamics of proteins predicted by molecular dynamics simulations and analytical approaches: application to alpha-amylase inhibitor. *Proteins.* 2000;40(3):512-24.

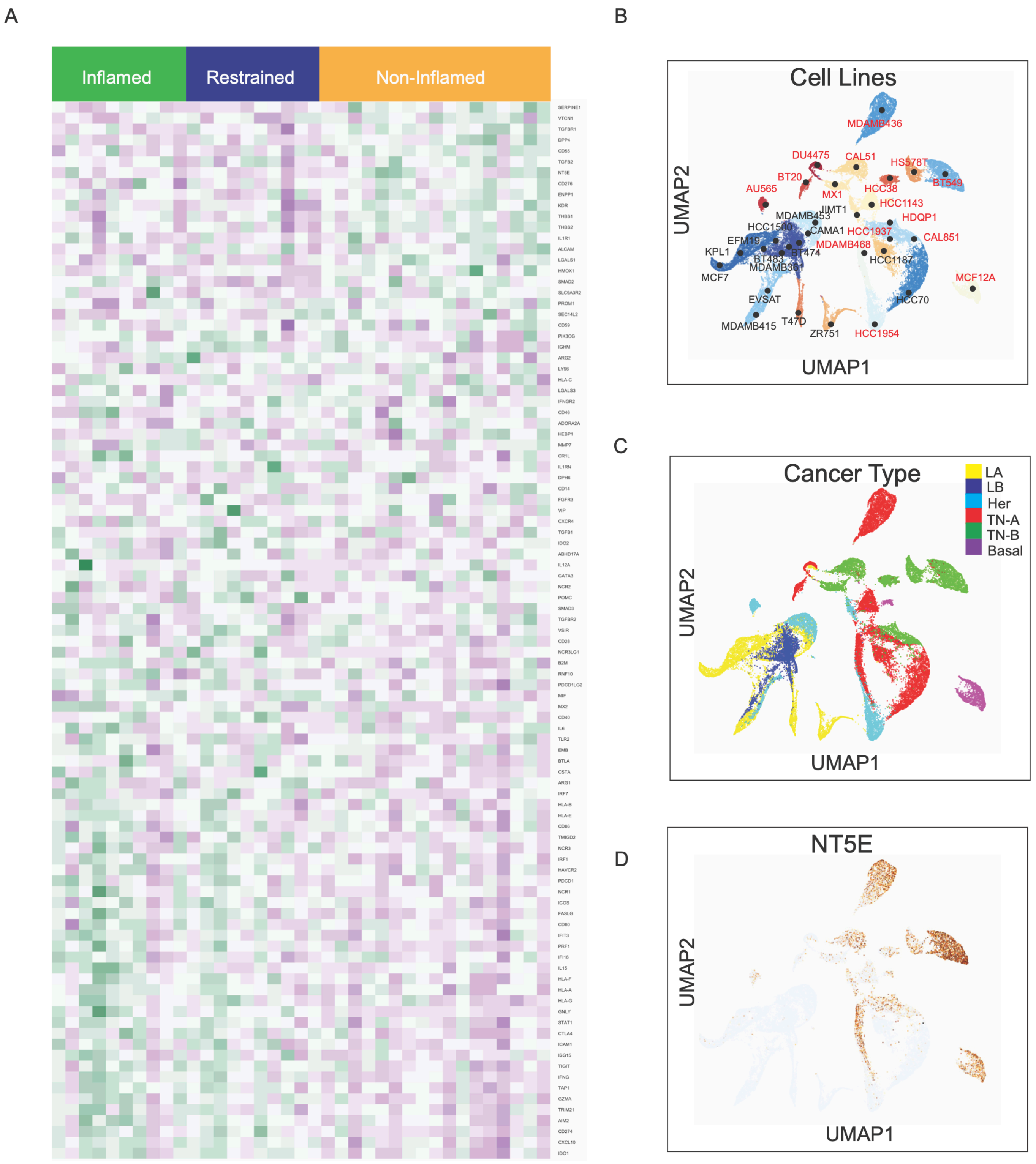
A
Inflamed
Restrained
Non-Inflamed
SERPINE1
VTCN1
TGFBR1
DPP4
CD55
TGFB2
NT5E
CD276
ENPP1
KDR
THBS1
THBS2
IL1R1
ALCAM
LGALS1
HMOX1
SMAD2
SLC9A3R2
PROM1
SEC14L2
CD59
PIK3CG
IGHM
ARG2
LY96
HLA-C
LGALS3
IFNGR2
CD46
ADORA2A
HEBP1
MMP7
CR1L
IL1RN
DPH6
CD14
FGFR3
VIP
CXCR4
TGFB1
IDO2
ABHD17A
IL12A
GATA3
NCR2
POMC
SMAD3
TGFBR2
VSIR
CD28
NCR3LG1
B2M
RNF10
PDCD1LG2
MIF
MX2
CD40
IL6
TLR2
EMB
BTLA
CSTA
ARG1
IRF7
HLA-B
HLA-E
CD86
TMIGD2
NCR3
IRF1
HAVCR2
PDCD1
NCR1
ICOS
FASLG
CD80
IFIT3
PRF1
IFI16
IL15
HLA-F
HLA-A
HLA-G
GNLY
STAT1
CTLA4
ICAM1
ISG15
TIGIT
IFNG
TAP1
GZMA
TRIM21
AIM2
CD274
CXCL10
IDO1
B
Cell Lines
UMAP2
UMAP1
MDAMB436
DU4475
CAL51
HS578T
BT549
BT20
AU565
MX1
HCC38
JIMT1
HCC1143
MDAMB453
HDQP1
HCC1500
CAMA1
HCC1937
CAL851
EFM19
MDAMB468
KPL1
BT474
BT483
HCC1187
MDAMB361
MCF7
MCF12A
EVSAT
HCC70
T47D
MDAMB415
ZR751
HCC1954
C
Cancer Type
LA
LB
Her
TN-A
TN-B
Basal
UMAP2
UMAP1
D
NT5E
UMAP2
UMAP1

Figure S1

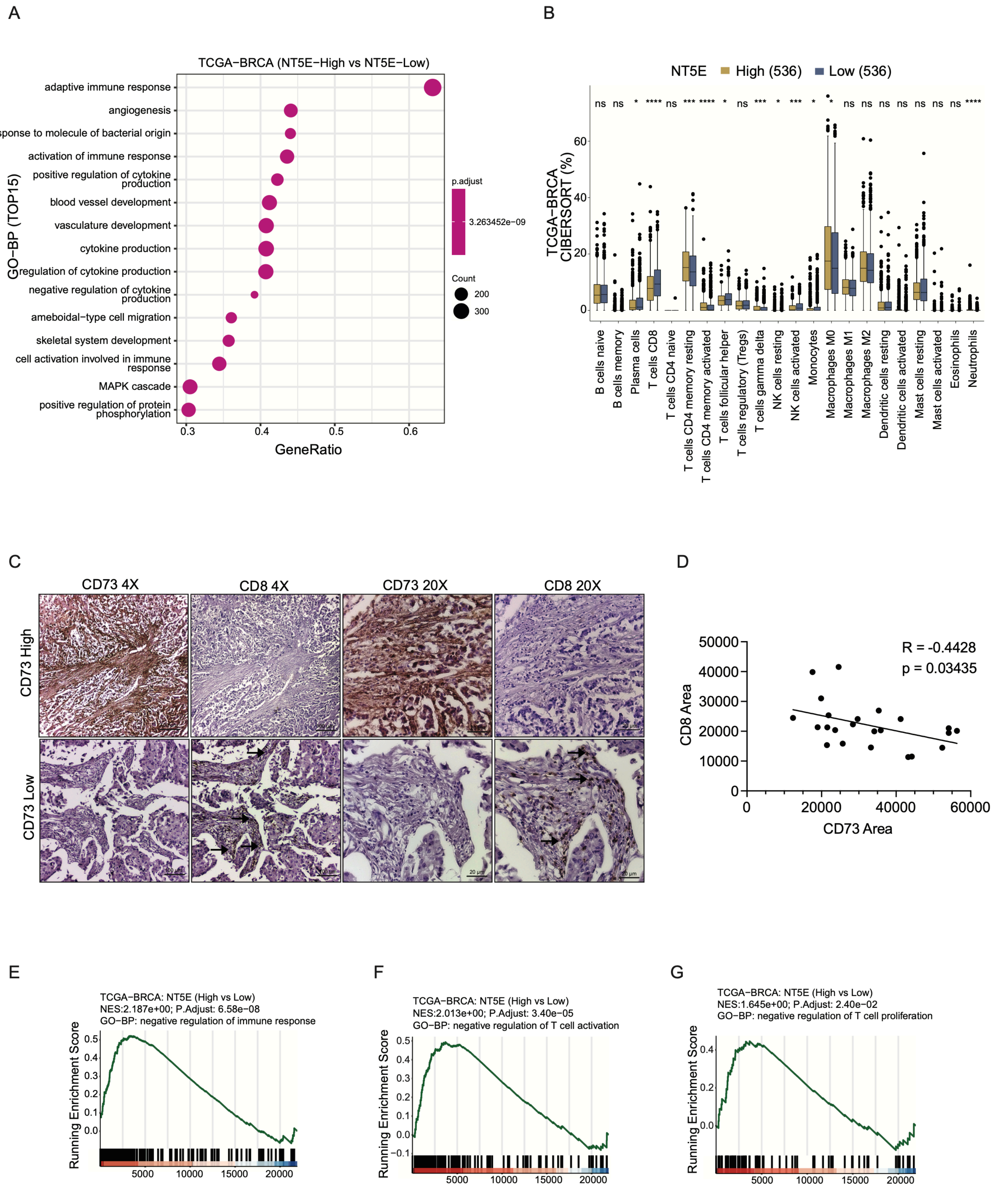
A
TCGA−BRCA (NT5E−High vs NT5E−Low)
adaptive immune response
angiogenesis
response to molecule of bacterial origin
activation of immune response
positive regulation of cytokine production
blood vessel development
vasculature development
cytokine production
regulation of cytokine production
negative regulation of cytokine production
ameboidal−type cell migration
skeletal system development
cell activation involved in immune response
MAPK cascade
positive regulation of protein phosphorylation
GO−BP (TOP15)
GeneRatio
p.adjust
3.263452e−09
Count
200
300
B
NT5E
High (536)
Low (536)
TCGA-BRCA CIBERSORT (%)
ns ns * **** ns *** **** * ns *** * *** * * ns ns ns ns ns ns ns ****
B cells naive
B cells memory
Plasma cells
T cells CD8
T cells CD4 naive
T cells CD4 memory resting
T cells CD4 memory activated
T cells follicular helper
T cells regulatory (Tregs)
T cells gamma delta
NK cells resting
NK cells activated
Monocytes
Macrophages M0
Macrophages M1
Macrophages M2
Dendritic cells resting
Dendritic cells activated
Mast cells resting
Mast cells activated
Eosinophils
Neutrophils
C
CD73 4X
CD8 4X
CD73 20X
CD8 20X
CD73 High
CD73 Low
D
R = -0.4428
p = 0.03435
CD8 Area
CD73 Area
E
TCGA−BRCA: NT5E (High vs Low)
NES:2.187e+00; P.Adjust: 6.58e−08
GO−BP: negative regulation of immune response
Running Enrichment Score
F
TCGA−BRCA: NT5E (High vs Low)
NES:2.013e+00; P.Adjust: 3.40e−05
GO−BP: negative regulation of T cell activation
Running Enrichment Score
G
TCGA−BRCA: NT5E (High vs Low)
NES:1.645e+00; P.Adjust: 2.40e−02
GO−BP: negative regulation of T cell proliferation
Running Enrichment Score

Figure S2

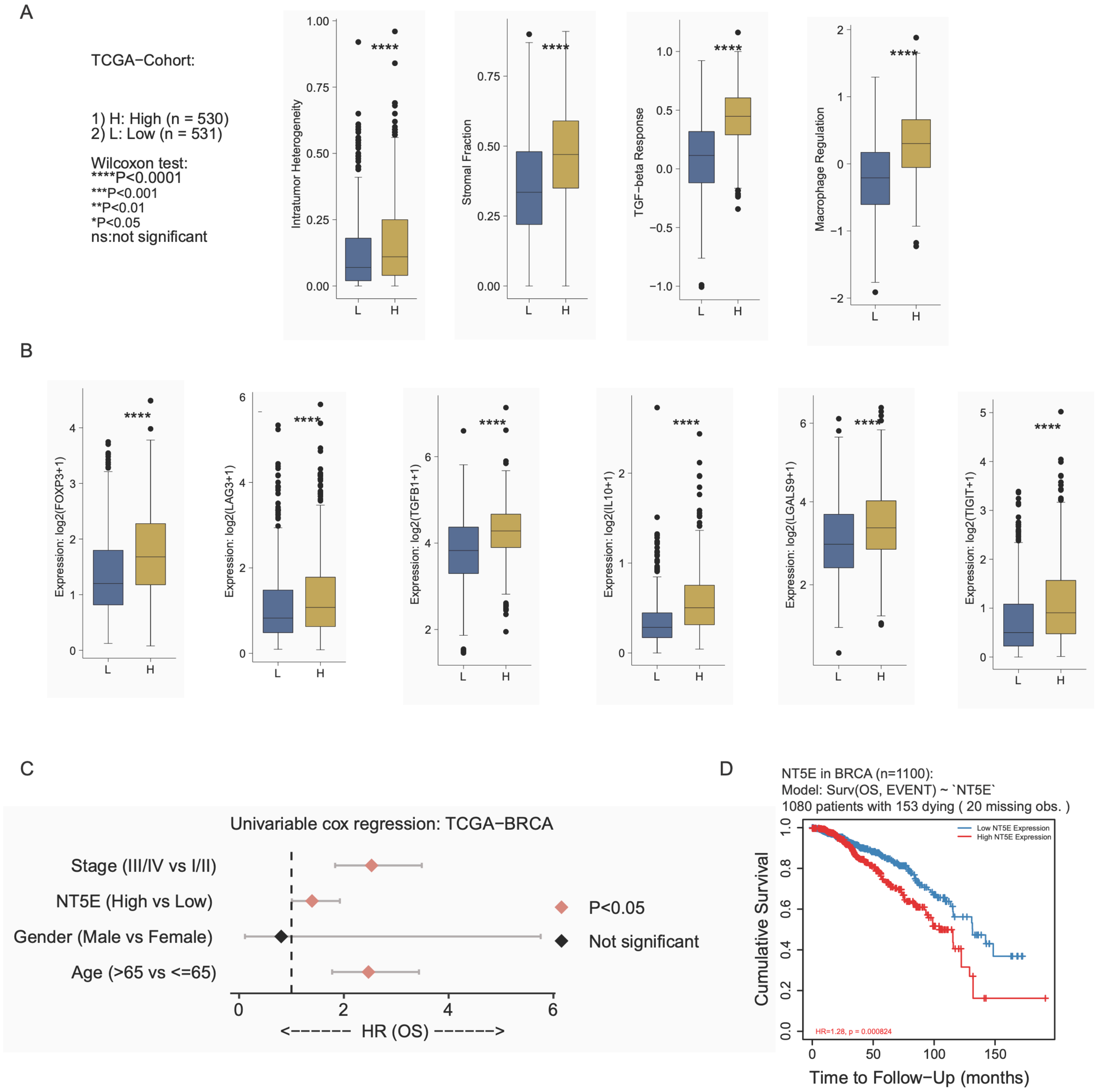
A
TCGA−Cohort:
1) H: High (n = 530)
2) L: Low (n = 531)
Wilcoxon test:
****P<0.0001
***P<0.001
**P<0.01
*P<0.05
ns:not significant
Intratumor Heterogeneity
Stromal Fraction
TGF−beta Response
Macrophage Regulation
B
Expression: log2(FOXP3+1)
Expression: log2(LAG3+1)
Expression: log2(TGFB1+1)
Expression: log2(IL10+1)
Expression: log2(LGALS9+1)
Expression: log2(TIGIT+1)
C
Univariable cox regression: TCGA−BRCA
Stage (III/IV vs I/II)
NT5E (High vs Low)
Gender (Male vs Female)
Age (>65 vs <=65)
P<0.05
Not significant
<------ HR (OS) ------>
D
NT5E in BRCA (n=1100):
Model: Surv(OS, EVENT) ~ `NT5E`
1080 patients with 153 dying (20 missing obs.)
Low NT5E Expression
High NT5E Expression
HR=1.28, p = 0.000824
Cumulative Survival
Time to Follow−Up (months)

Figure S3

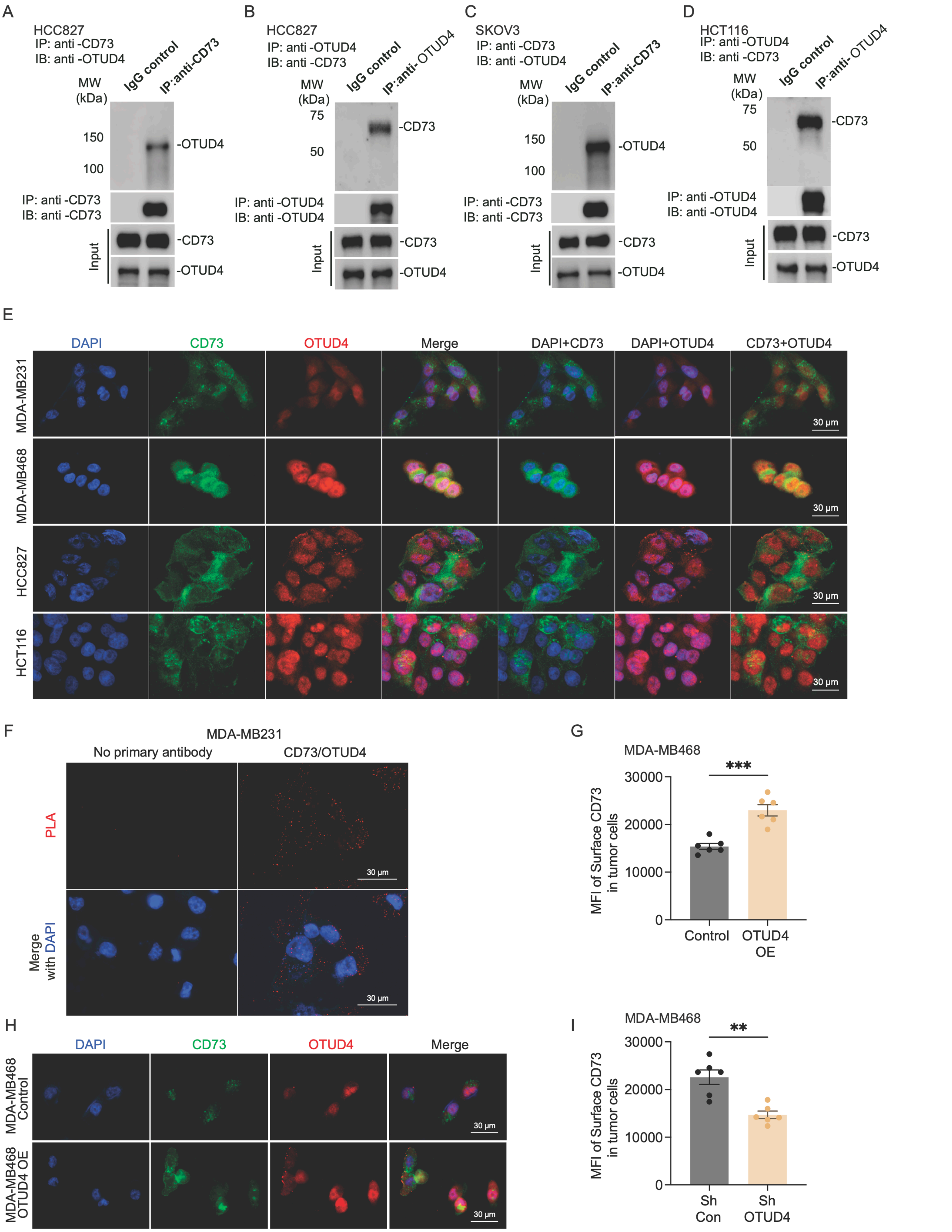
A
HCC827
IP: anti -CD73
IB: anti -OTUD4
MW (kDa)
IgG control
IP:anti-CD73
150
100
-OTUD4
IP: anti -CD73
IB: anti -CD73
Input
-CD73
-OTUD4
B
HCC827
IP: anti -OTUD4
IB: anti -CD73
MW (kDa)
IgG control
IP:anti-OTUD4
75
50
-CD73
IP: anti -OTUD4
IB: anti -OTUD4
Input
-CD73
-OTUD4
C
SKOV3
IP: anti -CD73
IB: anti -OTUD4
MW (kDa)
IgG control
IP:anti-CD73
150
100
-OTUD4
IP: anti -CD73
IB: anti -CD73
Input
-CD73
-OTUD4
D
HCT116
IP: anti -OTUD4
IB: anti -CD73
MW (kDa)
IgG control
IP:anti-OTUD4
75
50
-CD73
IP: anti -OTUD4
IB: anti -OTUD4
Input
-CD73
-OTUD4
E
DAPI
CD73
OTUD4
Merge
DAPI+CD73
DAPI+OTUD4
CD73+OTUD4
MDA-MB231
MDA-MB468
HCC827
HCT116
30 µm
F
MDA-MB231
No primary antibody
CD73/OTUD4
PLA
Merge with DAPI
30 µm
G
MDA-MB468
MFI of Surface CD73 in tumor cells
30000
20000
10000
0
***
Control
OTUD4 OE
H
DAPI
CD73
OTUD4
Merge
MDA-MB468 Control
MDA-MB468 OTUD4 OE
30 µm
I
MDA-MB468
MFI of Surface CD73 in tumor cells
30000
20000
10000
0
**
Sh Con
Sh OTUD4

Figure S4

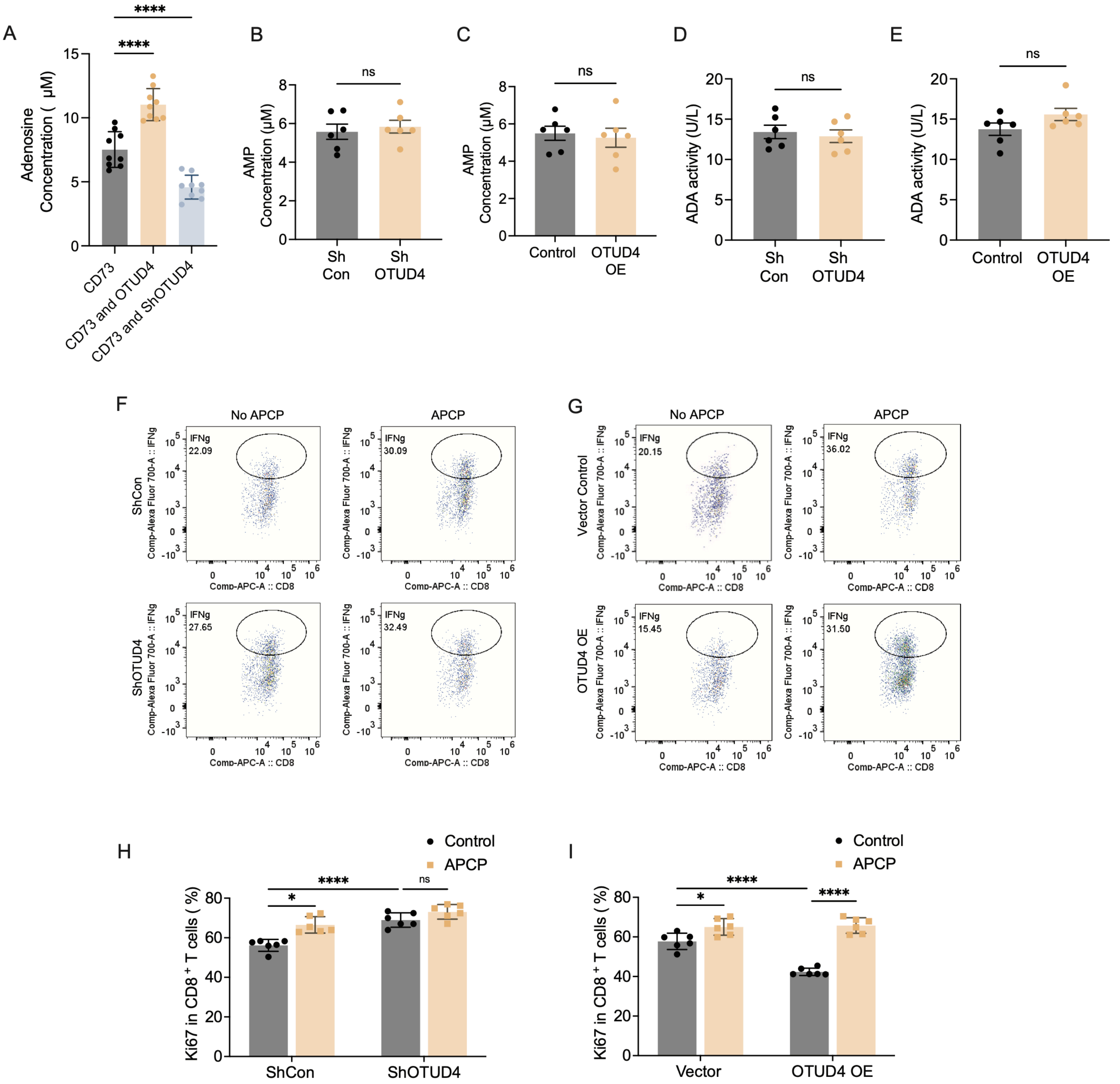
A
B
C
D
E
Adenosine Concentration (μM)
AMP Concentration (μM)
ADA activity (U/L)
CD73
CD73 and OTUD4
CD73 and ShOTUD4
Sh Con
Sh OTUD4
Control
OTUD4 OE
ns
****
F
G
No APCP
APCP
ShCon
ShOTUD4
Vector Control
OTUD4 OE
IFNg
22.09
30.09
27.65
32.49
20.15
36.02
15.45
31.50
Comp-Alexa Fluor 700-A :: IFNg
Comp-APC-A :: CD8
H
I
Control
APCP
Ki67 in CD8 + T cells (%)
ShCon
ShOTUD4
Vector
OTUD4 OE
*
****
ns

Figure S5

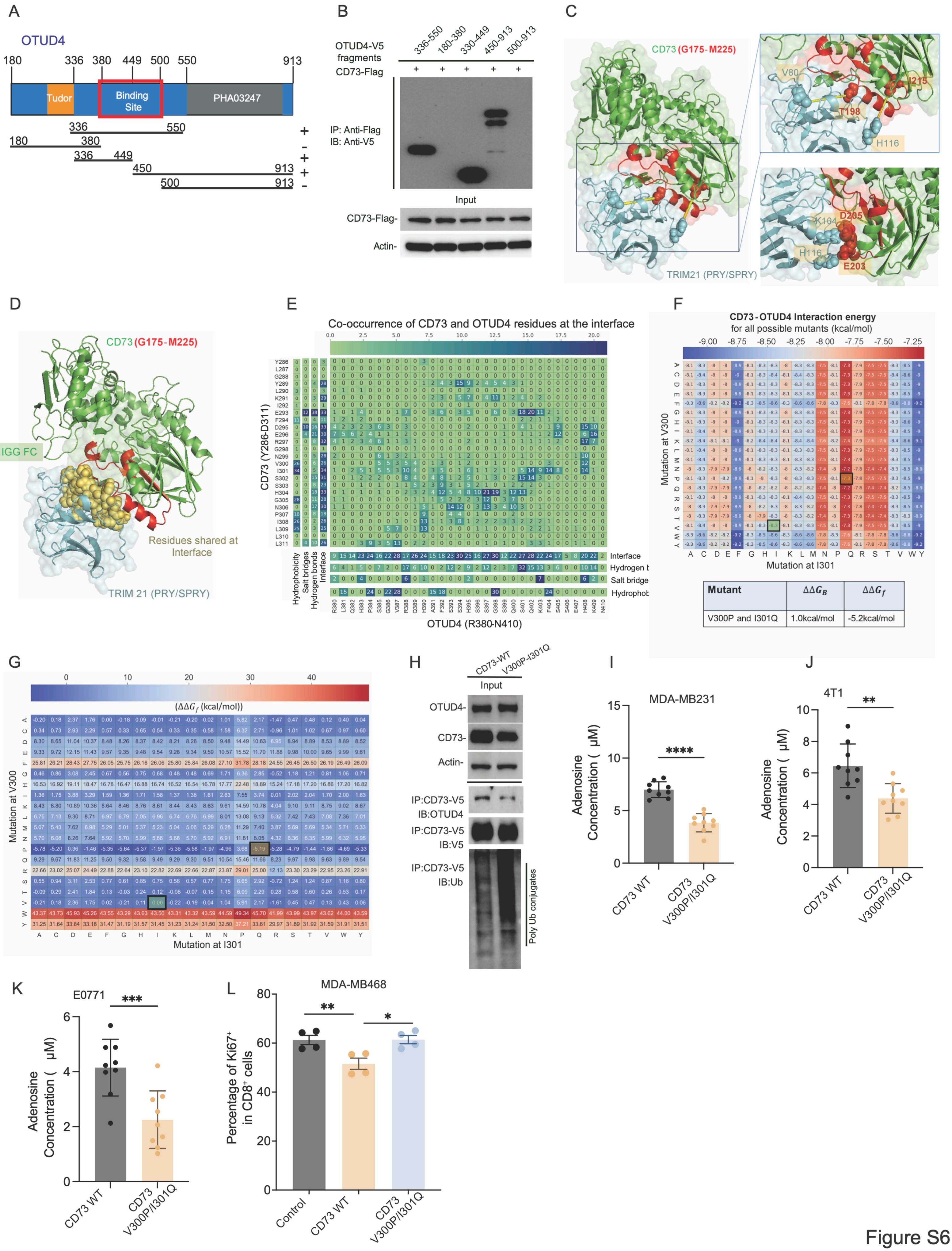

Mutant	$\Delta\Delta G_B$	$\Delta\Delta G_f$
V300P and I301Q	1.0kcal/mol	-5.2kcal/mol

Figure S6

A

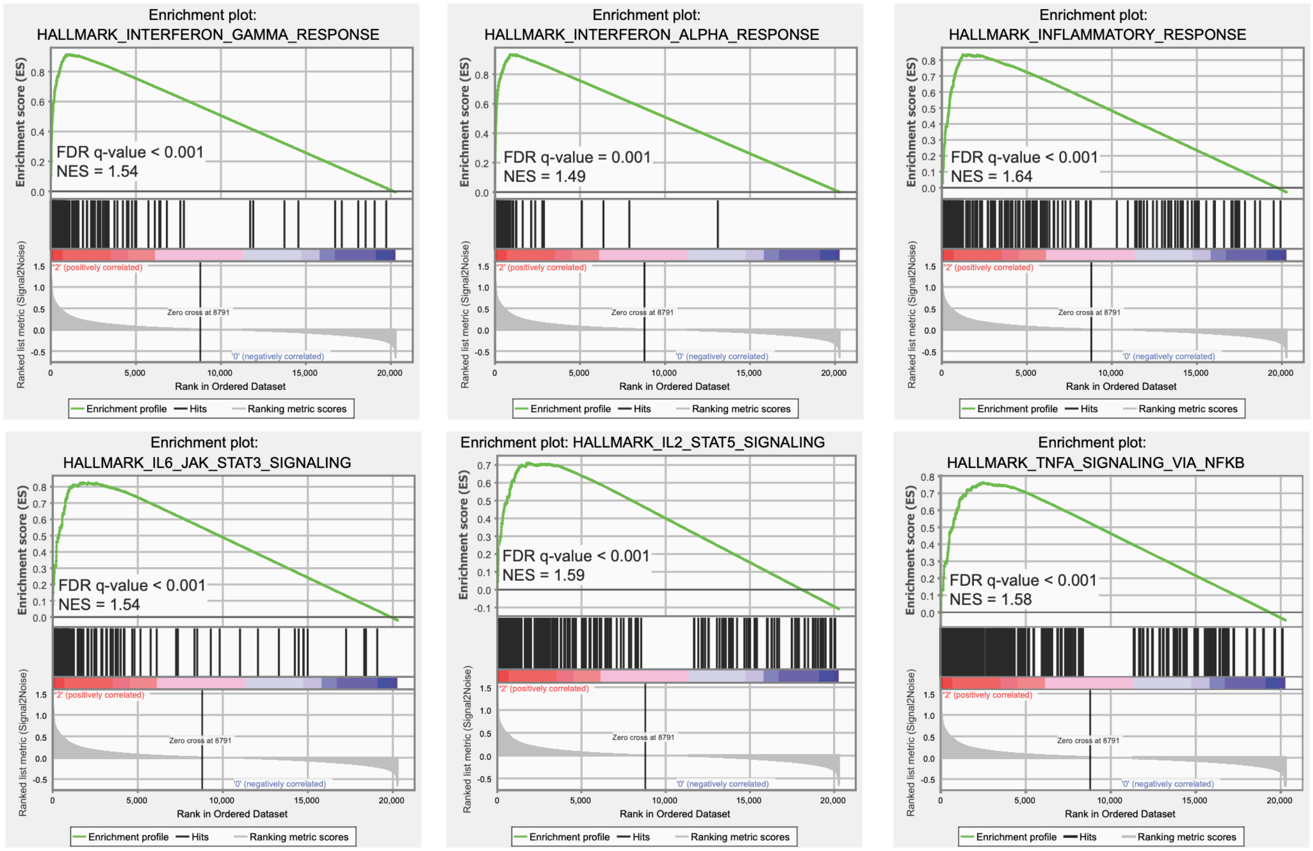
Enrichment plot:
HALLMARK_INTERFERON_GAMMA_RESPONSE
FDR q-value < 0.001
NES = 1.54
Enrichment plot:
HALLMARK_INTERFERON_ALPHA_RESPONSE
FDR q-value = 0.001
NES = 1.49
Enrichment plot:
HALLMARK_INFLAMMATORY_RESPONSE
FDR q-value < 0.001
NES = 1.64
Enrichment plot:
HALLMARK_IL6_JAK_STAT3_SIGNALING
FDR q-value < 0.001
NES = 1.54
Enrichment plot: HALLMARK_IL2_STAT5_SIGNALING
FDR q-value < 0.001
NES = 1.59
Enrichment plot:
HALLMARK_TNFA_SIGNALING_VIA_NFKB
FDR q-value < 0.001
NES = 1.58
Enrichment score (ES)
Ranked list metric (Signal2Noise)
'2' (positively correlated)
Zero cross at 8791
'0' (negatively correlated)
Rank in Ordered Dataset
Enrichment profile
Hits
Ranking metric scores

Figure S7

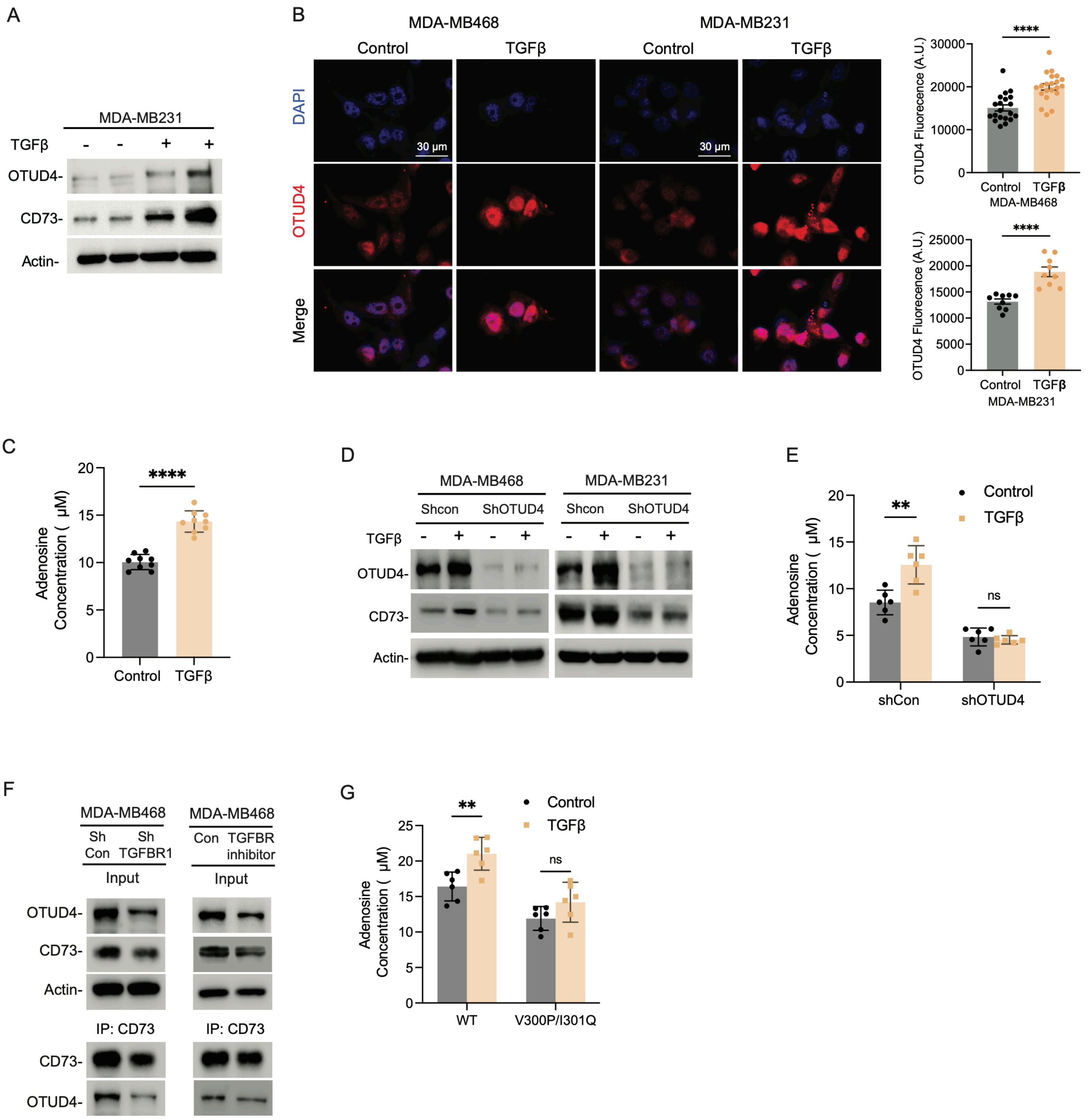

A
MDA-MB231
TGFβ - - + +
OTUD4-
CD73-
Actin-
B
MDA-MB468
MDA-MB231
Control
TGFβ
Control
TGFβ
DAPI
OTUD4
Merge
30 µm
30 µm
****
OTUD4 Fluorecence (A.U.)
30000
20000
10000
0
Control
TGFβ
MDA-MB468
****
OTUD4 Fluorecence (A.U.)
25000
20000
15000
10000
5000
0
Control
TGFβ
MDA-MB231
C
Adenosine Concentration (µM)
20
15
10
5
0
****
Control
TGFβ
D
MDA-MB468
MDA-MB231
Shcon
ShOTUD4
Shcon
ShOTUD4
TGFβ - + - + - + - +
OTUD4-
CD73-
Actin-
E
Adenosine Concentration (µM)
20
15
10
5
0
**
ns
Control
TGFβ
shCon
shOTUD4
F
MDA-MB468
Sh Con
Sh TGFBR1
Input
MDA-MB468
Con
TGFBR inhibitor
Input
OTUD4-
CD73-
Actin-
IP: CD73
IP: CD73
CD73-
OTUD4-
G
Adenosine Concentration (µM)
25
20
15
10
5
0
**
ns
Control
TGFβ
WT
V300P/I301Q

Figure S8

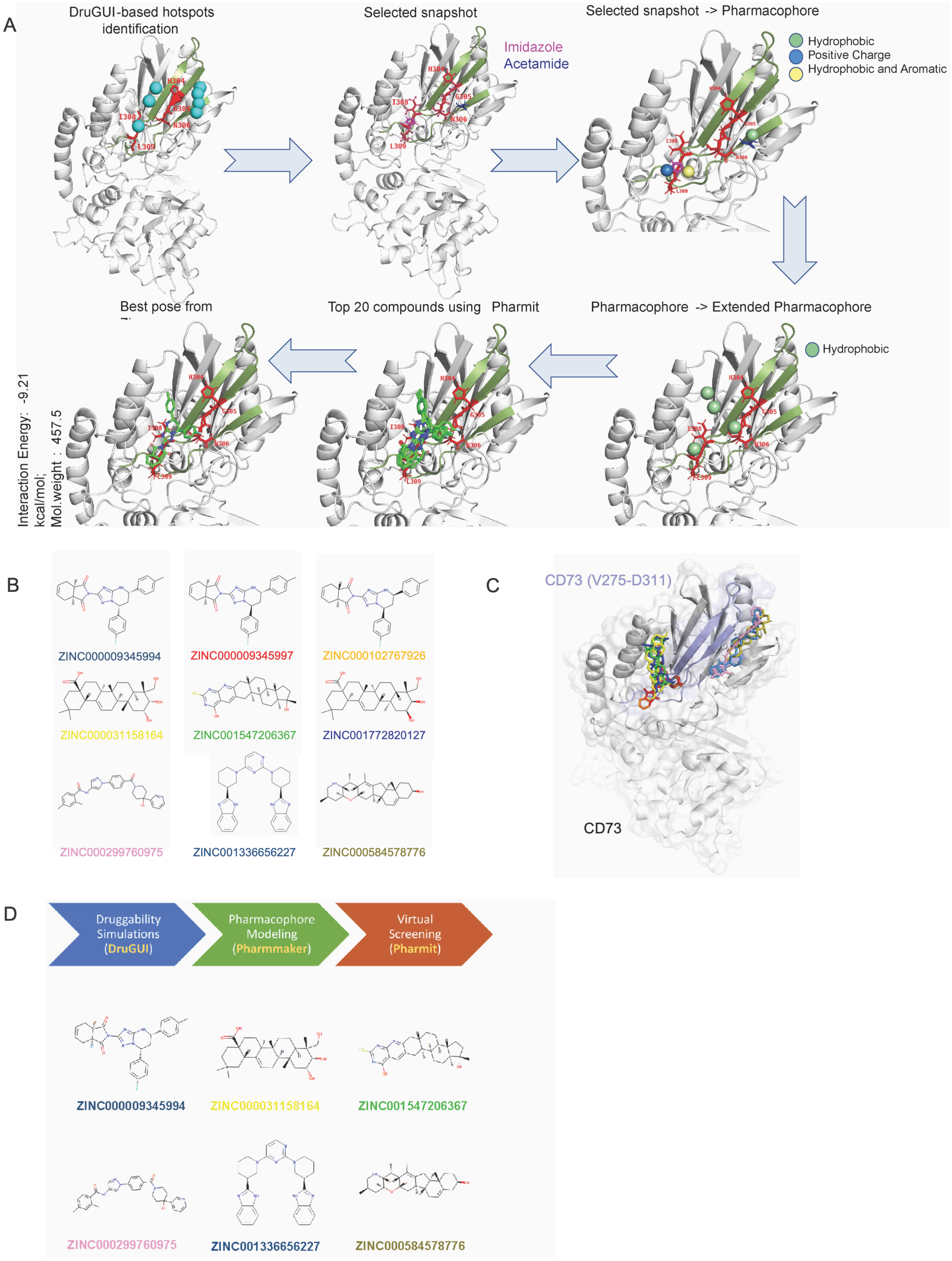
A
DruGUI-based hotspots identification
Selected snapshot
Imidazole
Acetamide
Selected snapshot -> Pharmacophore
Hydrophobic
Positive Charge
Hydrophobic and Aromatic
Pharmacophore -> Extended Pharmacophore
Hydrophobic
Top 20 compounds using Pharmit
Best pose from
Interaction Energy: -9.21 kcal/mol;
Mol.weight : 457.5
B
ZINC000009345994
ZINC000009345997
ZINC000102767926
ZINC000031158164
ZINC001547206367
ZINC001772820127
ZINC000299760975
ZINC001336656227
ZINC000584578776
C
CD73 (V275-D311)
CD73
D
Druggability Simulations (DruGUI)
Pharmacophore Modeling (Pharmmaker)
Virtual Screening (Pharmit)
ZINC000009345994
ZINC000031158164
ZINC001547206367
ZINC000299760975
ZINC001336656227
ZINC000584578776

Figure S9

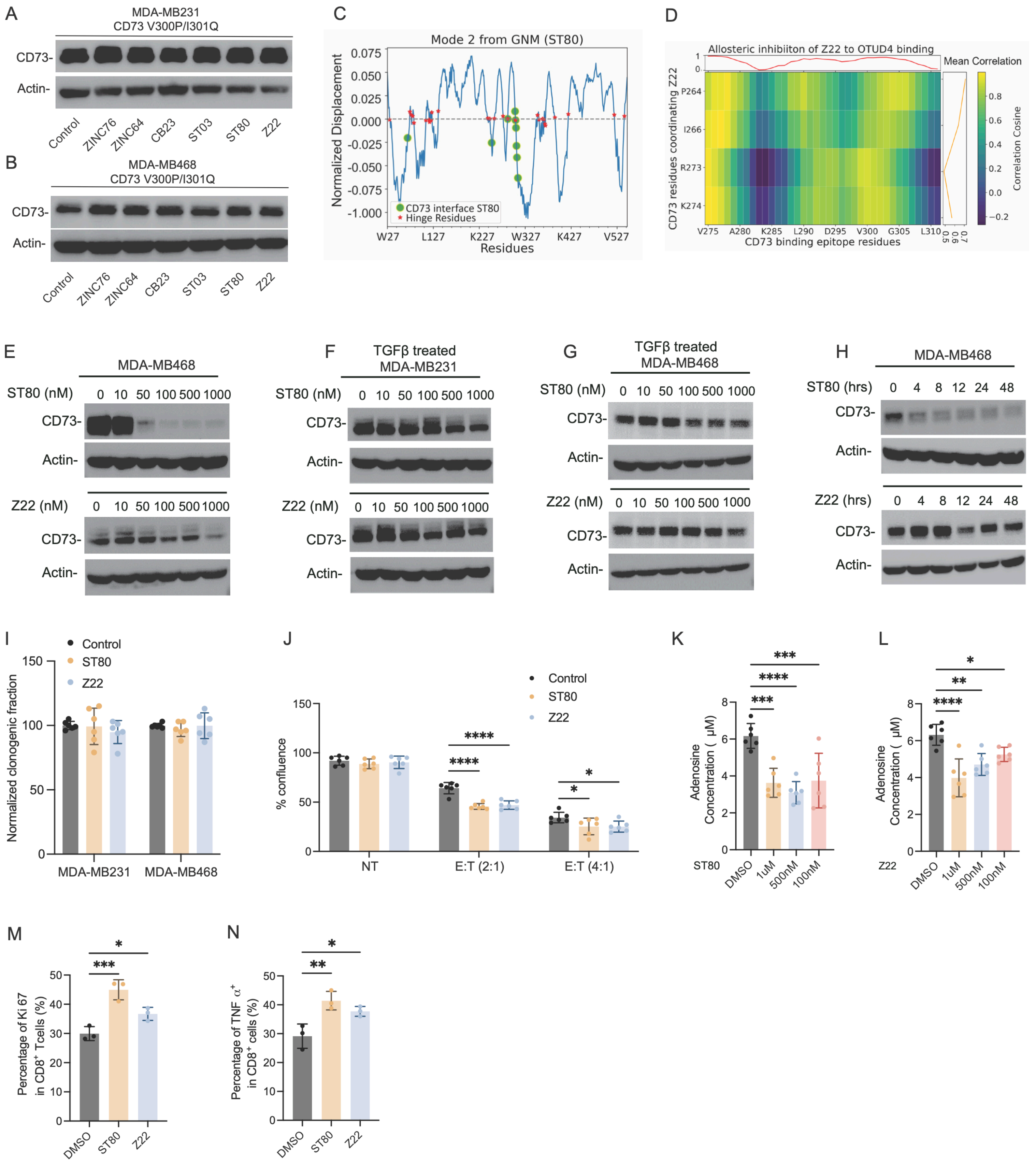
A
MDA-MB231
CD73 V300P/I301Q
CD73-
Actin-
Control
ZINC76
ZINC64
CB23
ST03
ST80
Z22
B
MDA-MB468
CD73 V300P/I301Q
C
Mode 2 from GNM (ST80)
Normalized Displacement
Residues
CD73 interface ST80
Hinge Residues
D
Allosteric inhibiiton of Z22 to OTUD4 binding
Mean Correlation
CD73 residues coordinating Z22
CD73 binding epitope residues
Correlation Cosine
E
MDA-MB468
ST80 (nM)
Z22 (nM)
F
TGFβ treated MDA-MB231
G
TGFβ treated MDA-MB468
H
MDA-MB468
ST80 (hrs)
Z22 (hrs)
I
Normalized clonogenic fraction
Control
ST80
Z22
MDA-MB231
MDA-MB468
J
% confluence
NT
E:T (2:1)
E:T (4:1)
K
Adenosine Concentration (µM)
ST80
DMSO
1uM
500nM
100nM
L
Z22
M
Percentage of Ki 67 in CD8+ Tcells (%)
DMSO
ST80
Z22
N
Percentage of TNF α+ in CD8+ cells (%)

Figure S10

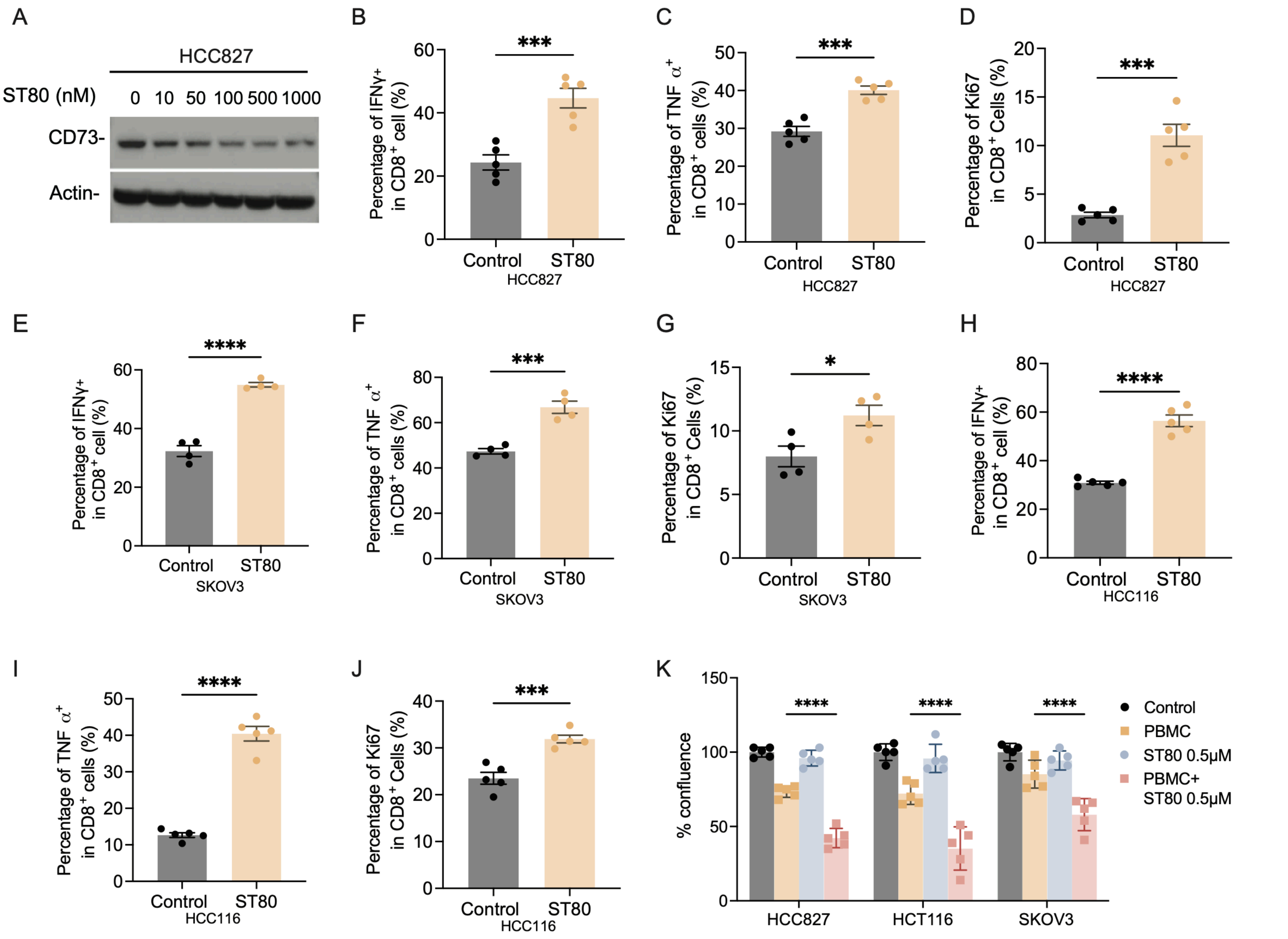
A
HCC827
ST80 (nM)
0 10 50 100 500 1000
CD73-
Actin-
B
Percentage of IFNγ+ in CD8+ cell (%)
***
Control
ST80
HCC827
C
Percentage of TNF α+ in CD8+ cells (%)
***
Control
ST80
HCC827
D
Percentage of Ki67 in CD8+ Cells (%)
***
Control
ST80
HCC827
E
Percentage of IFNγ+ in CD8+ cell (%)
****
Control
ST80
SKOV3
F
Percentage of TNF α+ in CD8+ cells (%)
***
Control
ST80
SKOV3
G
Percentage of Ki67 in CD8+ Cells (%)
*
Control
ST80
SKOV3
H
Percentage of IFNγ+ in CD8+ cell (%)
****
Control
ST80
HCC116
I
Percentage of TNF α+ in CD8+ cells (%)
****
Control
ST80
HCC116
J
Percentage of Ki67 in CD8+ Cells (%)
***
Control
ST80
HCC116
K
% confluence
****
HCC827
HCT116
SKOV3
Control
PBMC
ST80 0.5μM
PBMC+ ST80 0.5μM

Figure S11

A

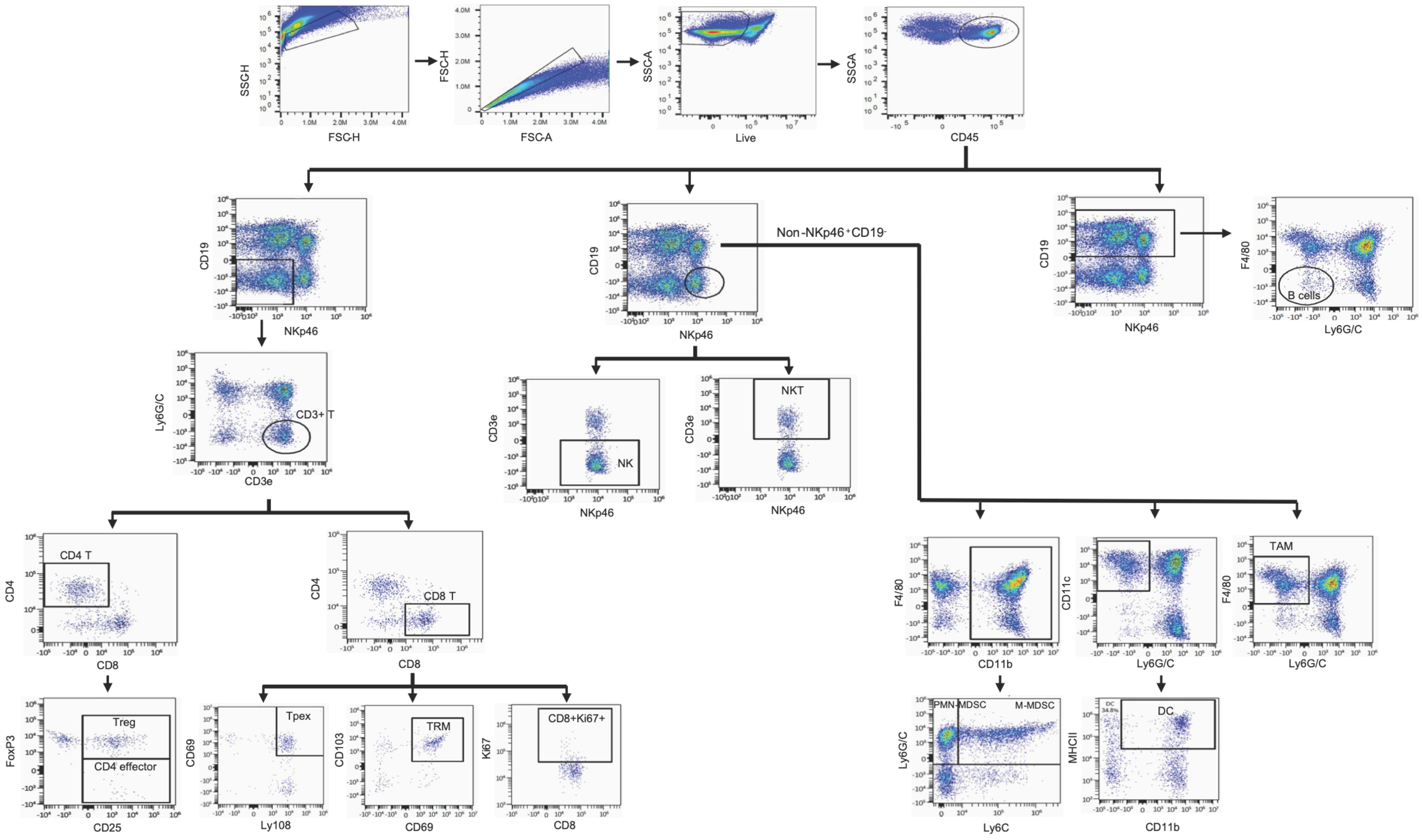

B

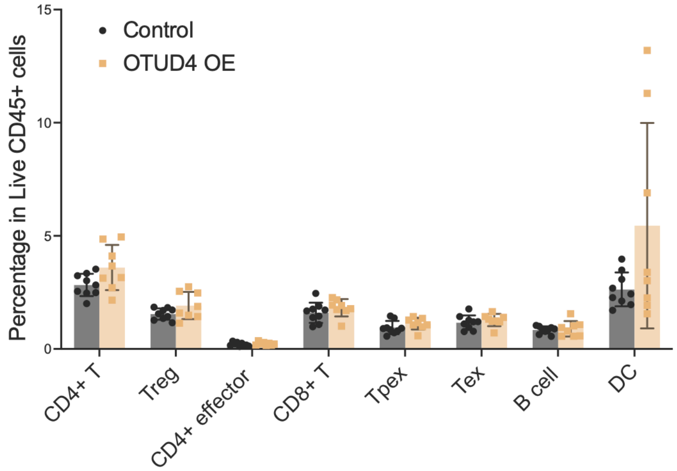

C

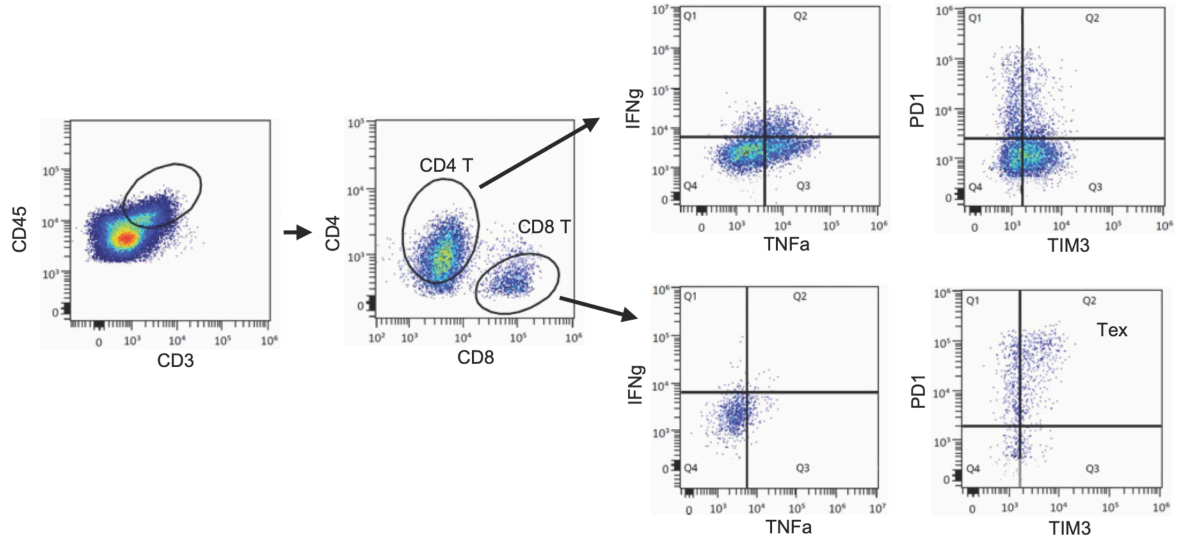

Figure S12

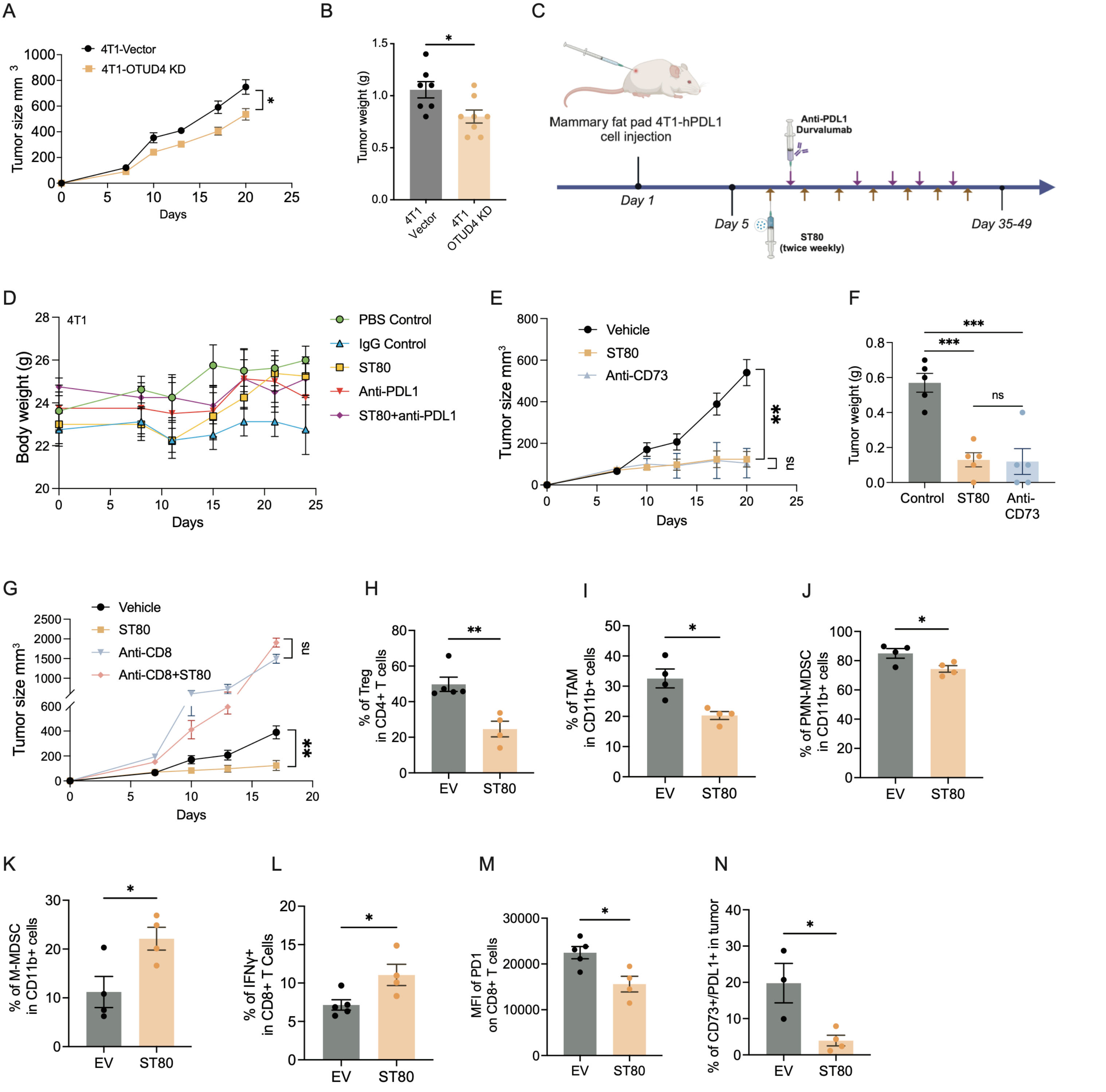
A
4T1-Vector
4T1-OTUD4 KD
Tumor size mm 3
Days
*
B
Tumor weight (g)
4T1 Vector
4T1 OTUD4 KD
*
C
Mammary fat pad 4T1-hPDL1 cell injection
Anti-PDL1 Durvalumab
Day 1
Day 5
ST80 (twice weekly)
Day 35-49
D
4T1
Body weight (g)
Days
PBS Control
IgG Control
ST80
Anti-PDL1
ST80+anti-PDL1
E
Vehicle
ST80
Anti-CD73
Tumor size mm3
Days
**
ns
F
Tumor weight (g)
Control
ST80
Anti-CD73
***
***
ns
G
Vehicle
ST80
Anti-CD8
Anti-CD8+ST80
Tumor size mm3
Days
ns
**
H
% of Treg in CD4+ T cells
EV
ST80
**
I
% of TAM in CD11b+ cells
EV
ST80
*
J
% of PMN-MDSC in CD11b+ cells
EV
ST80
*
K
% of M-MDSC in CD11b+ cells
EV
ST80
*
L
% of IFNγ+ in CD8+ T Cells
EV
ST80
*
M
MFI of PD1 on CD8+ T cells
EV
ST80
*
N
% of CD73+/PDL1+ in tumor
EV
ST80
*

Figure S13

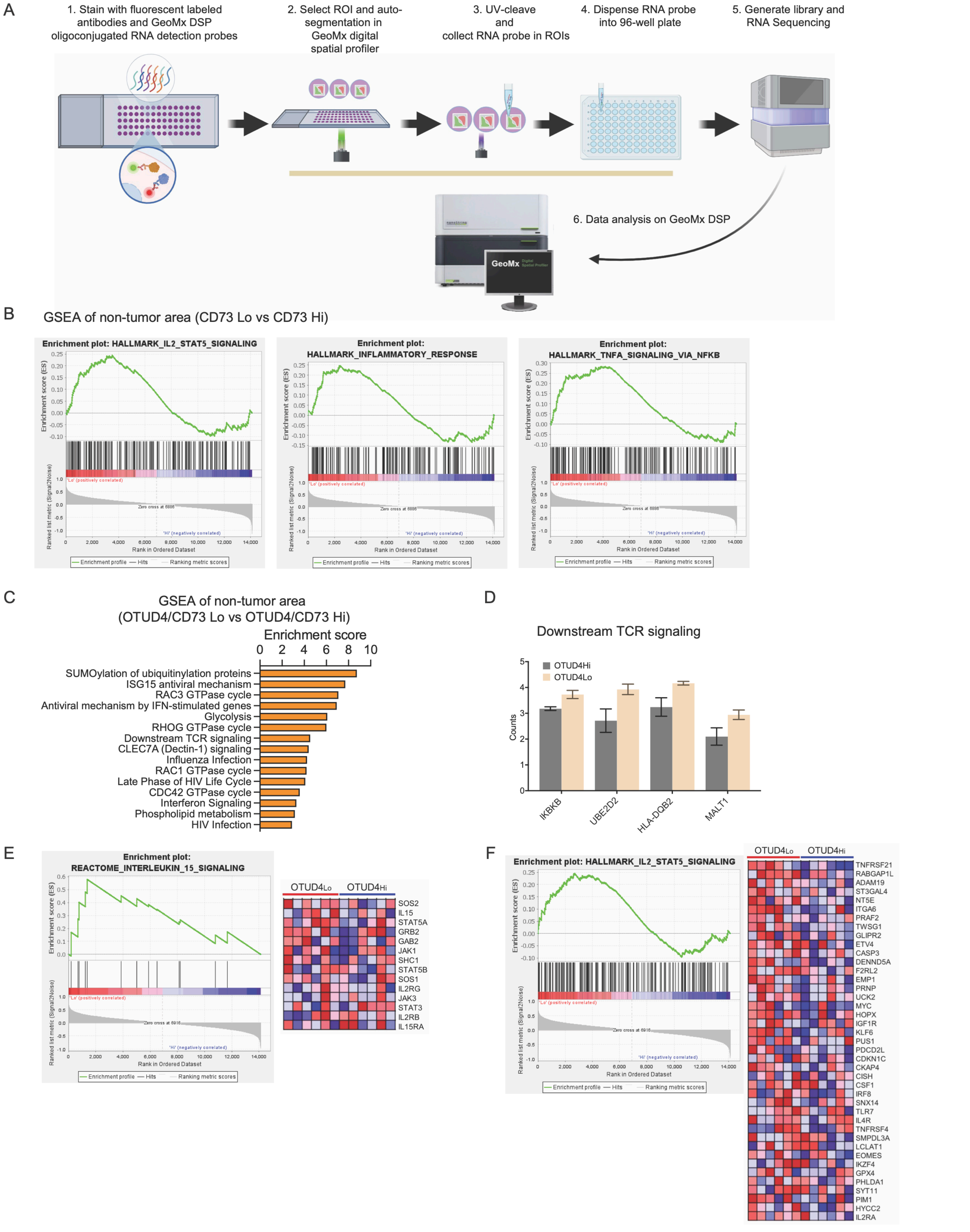
A
1. Stain with fluorescent labeled antibodies and GeoMx DSP oligoconjugated RNA detection probes
2. Select ROI and auto-segmentation in GeoMx digital spatial profiler
3. UV-cleave and collect RNA probe in ROIs
4. Dispense RNA probe into 96-well plate
5. Generate library and RNA Sequencing
6. Data analysis on GeoMx DSP
GeoMx
B
GSEA of non-tumor area (CD73 Lo vs CD73 Hi)
Enrichment plot: HALLMARK_IL2_STAT5_SIGNALING
Enrichment plot: HALLMARK_INFLAMMATORY_RESPONSE
Enrichment plot: HALLMARK_TNFA_SIGNALING_VIA_NFKB
Enrichment score (ES)
Ranked list metric (Signal2Noise)
'Lo' (positively correlated)
'Hi' (negatively correlated)
Zero cross at 6886
Rank in Ordered Dataset
Enrichment profile
Hits
Ranking metric scores
C
GSEA of non-tumor area (OTUD4/CD73 Lo vs OTUD4/CD73 Hi)
Enrichment score
0 2 4 6 8 10
SUMOylation of ubiquitinylation proteins
ISG15 antiviral mechanism
RAC3 GTPase cycle
Antiviral mechanism by IFN-stimulated genes
Glycolysis
RHOG GTPase cycle
Downstream TCR signaling
CLEC7A (Dectin-1) signaling
Influenza Infection
RAC1 GTPase cycle
Late Phase of HIV Life Cycle
CDC42 GTPase cycle
Interferon Signaling
Phospholipid metabolism
HIV Infection
D
Downstream TCR signaling
OTUD4Hi
OTUD4Lo
Counts
0 1 2 3 4 5
IKBKB
UBE2D2
HLA-DQB2
MALT1
E
Enrichment plot: REACTOME_INTERLEUKIN_15_SIGNALING
Enrichment score (ES)
Ranked list metric (Signal2Noise)
'Lo' (positively correlated)
'Hi' (negatively correlated)
Zero cross at 6916
Rank in Ordered Dataset
Enrichment profile
Hits
Ranking metric scores
OTUD4Lo
OTUD4Hi
SOS2
IL15
STAT5A
GRB2
GAB2
JAK1
SHC1
STAT5B
SOS1
IL2RG
JAK3
STAT3
IL2RB
IL15RA
F
Enrichment plot: HALLMARK_IL2_STAT5_SIGNALING
Enrichment score (ES)
Ranked list metric (Signal2Noise)
'Lo' (positively correlated)
'Hi' (negatively correlated)
Zero cross at 6916
Rank in Ordered Dataset
Enrichment profile
Hits
Ranking metric scores
OTUD4Lo
OTUD4Hi
TNFRSF21
RABGAP1L
ADAM19
ST3GAL4
NT5E
ITGA6
PRAF2
TWSG1
GLIPR2
ETV4
CASP3
DENND5A
F2RL2
EMP1
PRNP
UCK2
MYC
HOPX
IGF1R
KLF6
PUS1
PDCD2L
CDKN1C
CKAP4
CISH
CSF1
IRF8
SNX14
TLR7
IL4R
TNFRSF4
SMPDL3A
LCLAT1
EOMES
IKZF4
GPX4
PHLDA1
SYT11
PIM1
HYCC2
IL2RA

Figure S14

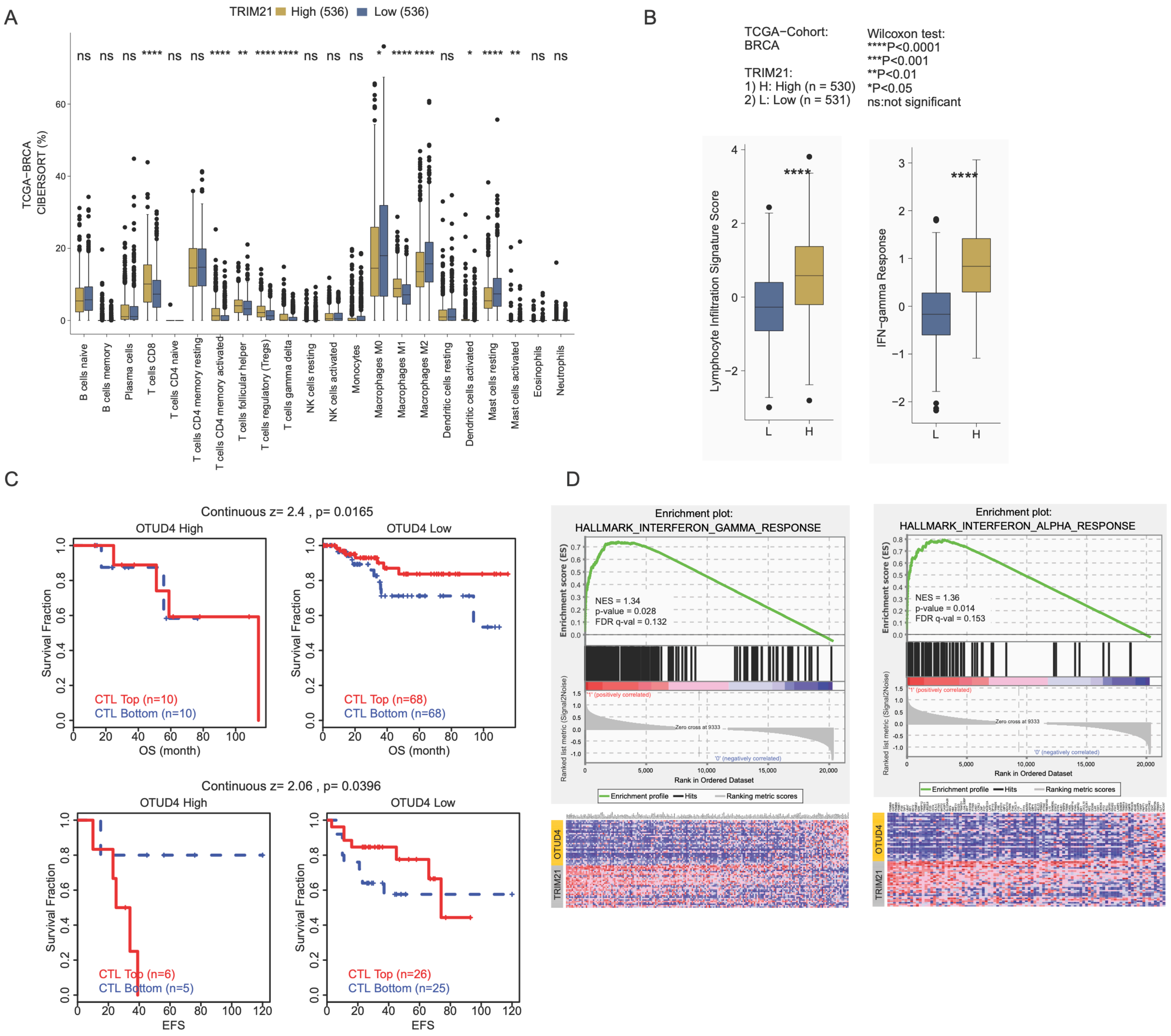

A
TRIM21 High (536) Low (536)
TCGA−BRCA CIBERSORT (%)
B cells naive
B cells memory
Plasma cells
T cells CD8
T cells CD4 naive
T cells CD4 memory resting
T cells CD4 memory activated
T cells follicular helper
T cells regulatory (Tregs)
T cells gamma delta
NK cells resting
NK cells activated
Monocytes
Macrophages M0
Macrophages M1
Macrophages M2
Dendritic cells resting
Dendritic cells activated
Mast cells resting
Mast cells activated
Eosinophils
Neutrophils
B
TCGA−Cohort: BRCA
TRIM21:
1) H: High (n = 530)
2) L: Low (n = 531)
Wilcoxon test:
****P<0.0001
***P<0.001
**P<0.01
*P<0.05
ns:not significant
Lymphocyte Infiltration Signature Score
IFN−gamma Response
C
Continuous z= 2.4 , p= 0.0165
OTUD4 High
OTUD4 Low
Survival Fraction
CTL Top (n=10)
CTL Bottom (n=10)
CTL Top (n=68)
CTL Bottom (n=68)
OS (month)
Continuous z= 2.06 , p= 0.0396
CTL Top (n=6)
CTL Bottom (n=5)
CTL Top (n=26)
CTL Bottom (n=25)
EFS
D
Enrichment plot: HALLMARK_INTERFERON_GAMMA_RESPONSE
NES = 1.34
p-value = 0.028
FDR q-val = 0.132
Enrichment plot: HALLMARK_INTERFERON_ALPHA_RESPONSE
NES = 1.36
p-value = 0.014
FDR q-val = 0.153
Enrichment score (ES)
Ranked list metric (Signal2Noise)
Rank in Ordered Dataset
'1' (positively correlated)
'0' (negatively correlated)
Zero cross at 9333
Enrichment profile
Hits
Ranking metric scores
OTUD4
TRIM21

Figure S15

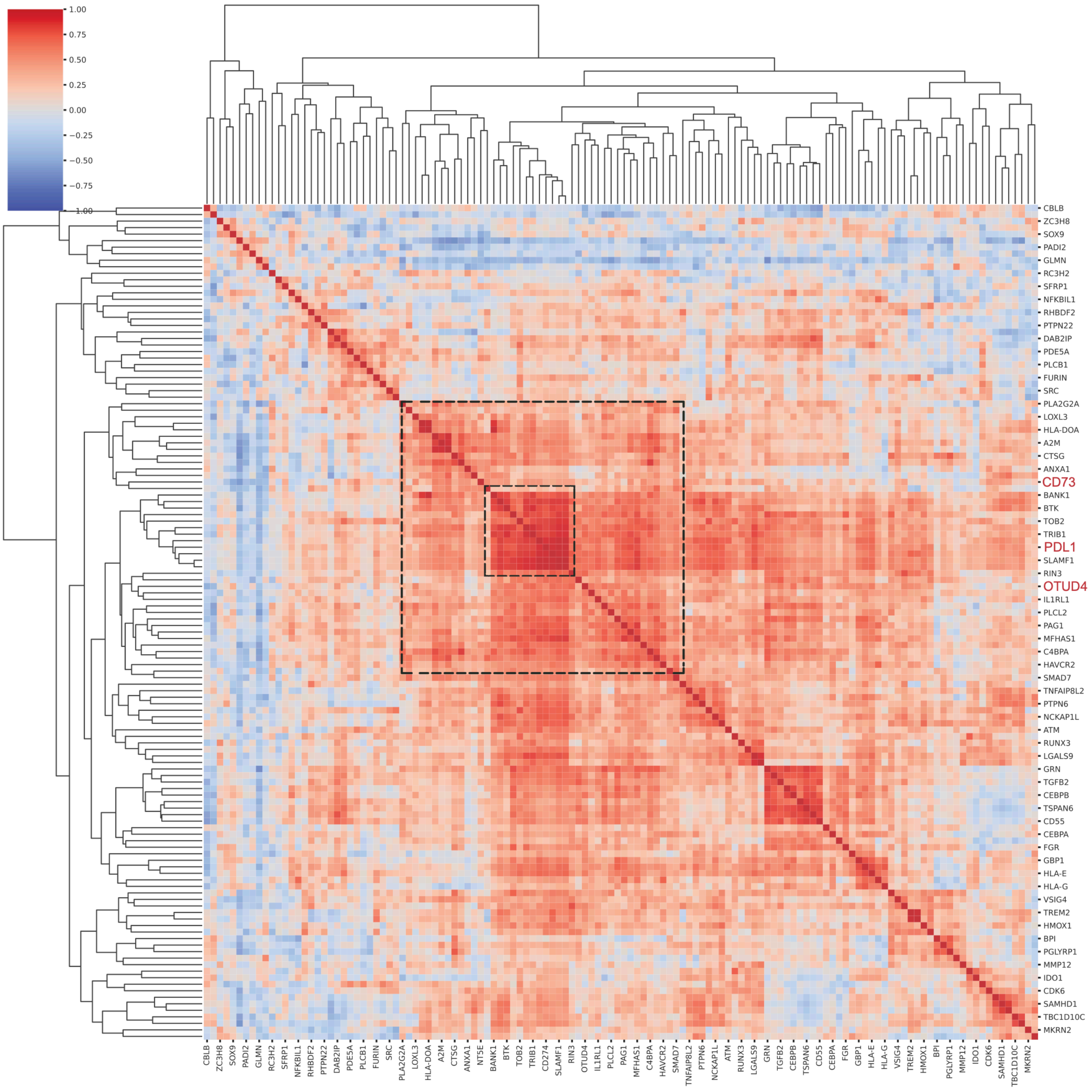

1.00
0.75
0.50
0.25
0.00
−0.25
−0.50
−0.75
−1.00
CBLB
ZC3H8
SOX9
PADI2
GLMN
RC3H2
SFRP1
NFKBIL1
RHBDF2
PTPN22
DAB2IP
PDE5A
PLCB1
FURIN
SRC
PLA2G2A
LOXL3
HLA-DOA
A2M
CTSG
ANXA1
CD73
BANK1
BTK
TOB2
TRIB1
PDL1
SLAMF1
RIN3
OTUD4
IL1RL1
PLCL2
PAG1
MFHAS1
C4BPA
HAVCR2
SMAD7
TNFAIP8L2
PTPN6
NCKAP1L
ATM
RUNX3
LGALS9
GRN
TGFB2
CEBPB
TSPAN6
CD55
CEBPA
FGR
GBP1
HLA-E
HLA-G
VSIG4
TREM2
HMOX1
BPI
PGLYRP1
MMP12
IDO1
CDK6
SAMHD1
TBC1D10C
MKRN2
CBLB
ZC3H8
SOX9
PADI2
GLMN
RC3H2
SFRP1
NFKBIL1
RHBDF2
PTPN22
DAB2IP
PDE5A
PLCB1
FURIN
SRC
PLA2G2A
LOXL3
HLA-DOA
A2M
CTSG
ANXA1
NT5E
BANK1
BTK
TOB2
TRIB1
CD274
SLAMF1
RIN3
OTUD4
IL1RL1
PLCL2
PAG1
MFHAS1
C4BPA
HAVCR2
SMAD7
TNFAIP8L2
PTPN6
NCKAP1L
ATM
RUNX3
LGALS9
GRN
TGFB2
CEBPB
TSPAN6
CD55
CEBPA
FGR
GBP1
HLA-E
HLA-G
VSIG4
TREM2
HMOX1
BPI
PGLYRP1
MMP12
IDO1
CDK6
SAMHD1
TBC1D10C
MKRN2

Figure S16

Table 1. Chemicals and Antibodies used in this study.

Chemical	Vendors	Catalog no.
Cycloheximide	Cayman	14126
MG-132	Cayman	13697
**Antibody**	**Vendors**	**Catalog no.**
Ubiquitin Polyclonal Anti-Rabbit	Cell Signaling	58395
CD73 Monoclonal Anti-Rabbit	Cell Signaling	13160
OTUD4 Rabbit Polyclonal	Abcam	Ab106368
β-Actin Monoclonal Anti-Rabbit	Cell Signaling	4970
CD73 Monoclonal Anti-Rabbit	Cell Signaling	13160 s
CD3 Rabbit Polyclonal	Viocare Medical	PP215AA
CD8 Monoclonal Anti-Mouse	Cell Signaling	70306S
Ki67 Rabbit Polyclonal	Abcam	ab15580
PanCK Mouse Monoclonal	Abcam	ab7753
CD4-BV750 (GK1.5) Rat Monoclonal Anti-Mouse	Biolegend	100467
CD8a-Spark Blue550 (53-6.7) Rat Monoclonal Anti-Mouse	Biolegend	100780
CD45-BV570 (30-F11) Rat Monoclonal Anti-Mouse	Biolegend	103135
CD3e-Percp5.5 (145-2C11) Armenian Hamster Monoclonal Anti-Mouse	Biolegend	100328
CD11b-BB515 (M1/70) Rat Monoclonal Anti-Mouse	BD Horizon	564454
CD11c-AF532 (N418) Armenian Hamster Monoclonal	eBiosciences	58011482
CD19-BV785 (6D5) Rat Monoclonal Anti-Mouse	Biolegend	115543
CD24-AF700 (M1/69) Rat Anti-Mouse	Biolegend	101836
CD25-BV650 (PC61) Rat Monoclonal Anti-Mouse	Biolegend	102038
CD44-PECy5 (IM7) Rat Monoclonal Anti-Mouse/Human	Biolegend	103010
CD69-BV510 (H1.2F3) Armenian Hamster Monoclonal Anti-Mouse	Biolegend	104532
CD73-APC (TY/11.8) Rat Monoclonal Anti-Mouse	Biolegend	127210
IFN-γ-PECy7 (XMG1.2) Rat Monoclonal Anti-Mouse	Biolegend	505826
TNF-α-BV650 (MP6-XT22) Rat Monoclonal Anti-Mouse	Biolegend	506333
F4/80-PECy5 (BM8) Rat Monoclonal Anti-Mouse	Biolegend	123114
MHCII-PE/Dazzle™ 594 (M5/114.15.2)	Biolegend	121606
CD163-PE (S15049F) Rat Monoclonal Anti-Mouse	Biolegend	156704
CD274-BV605 (10F.9G2) Rat Monoclonal Anti-Mouse	Biolegend	124321
CD279-APC-R700 (J43) Armenian Hamster Monoclonal Anti-Mouse	BD Biosciences	565815

Ly-6C-BV711 (HK1.4) Rat Monoclonal Anti-Mouse	Biolegend	128037
Ly-6G/C-AF594 (RB6-8C5) Rat Monoclonal Anti-Mouse	Biolegend	108448
Tim3-APC (B8.2C12) Rat Monoclonal Anti-Mouse	Biolegend	134008
CD335(NKp46)-PerCp-eFluor710 Rat Monoclonal Anti-Mouse	eBiosciences	46-3351-82
CX3CR1-APC/Fire750 (SA011F11) Mouse Monoclonal	Biolegend	149039
CD86-FITC (GL-1) Rat Monoclonal Anti-Mouse	Biolegend	105006
FoxP3-BV421 (MF-14) Rat Monoclonal Anti-Mouse	Biolegend	126419
Ly108-BB700 (13G3) Mouse Monoclonal	BD Biosciences	742272
Ki67-PacificBlue (16A8) Rat Monoclonal Anti-Mouse	Biolegend	652422
Tox-eFluor660 (TXRX10) Rat Monoclocal Anti-Human/Mouse	eBiosciences	50650282
CD103-BV480 (M290) Rat Anti-Mouse	BD Biosciences	566118
KLRG1-PECy7 (2F1/KLRG1) Syrian Hamster Monoclonal Anti-Mouse/Human	Biolegend	138416
CD45-APC-Cy7 (30-F11) Rat Monoclonal Anti-Mouse	Biolegend	103116
CD8-BUV737 (53-6.7) Rat Monoclonal Anti-Mouse	BD Biosciences	612759
CD4-FITC (GK1.5) Rat Monoclonal Anti-Mouse	Biolegend	100406
CD279 (PD1)-PE (29F.1A12) Rat Monoclonal Anti-Mouse	Biolegend	135206
PanCK-AF532 (AE1/AE3) Mouse Monoclonal	Novus	NBP2-33200AF532
CD73-CoraLite594 (1G5G8) Mouse Monoclonal Anti-Human	Proteintech	CL594-67789

Table 2: Full Protein ID list of Figure 1A and Figure 1B

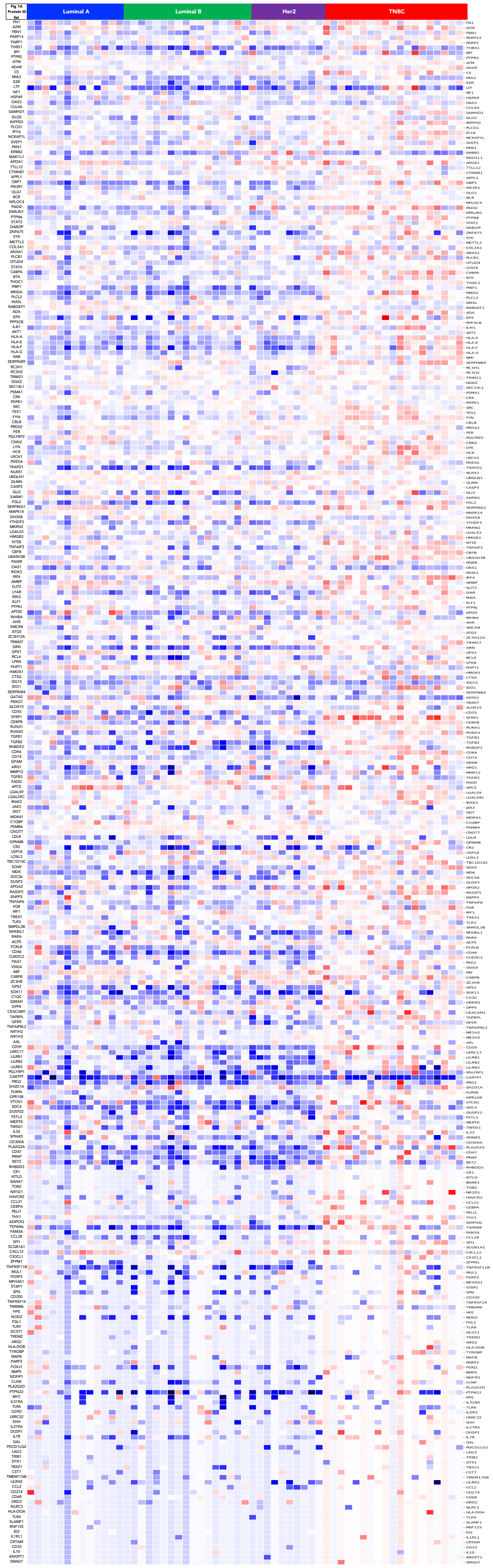

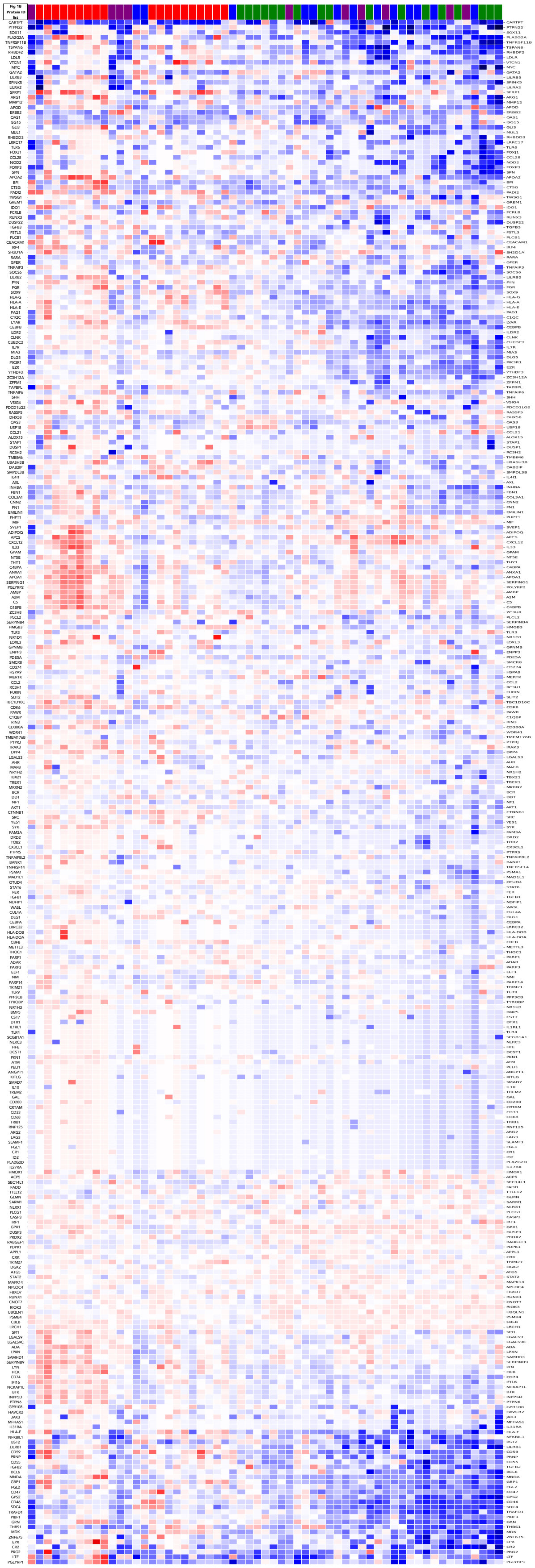
Fig 1B
Protein ID
list

Gene	Log2 Fold Change (Group1 vs Group2)	P-value (Group1 vs Group2)	Adjusted P-value (FDR)
CXCL12	4.008663964	3.15201E-05	0.009739716
LTF	3.576554218	0.163980915	0.386077432
IL33	3.401000273	0.009081497	0.063828349
APOA1	3.294575386	0.00575973	0.049437679
**NT5E**	**3.277011899**	**0.000103599**	**0.011889078**
PLA2G2A	3.007236226	0.233084376	0.473836
APOA2	3.00164891	0.03847658	0.16066572
C1QC	2.891544221	0.001458219	0.030608955
A2M	2.836624089	0.018030521	0.105121337
SERPING1	2.812597861	0.005686127	0.049437679
APOD	2.743509881	0.004225232	0.042116019
SERPINB9	2.419027331	0.017876676	0.105121337
APCS	2.400764627	0.044715143	0.165357524
C4BPB	2.399461087	0.050055227	0.175762103
AMBP	2.359354708	0.013103121	0.084351342
ANXA1	2.352005331	0.019847211	0.112226998
COL3A1	2.344243209	0.031098167	0.141912117
SVEP1	2.327702953	0.019975679	0.112226998
GREM1	2.267456461	0.030719267	0.141912117
THY1	2.263149207	0.030057641	0.141912117
ADIPOQ	2.235325833	0.068981223	0.224370503
CTSG	2.22117756	0.213670239	0.455338647
C5	2.208304853	0.023709475	0.122103796
SH2D1A	2.147519862	0.125836426	0.311067646
CCL21	2.131479753	0.074610398	0.235948136
PRNP	2.118330149	0.083846351	0.249120409
ADA	2.100004068	0.001883069	0.030608955
TLR3	2.096547049	0.006931775	0.056366272
FBN1	2.051239954	0.064435137	0.211813375
PGLYRP2	2.048600698	0.027334231	0.136230278
TBC1D10C	1.974699323	0.044162699	0.165357524
SAMHD1	1.917925935	0.007846	0.060583462
INPP5D	1.825257184	0.011096768	0.074541335
SYK	1.785452597	0.008038582	0.060583462
CDK6	1.731793431	0.031229851	0.141912117
VSIG4	1.719667191	0.25282374	0.50479333
BTK	1.70629047	0.021580791	0.116739955
C4BPA	1.702891095	0.17524426	0.401114639
LPXN	1.604398797	0.090509558	0.263843899
IRF4	1.596583062	0.308058842	0.557186455
GPAM	1.584910213	0.081814415	0.247460093
GPX1	1.582818538	0.00217042	0.030608955
SPI1	1.566820235	0.042853571	0.165357524
EMILIN1	1.557299962	0.041834957	0.165357524
FER	1.555732314	0.006800672	0.056366272
PTPN6	1.552585435	0.116345434	0.301645207
HLA-DOA	1.531455128	0.171447885	0.397911378
SFRP1	1.522827575	0.294041695	0.547342674

LGALS9C	1.495825851	0.04495156	0.165357524
PHPT1	1.474810906	0.031025332	0.141912117
CD74	1.417229039	0.074831448	0.235948136
AXL	1.40633081	0.033842786	0.14728762
RASSF5	1.385078999	0.121752645	0.308373502
LGALS9	1.374009644	0.055687262	0.191192931
DUSP3	1.367898576	0.06011459	0.201906612
TNFAIP8L2	1.363306165	0.042780553	0.165357524
LYN	1.332916626	0.092177545	0.266194968
PRG2	1.332461311	0.523033975	0.742272405
HAVCR2	1.31456974	0.043584591	0.165357524
GPNMB	1.304373297	0.18167992	0.40760914
FGL2	1.232817045	0.263552296	0.515428225
HMOX1	1.229982398	0.184715463	0.40980336
HCK	1.223565419	0.247600434	0.500055778
CNN2	1.20121653	0.137169444	0.333742979
MYC	1.187521781	0.022290153	0.116739955
PDCD1LG2	1.178562659	0.12415869	0.311067646
HLA-DOB	1.16575142	0.31190868	0.557186455
MNDA	1.161877195	0.445437038	0.67680359
SMAD7	1.154167178	0.108005191	0.282827154
APPL1	1.118816699	0.004430509	0.042782105
PKN1	1.109402151	0.001260011	0.030608955
STAT6	1.095972738	0.021959609	0.116739955
TAPBPL	1.063665396	0.217137852	0.459558878
TWSG1	1.059334904	0.22202238	0.463783174
PLCB1	1.058411089	0.395183732	0.631725581
STAT2	1.04770973	0.107736057	0.282827154
NCKAP1L	1.036040556	0.185671425	0.40980336
MAFB	1.036025476	0.11714377	0.301645207
RIN3	1.024761519	0.014159116	0.087503338
LILRB2	1.015423353	0.3686017	0.599462764
LOXL3	1.015345254	0.2867379	0.54357062
ACP5	1.008717609	0.047560241	0.170623434
IFI16	1.00764392	0.100922146	0.280945433
BANK1	1.007531589	0.063668566	0.211543944
BMP5	0.994588988	0.179115278	0.406960447
CD300A	0.988689754	0.312517999	0.557186455
FN1	0.988368534	0.315558672	0.557186455
PARP3	0.966501706	0.001280523	0.030608955
EPX	0.95668316	0.669094422	0.835967732
LRRC32	0.928003177	0.043412444	0.165357524
PAG1	0.923995438	0.253213483	0.50479333
CD274	0.909587142	0.082156344	0.247460093
SARM1	0.902286882	0.105746993	0.282827154
TRIM21	0.884746262	0.087397843	0.257199368
BST2	0.872598123	0.396617355	0.631725581
CST7	0.867822089	0.035540994	0.150440645
IRAK3	0.86276221	0.276479995	0.530635519
AHR	0.856513356	0.135953223	0.333409094
RHBDD3	0.851543829	0.104355303	0.282827154
PGLYRP1	0.846473743	0.720482159	0.874969165

RUNX3	0.843705645	0.328276209	0.56042734
NR1H2	0.842170886	0.00033949	0.02098048
FSTL3	0.836286091	0.524644152	0.742272405
PPP3CB	0.831679886	0.040031436	0.164929515
PDE5A	0.808688307	0.376005579	0.606380774
SLIT2	0.808104311	0.429757235	0.66067157
LGALS3	0.802894534	0.209457094	0.449460014
CRTAM	0.801790135	0.004082803	0.042116019
NLRX1	0.798482092	0.082486698	0.247460093
TNFRSF14	0.796374575	0.048039608	0.170623434
RARA	0.767766208	0.223747938	0.463783174
NR1H3	0.766454918	0.000153904	0.011889078
ATM	0.762349421	0.015339554	0.09293965
CR2	0.75685582	0.632230146	0.810618735
TRIB1	0.746774796	0.00829126	0.060999985
ARG1	0.742568214	0.706836119	0.866715717
PARP14	0.742420965	0.225137463	0.463783174
INHBA	0.74092795	0.358786685	0.592861421
DPP4	0.731103608	0.311734876	0.557186455
CD200	0.728706408	0.044893989	0.165357524
SPINK5	0.72143089	0.712315158	0.869981755
NF1	0.714555746	0.182039033	0.40760914
ERBB2	0.706171208	0.460831584	0.687908016
MFHAS1	0.696126294	0.005240282	0.049068091
FAM3A	0.691489211	0.000140638	0.011889078
BCR	0.672826867	0.10747922	0.282827154
DCST1	0.669006278	0.004133321	0.042116019
LRCH1	0.66646374	0.120754849	0.308373502
CD68	0.661401202	0.005408117	0.049150242
MUL1	0.654361432	0.093216511	0.266702795
PTPRJ	0.651017373	0.449697632	0.677836919
TREX1	0.642843934	0.191822743	0.4203775
GATA2	0.634410845	0.511159827	0.740274702
CCL2	0.633906383	0.002612163	0.030608955
TYROBP	0.632473571	0.376780287	0.606380774
ARG2	0.631130687	0.013869337	0.08746174
FADD	0.627036181	0.094093576	0.26674234
CCL28	0.609153558	0.587000805	0.785208869
TMEM176B	0.608158416	0.566778728	0.768134329
GLI3	0.606948414	0.738425797	0.87984493
ELF1	0.595023003	0.097111165	0.272794092
CBLB	0.577270357	0.081313761	0.247460093
CASP3	0.571113611	0.145620712	0.348812402
CR1	0.555665612	0.002674569	0.030608955
KITLG	0.555665612	0.002674569	0.030608955
SCGB1A1	0.555665612	0.002674569	0.030608955
ZFPM1	0.555665612	0.002674569	0.030608955
TREM2	0.555665612	0.002674569	0.030608955
PLA2G2D	0.555665612	0.002674569	0.030608955
IL27RA	0.555665612	0.002674569	0.030608955
GAL	0.555665612	0.002674569	0.030608955
LAG3	0.555665612	0.002674569	0.030608955

DRD2	0.555665612	0.002674569	0.030608955
NLRC3	0.555665612	0.002674569	0.030608955
TLR4	0.555665612	0.002674569	0.030608955
SLAMF1	0.555665612	0.002674569	0.030608955
RNF125	0.555665612	0.002674569	0.030608955
ID2	0.555665612	0.002674569	0.030608955
CD33	0.555665612	0.002674569	0.030608955
SMCR8	0.53212908	0.32804844	0.56042734
ILDR2	0.524667244	0.0034922	0.03853892
PRDX2	0.490624236	0.511239601	0.740274702
RC3H2	0.487454722	0.314728985	0.557186455
PSMB4	0.48637266	0.106703163	0.282827154
IL7R	0.484675691	0.102405371	0.282529103
CD59	0.484069192	0.748412063	0.87984493
SPN	0.48302793	0.626460732	0.80957077
RABGEF1	0.481021397	0.172557038	0.397911378
PIBF1	0.479303813	0.312282799	0.557186455
RUNX1	0.469041699	0.293784262	0.547342674
SEC14L1	0.46594644	0.32709389	0.56042734
CRK	0.458665387	0.284330962	0.542334983
LRRC17	0.454322813	0.628792831	0.80957077
ANGPT1	0.450970372	0.026942571	0.136230278
PAWR	0.448701443	0.596046887	0.790465614
HFE	0.441652563	0.027817864	0.13644
SERPINB4	0.437241017	0.538371925	0.750215133
PIK3R1	0.426640073	0.319573564	0.56042734
SRC	0.412171692	0.485463833	0.714554308
IL1RL1	0.39311687	0.053594617	0.186075694
AKT1	0.388718956	0.427485007	0.660464336
SHH	0.372167174	0.559102794	0.761069442
PDPK1	0.365244117	0.334993143	0.56564416
TOB2	0.358705949	0.125496895	0.311067646
CLNK	0.357940612	0.621804376	0.807300639
NR1D1	0.355589861	0.829561189	0.907553027
PELI1	0.351214648	0.069956134	0.225171308
DUSP22	0.33191037	0.554097376	0.757593315
TGFB1	0.308560763	0.54207026	0.751119777
CX3CL1	0.302455266	0.411277048	0.645099532
TLR6	0.294069103	0.807880003	0.891553289
ZC3H12A	0.281384042	0.548512947	0.753291114
FGL1	0.274966764	0.440514759	0.673856735
MKRN2	0.271016166	0.525213807	0.742272405
GRN	0.268273403	0.780756821	0.888118388
DDT	0.256671727	0.647653967	0.820184737
NDFIP1	0.245445719	0.426585985	0.660464336
ISG15	0.24253216	0.834970416	0.907553027
MAPK14	0.238932614	0.289581603	0.545614117
DUSP1	0.232066023	0.886175864	0.940990866
PLCG1	0.217704594	0.658019859	0.829910761
ENPP3	0.212548379	0.784648285	0.888118388
IRF1	0.200504536	0.636339791	0.81251651
TRAFD1	0.191364606	0.771625369	0.886539495

PTPRS	0.190224208	0.796248479	0.890446577
OAS3	0.180558151	0.839355918	0.907553027
GPS2	0.175995046	0.802719184	0.890446577
FYN	0.174737075	0.788350788	0.888906858
TLR9	0.167904355	0.726215246	0.874969165
PLCL2	0.163072271	0.768608799	0.886539495
ATG5	0.150544557	0.570969323	0.770434589
OTUD4	0.150022818	0.647030173	0.820184737
MERTK	0.145639194	0.782507219	0.888118388
GFER	0.123912786	0.842937601	0.907553027
TBX21	0.117424705	0.773782074	0.886539495
TGFB2	0.100808712	0.832997545	0.907553027
METTL3	0.064851834	0.860414536	0.919958795
OAS1	0.063421648	0.945750569	0.974123086
CEACAM1	0.059128533	0.970436142	0.983163173
MIA3	0.043226742	0.955340694	0.978088117
IL10	0.043034876	0.93273003	0.964405452
LDLR	0.037826242	0.926689169	0.964405452
DGKZ	0.033334385	0.896354685	0.945302381
CBFB	0.018244773	0.976840753	0.986417623
BPI	-0.003273204	0.99871397	0.99871397
NMI	-0.007011321	0.993168176	0.996392748
LILRB1	-0.013605494	0.98562356	0.99204456
PSMA1	-0.016885771	0.959096115	0.978088117
WDR41	-0.023040607	0.965378804	0.981256745
EZR	-0.032494719	0.93319492	0.964405452
TNFAIP3	-0.037413763	0.955960058	0.978088117
MIF	-0.060889007	0.931828911	0.964405452
FURIN	-0.109925029	0.84107563	0.907553027
TMBIM6	-0.13592912	0.884898321	0.940990866
NOD2	-0.141532091	0.901463214	0.947456235
THBS1	-0.151779663	0.908701285	0.951826092
GPR108	-0.154447434	0.691090844	0.854188284
WASL	-0.159220196	0.750180421	0.87984493
NPLOC4	-0.167801105	0.607595368	0.798923271
IL31RA	-0.170391466	0.822022816	0.903932563
IDO1	-0.184696896	0.893233447	0.945236764
YES1	-0.189739662	0.74450365	0.87984493
DLG5	-0.212623983	0.751712173	0.87984493
HLA-E	-0.248465946	0.760710865	0.886539495
TGFB3	-0.264139038	0.737919452	0.87984493
CEBPA	-0.278618241	0.724526085	0.874969165
ZNF675	-0.280406405	0.851964158	0.914086545
UBQLN1	-0.30486736	0.224496654	0.463783174
STAP1	-0.307442908	0.769987665	0.886539495
TNFAIP6	-0.307566126	0.727725163	0.874969165
SMPDL3B	-0.313146121	0.702146051	0.864394939
UBASH3B	-0.318208253	0.750187678	0.87984493
RC3H1	-0.318380838	0.368541517	0.599462764
DHX58	-0.341809126	0.589863137	0.785636678
MMP12	-0.349229855	0.803603957	0.890446577
IL4I1	-0.35065739	0.689963417	0.854188284

DAB2IP	-0.35171109	0.526076559	0.742272405
DLG1	-0.371806249	0.454609988	0.681914982
SOCS6	-0.376277066	0.354662996	0.589198202
TTLL12	-0.381834005	0.538989513	0.750215133
FBXO7	-0.390243088	0.537328665	0.750215133
ALOX15	-0.397577391	0.791098337	0.888906858
USP18	-0.401150579	0.665477454	0.835904607
CUL4A	-0.402128339	0.231042469	0.472795516
RIOK3	-0.441985514	0.205476882	0.447129272
MAD1L1	-0.459941125	0.009088826	0.063828349
CEBPB	-0.522649734	0.326225371	0.56042734
THOC1	-0.544161731	0.260791718	0.513277967
SOX11	-0.555170036	0.774646161	0.886539495
DTX1	-0.557047366	0.512682156	0.740274702
PTPN22	-0.561973219	0.803995453	0.890446577
CUEDC2	-0.570154025	0.164926282	0.386077432
CD55	-0.582235754	0.158372493	0.376439233
GBP1	-0.610680076	0.670938504	0.835967732
RHBDF2	-0.618067615	0.573797286	0.77088418
GLMN	-0.622557729	0.265681873	0.51632515
ZC3H8	-0.637378171	0.367173067	0.599462764
HLA-A	-0.639341149	0.479261732	0.711980169
CD46	-0.735153176	0.488636049	0.715585494
ADAR	-0.741736013	0.270137788	0.521703603
FOXJ1	-0.803858499	0.403083067	0.635472794
C1QBP	-0.817071703	0.032994044	0.14728762
TNFRSF11B	-0.842480756	0.617316764	0.807270248
HSPA9	-0.857258122	0.056618062	0.192252542
CNOT7	-0.872101401	0.035192762	0.150440645
BCL6	-0.878435991	0.327053161	0.56042734
HLA-F	-0.88422996	0.446821788	0.67680359
PARP1	-0.885197551	0.141000982	0.340385184
SDC4	-0.897119015	0.517369612	0.742272405
VTCN1	-0.916335205	0.598669942	0.790551334
FGR	-0.936496234	0.402519	0.635472794
LYAR	-0.952079148	0.300682579	0.556352796
JAK3	-0.960150637	0.41863247	0.653320369
YTHDF3	-0.973719708	0.033393656	0.14728762
CD47	-1.00085794	0.31210461	0.557186455
MDK	-1.17245065	0.333699169	0.56564416
TSPAN6	-1.246674237	0.354600987	0.589198202
TRIM27	-1.271949774	0.046143146	0.167743906
FOXP3	-1.295966207	0.348212869	0.584770525
FCRLB	-1.300262807	0.209159942	0.449460014
CARTPT	-1.426826952	0.619168442	0.807270248
LILRB3	-1.551813039	0.548443471	0.753291114
CTNNB1	-1.613738376	0.021425145	0.116739955
HLA-G	-1.739921204	0.013082447	0.084351342
PADI2	-1.835166868	0.257485324	0.510019006
LILRA2	-1.870862488	0.485619433	0.714554308
SOX9	-2.007457739	0.011046933	0.074541335
NFKBIL1	-2.079124744	0.0805189	0.247460093

HMGB3	-2.419372128	0.007601515	0.060227388
