# Supplementary material for: Pharmacological suppression of the OTUD4/CD73 proteolytic axis revives antitumor immunity against immune-suppressive breast cancers
Source: J Clin Invest. 2024 Mar 26;134(10):e176390. doi: 10.1172/JCI176390 (PMC11093616; doi:10.1172/JCI176390)

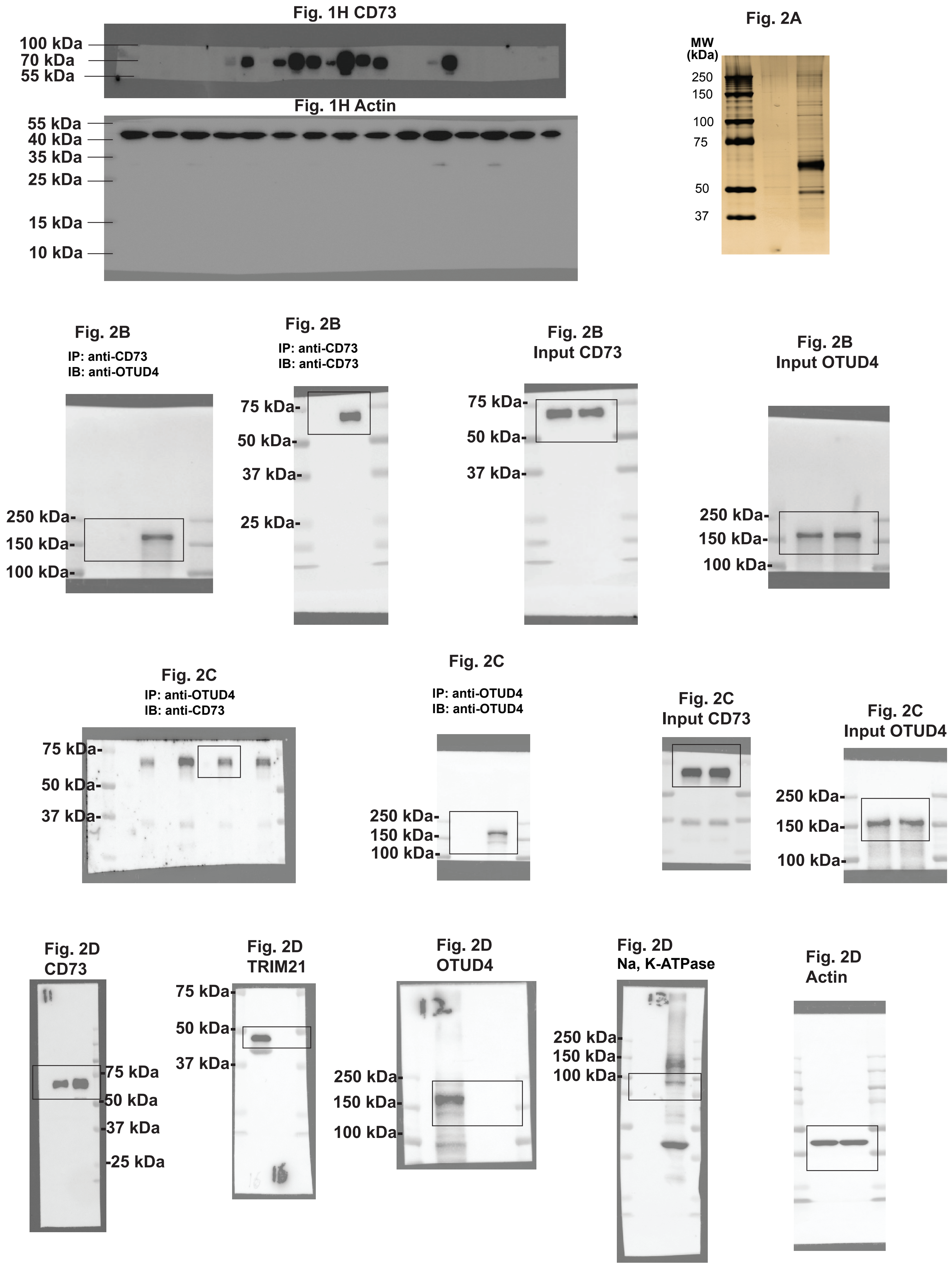
Fig. 1H CD73
100 kDa
70 kDa
55 kDa
Fig. 1H Actin
55 kDa
40 kDa
35 kDa
25 kDa
15 kDa
10 kDa
Fig. 2A
MW (kDa)
250
150
100
75
50
37
Fig. 2B
IP: anti-CD73
IB: anti-OTUD4
250 kDa-
150 kDa-
100 kDa-
Fig. 2B
IP: anti-CD73
IB: anti-CD73
75 kDa-
50 kDa-
37 kDa-
25 kDa-
Fig. 2B
Input CD73
75 kDa-
50 kDa-
37 kDa-
Fig. 2B
Input OTUD4
250 kDa-
150 kDa-
100 kDa-
Fig. 2C
IP: anti-OTUD4
IB: anti-CD73
75 kDa-
50 kDa-
37 kDa-
Fig. 2C
IP: anti-OTUD4
IB: anti-OTUD4
250 kDa-
150 kDa-
100 kDa-
Fig. 2C
Input CD73
Fig. 2C
Input OTUD4
250 kDa-
150 kDa-
100 kDa-
Fig. 2D
CD73
-75 kDa
-50 kDa
-37 kDa
-25 kDa
Fig. 2D
TRIM21
75 kDa-
50 kDa-
37 kDa-
Fig. 2D
OTUD4
250 kDa-
150 kDa-
100 kDa-
Fig. 2D
Na, K-ATPase
250 kDa-
150 kDa-
100 kDa-
Fig. 2D
Actin

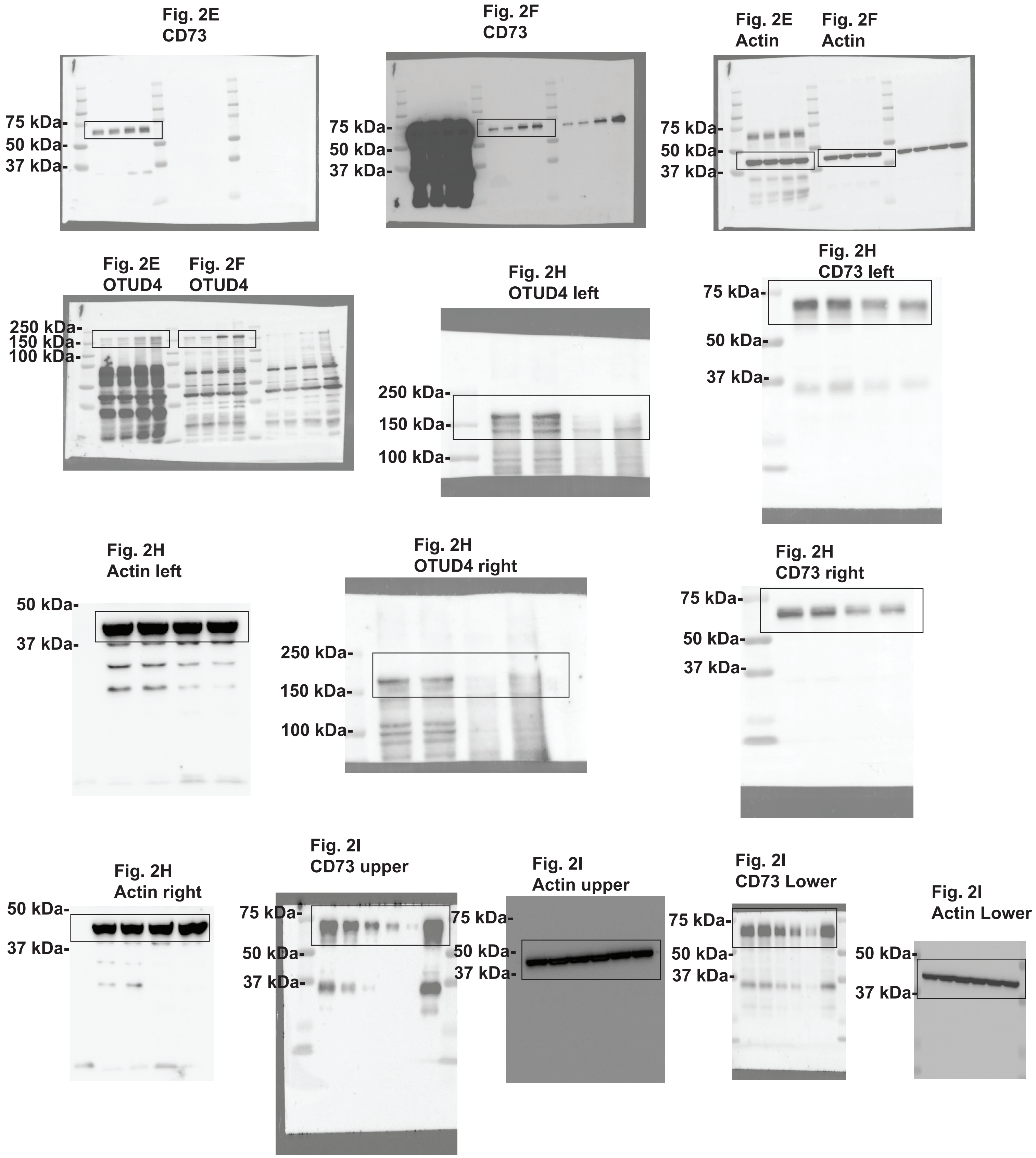
Fig. 2E
CD73
75 kDa-
50 kDa-
37 kDa-
Fig. 2F
CD73
75 kDa-
50 kDa-
37 kDa-
Fig. 2E
Actin
Fig. 2F
Actin
75 kDa-
50 kDa-
37 kDa-
Fig. 2E
OTUD4
Fig. 2F
OTUD4
250 kDa-
150 kDa-
100 kDa-
Fig. 2H
OTUD4 left
250 kDa-
150 kDa-
100 kDa-
Fig. 2H
CD73 left
75 kDa-
50 kDa-
37 kDa-
Fig. 2H
Actin left
50 kDa-
37 kDa-
Fig. 2H
OTUD4 right
250 kDa-
150 kDa-
100 kDa-
Fig. 2H
CD73 right
75 kDa-
50 kDa-
37 kDa-
Fig. 2H
Actin right
50 kDa-
37 kDa-
Fig. 2I
CD73 upper
75 kDa-
50 kDa-
37 kDa-
Fig. 2I
Actin upper
75 kDa-
50 kDa-
37 kDa-
Fig. 2I
CD73 Lower
75 kDa-
50 kDa-
37 kDa-
Fig. 2I
Actin Lower
50 kDa-
37 kDa-

Fig. 2J
IP: anti-CD73
IB: anti-Ub

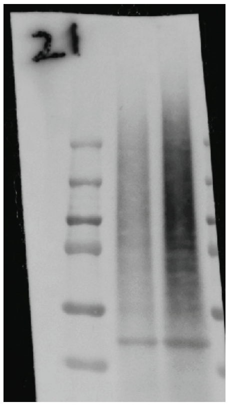

Fig. 2J
IP: anti-CD73
IB: anti-CD73

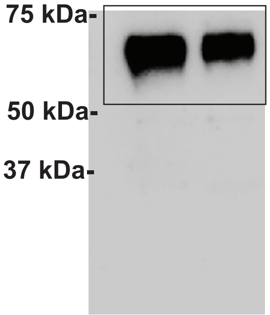

Fig. 2J
OTUD4

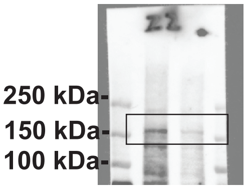

Fig. 2J
CD73

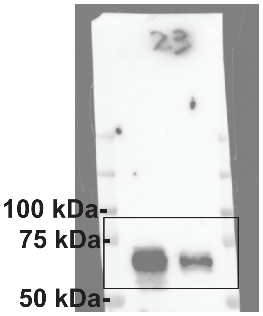

Fig. 2J
Actin

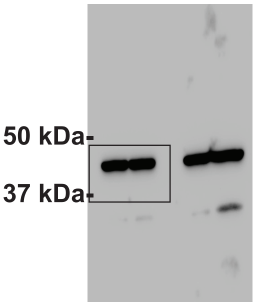

Fig. 3B
IP: Anti-V5
IB: Anti-Flag

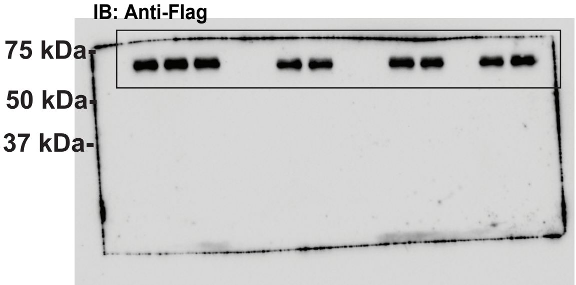

Fig. 3B
OTUD4 V5

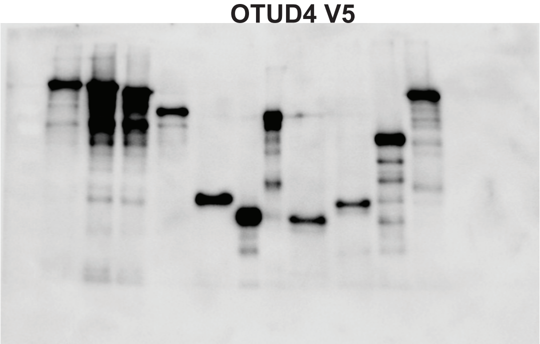

Fig. 3B
CD73 Flag

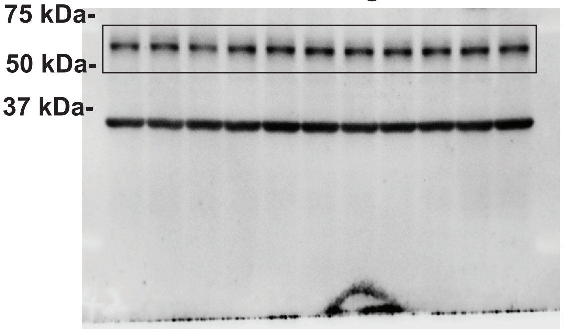

Fig. 3B
Actin

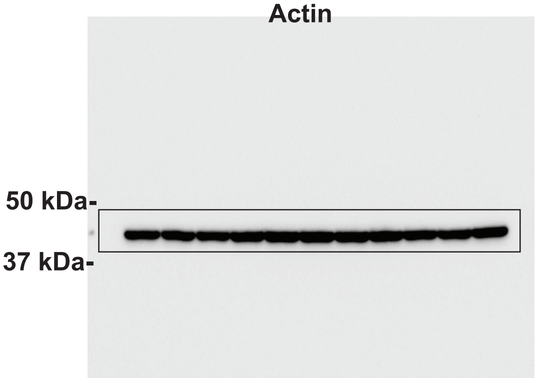

Fig. 3D
IP: Anti-V5
IB: Anti-V5

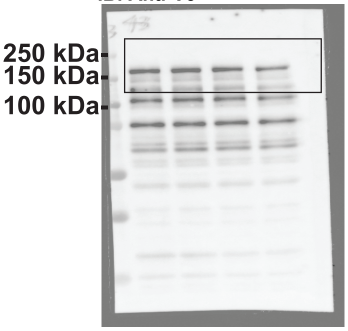

Fig. 3D
IP: Anti-V5
IB: Anti-Flag

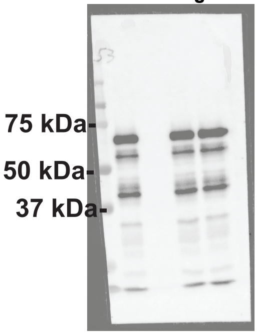

Fig. 3D
OTUD4 V5

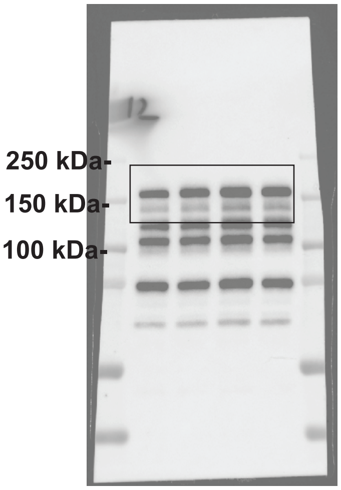

Fig. 3D
CD73-Flag

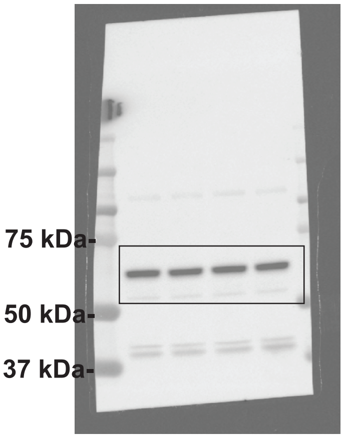

Fig. 3D
Actin

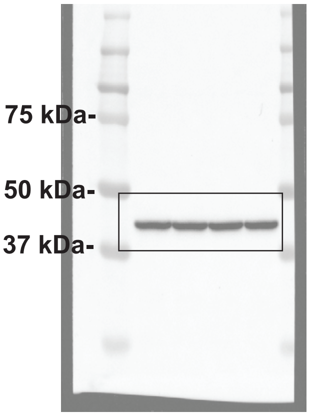

Fig. 3F

IP: Anti-Flag
IB: Anti-V5

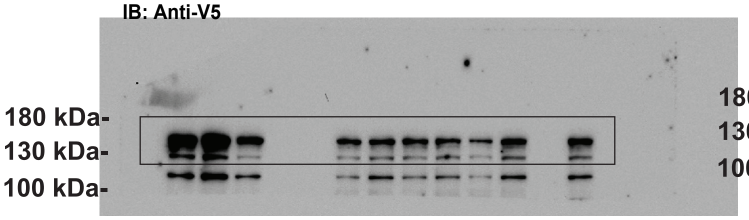

Fig. 3F
OTUD4-V5

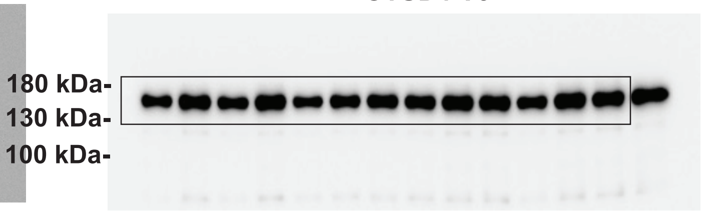

Fig. 3F
CD73-Flag

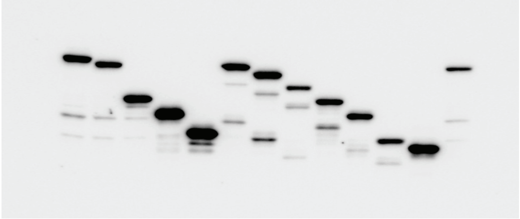

Fig. 3F
Actin

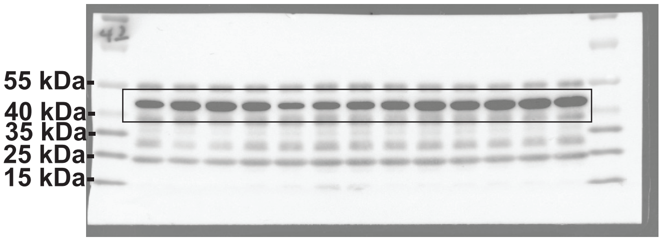

Fig. 3H
OTUD4

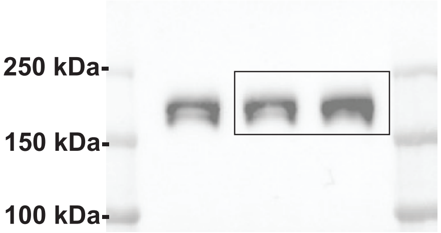

Fig. 3H
CD73-V5

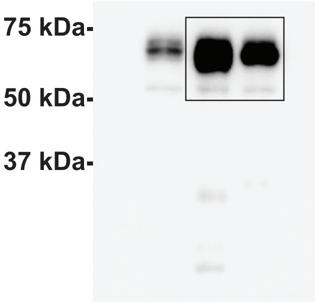

Fig. 3H
Actin

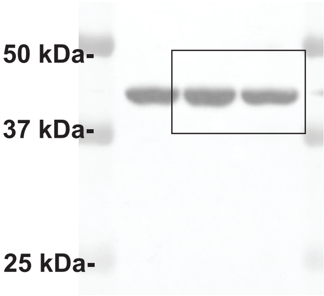

Fig. 3H

IP:CD73-V5
IB:OTUD4

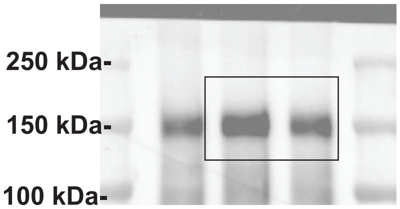

Fig. 3H

IP:CD73-V5
IB:V5

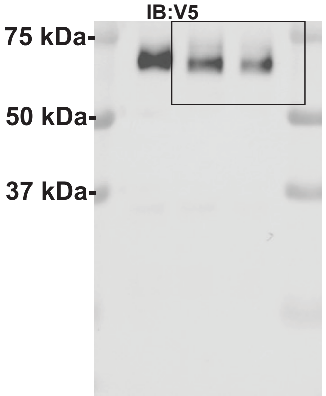

Fig. 3H

IP:CD73-V5
IB:Ub

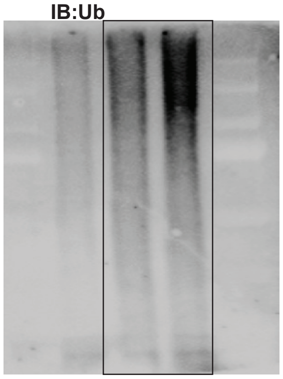

Fig. 3I
Upper CD73 V5

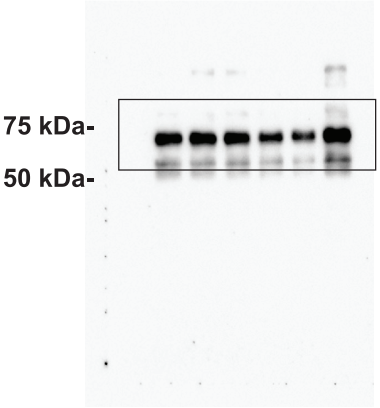

Fig. 3I
upper Actin

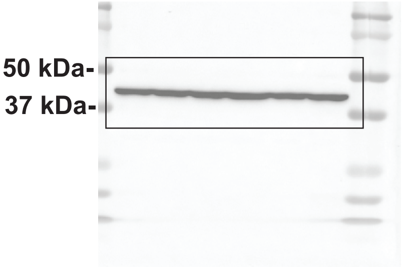

Fig. 3I
lower CD73 V5

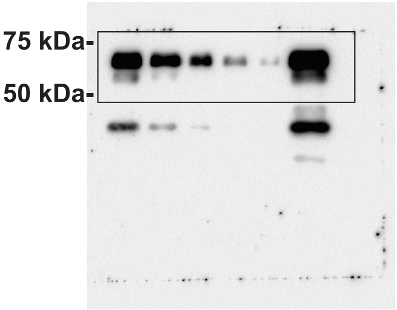

Fig. 3I
lower Actin

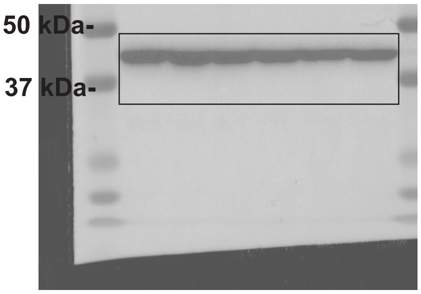

Fig. 4B
OTUD4

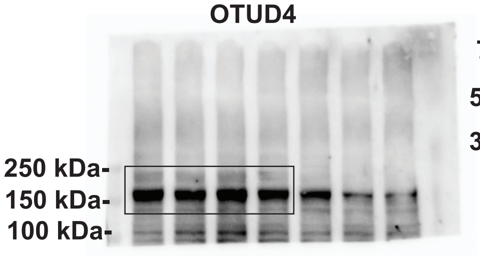

Fig. 4B
CD73

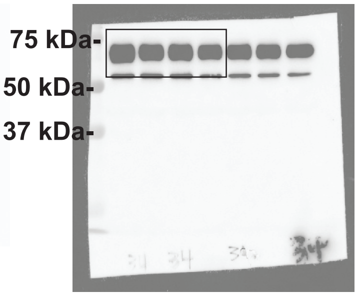

Fig. 4B
Actin

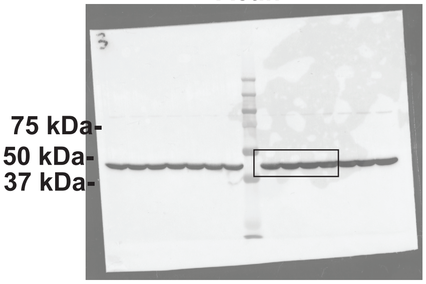

Fig. 4F
OTUD4

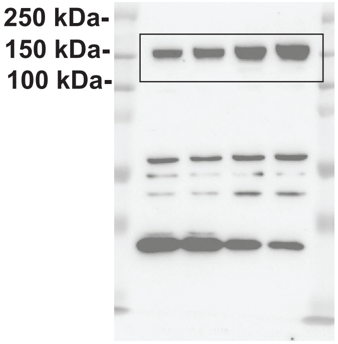

Fig. 4F
CD73

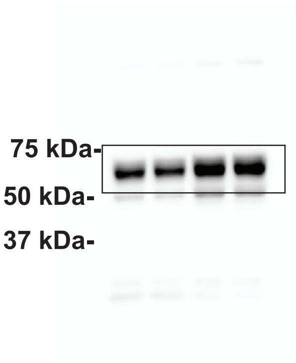

Fig. 4F
Actin

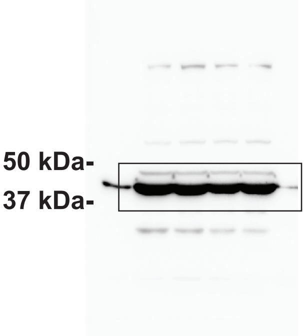

Fig. 4G
OTUD4

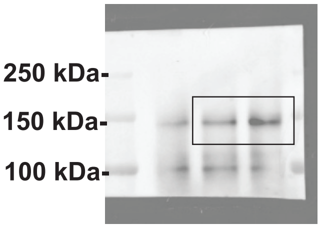

Fig. 4G
CD73

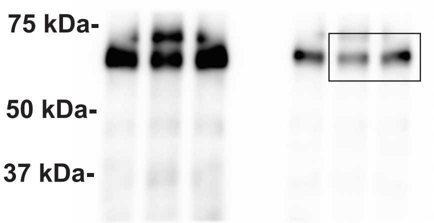

Fig. 4G
Actin

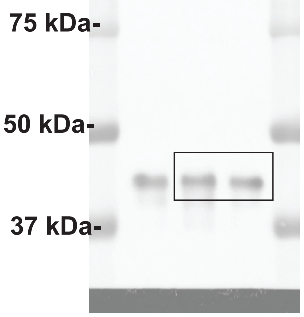

Fig. 4G
IP:CD73-V5
IB:V5

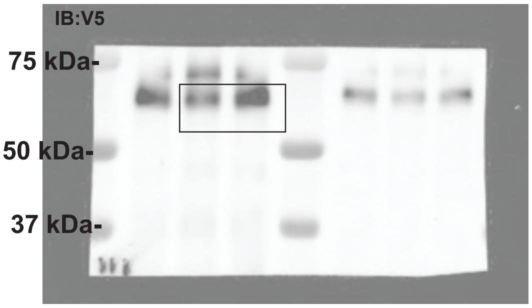

Fig. 4G
IP:CD73-V5
IB:Ub

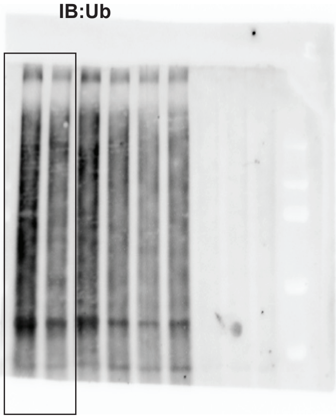

Fig. 4I
CD73 upper

Fig. 4I
CD73 lower

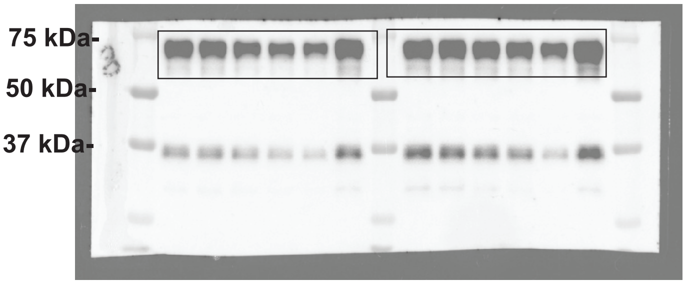

Fig. 4I
Actin upper

Fig. 4I
Actin lower

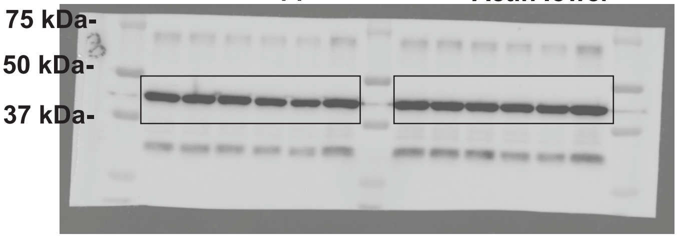

Fig. 4J
OTUD4

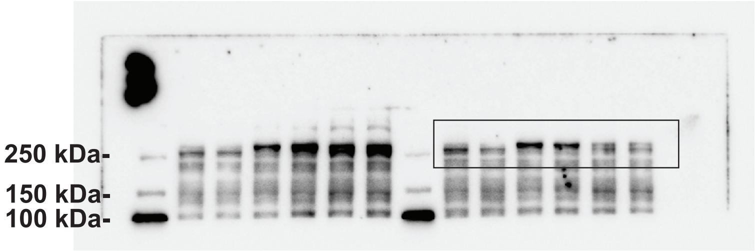

Fig. 4J
CD73

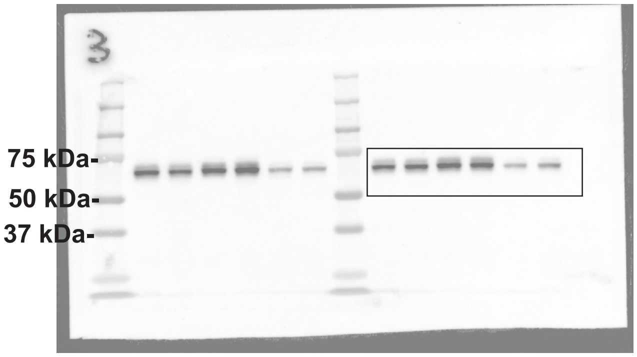

Fig. 4J
Actin

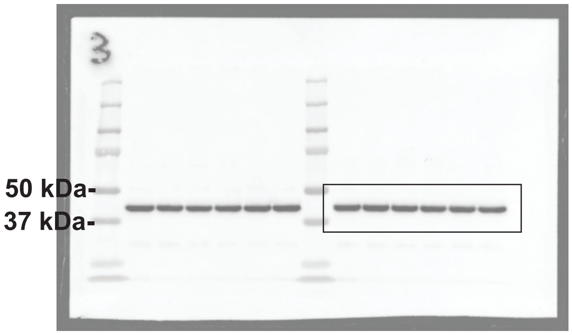

Fig. 4L
OTUD4

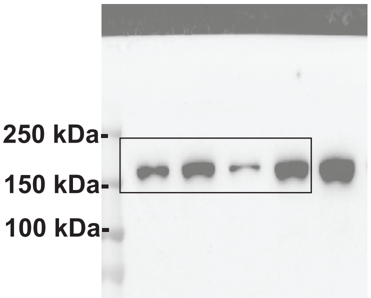

Fig. 4L
CD73-V5

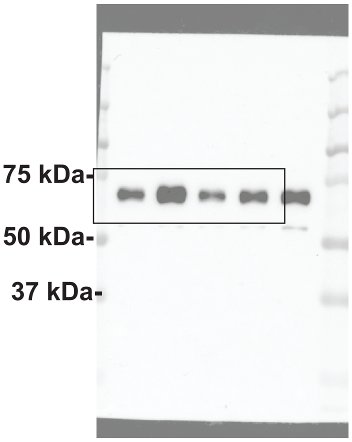

Fig. 4L
Actin

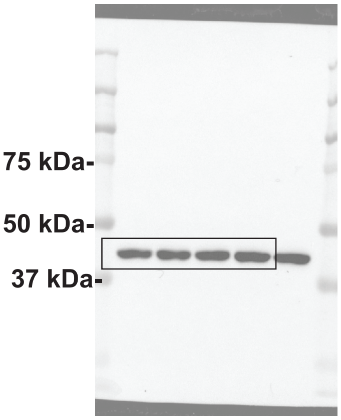

Fig. 4L

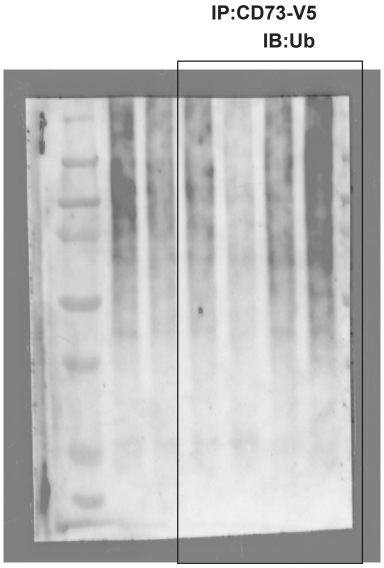

Fig. 4M
CD73

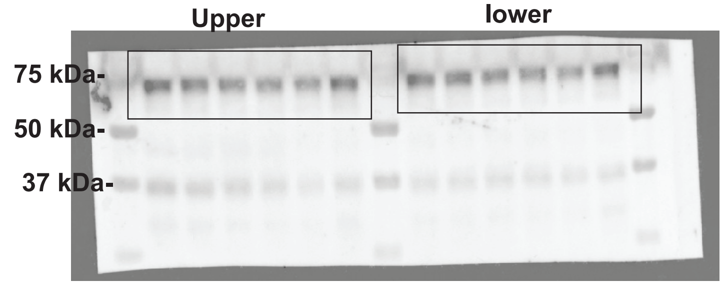

Fig. 4M
Upper Actin lower

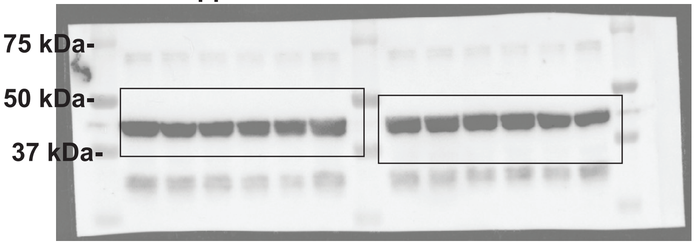

Fig. 5B
CD73

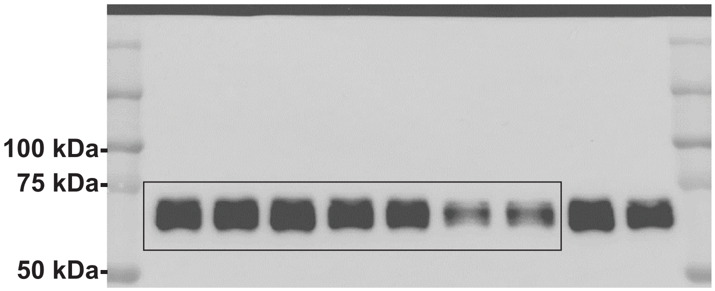

Fig. 5B
CD73

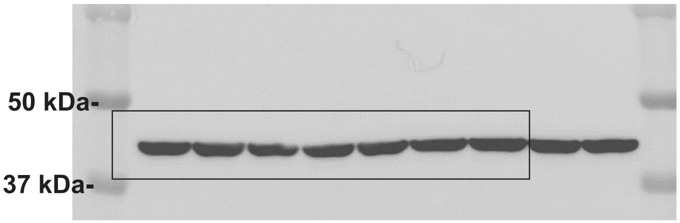

Fig. 5C
CD73

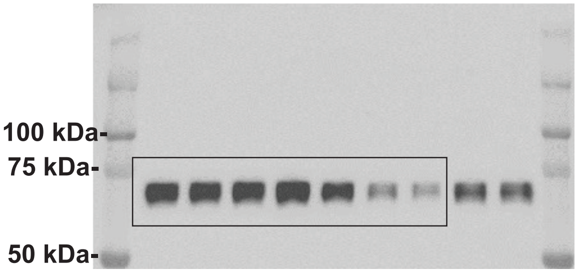

Fig. 5C
Actin

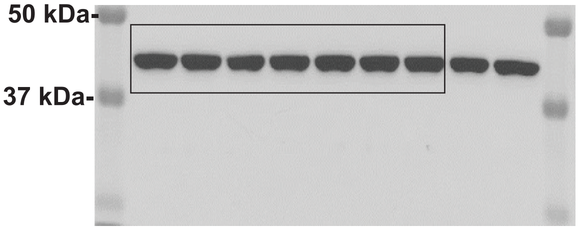

Fig. 5D
upper CD73

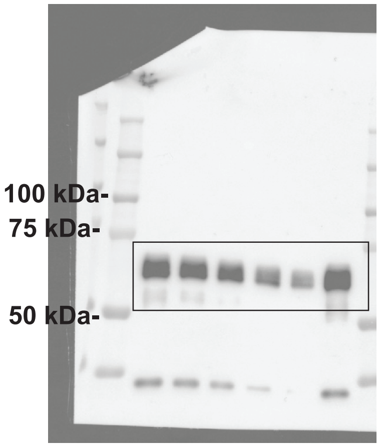

Fig. 5D
upper actin

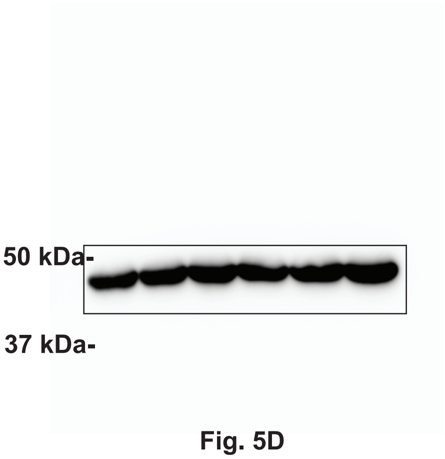

Fig. 5D
middle CD73

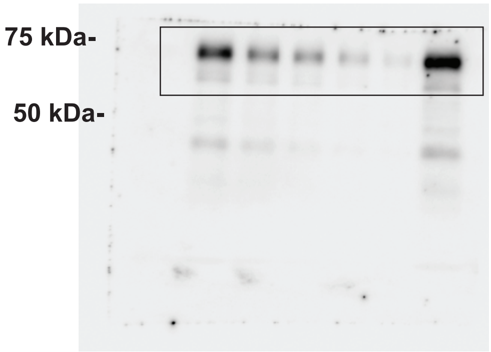

Fig. 5D
middle actin

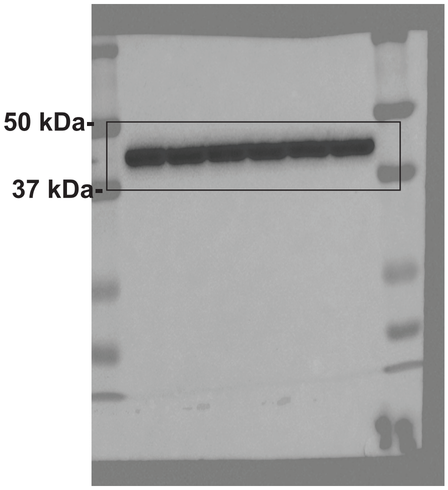

Fig. 5D
lower CD73

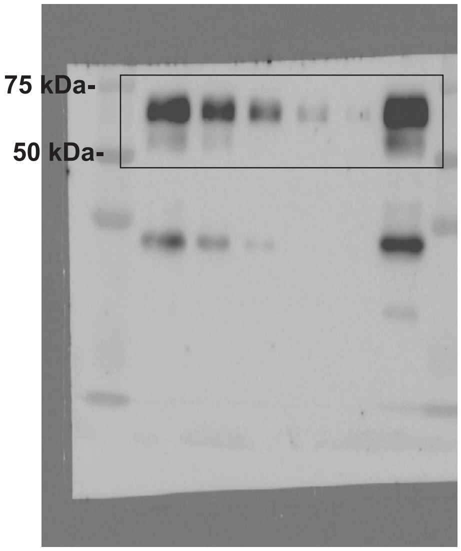

Fig. 5D
lower actin

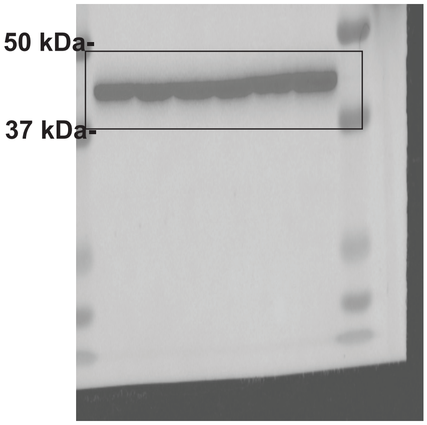

Fig. 5E
OTUD4

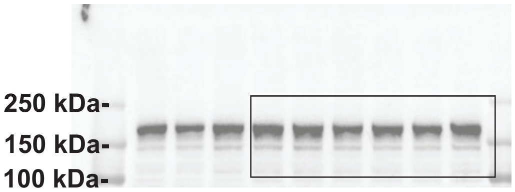

Fig. 5E
CD73-V5

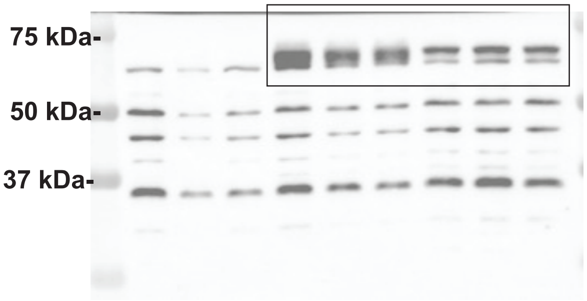

Fig. 5E
Actin

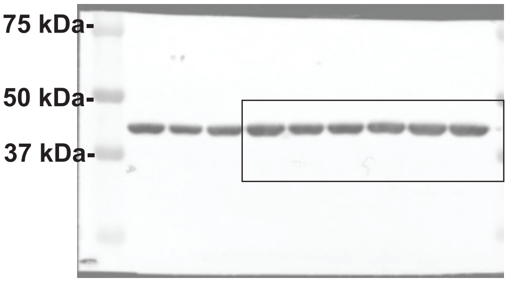

IP:CD73-V5
IB:OTUD4

Fig. 5E

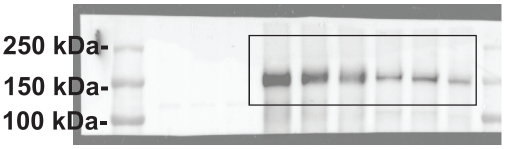

IP:CD73-V5
IB:V5

Fig. 5E

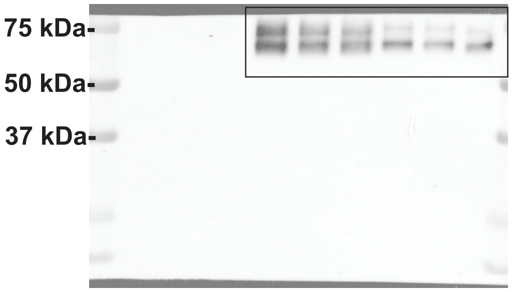

IP:CD73-V5
IB:Ub

Fig. 5E

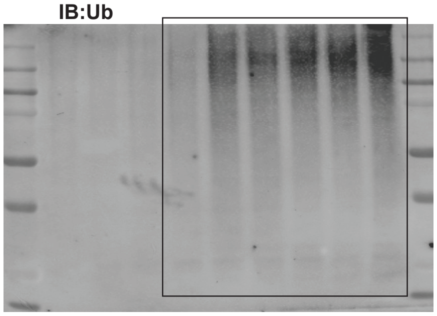

Fig. 5G
Upper CD73

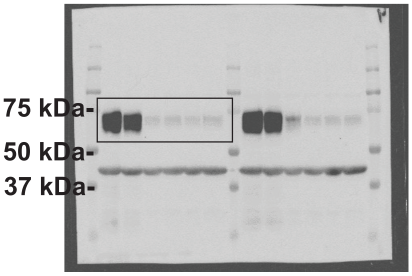

Fig. 5G
Upper actin

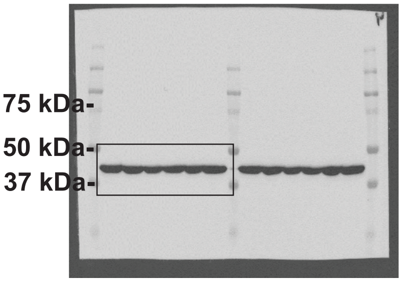

Fig. 5G
Lower CD73

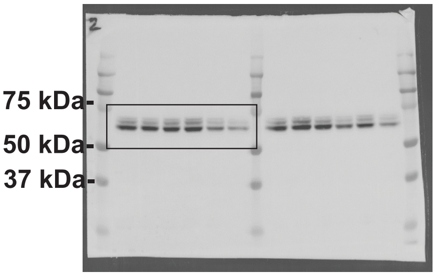

Fig. 5G
Lower actin

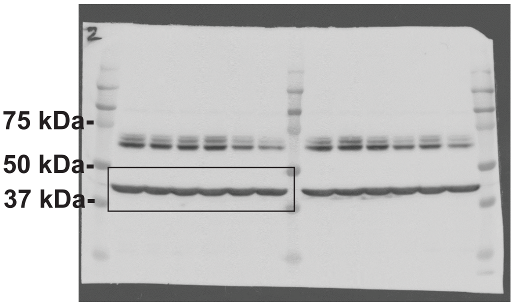

Fig. 5H
Upper CD73

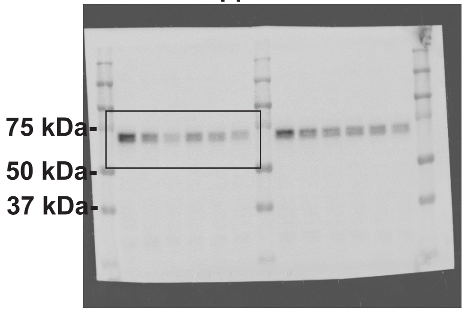

Fig. 5H
Upper actin

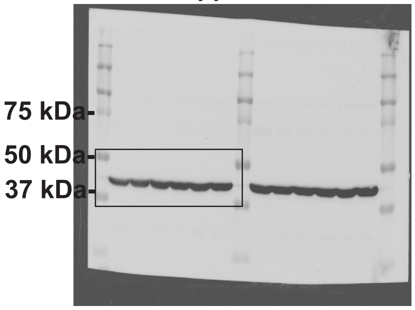

Fig. 5H
lower CD73

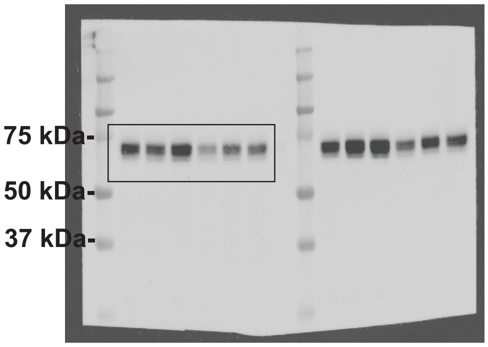

Fig. 5H
lower actin

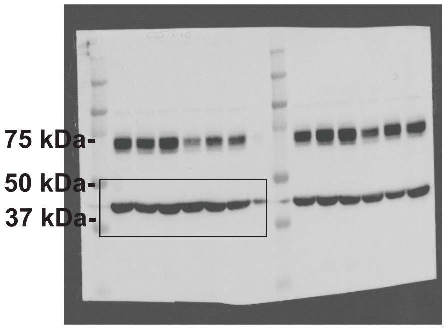

Fig. S4A
IP: anti-CD73
IB: anti-OTUD4

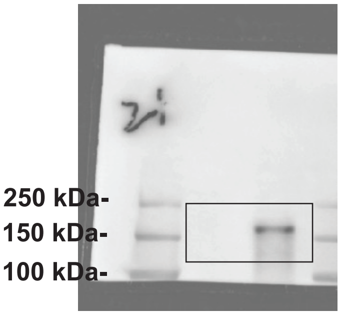

Fig. S4A
IP: anti-CD73
IB: anti-CD73

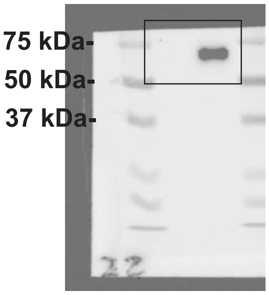

Fig. S4A
Input CD73

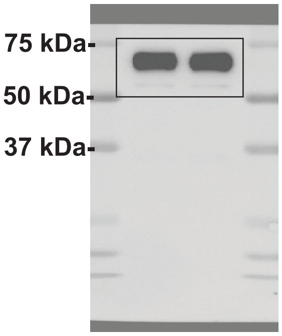

Fig. S4A
Input OTUD4

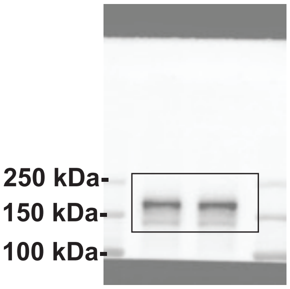

Fig. S4B
IP: anti-OTUD4
IB: anti-CD73

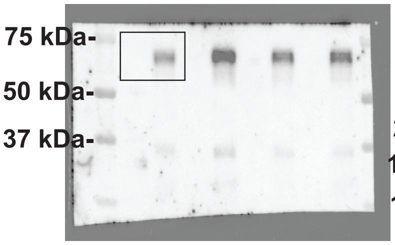

Fig. S4B
IP: anti-OTUD4
IB: anti-OTUD4

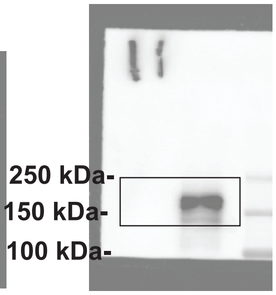

Fig. S4B
Input CD73

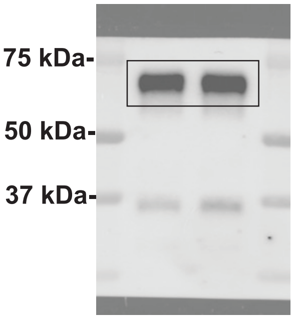

Fig. S4B
Input OTUD4

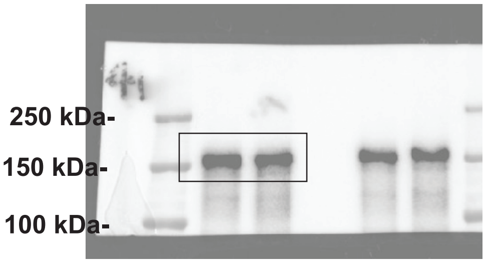

Fig. S4C
IP: anti-CD73
IB: anti-OTUD4

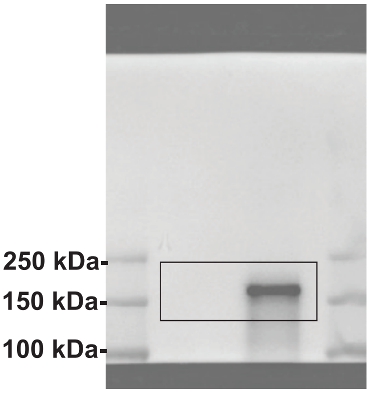

Fig. S4C
IP: anti-CD73
IB: anti-CD73

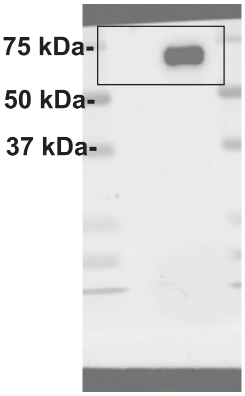

Fig. S4C
Input CD73

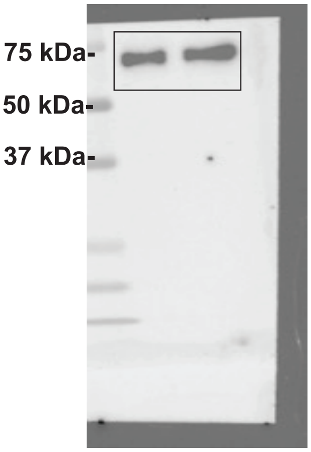

Fig. S4C
Input OTUD4

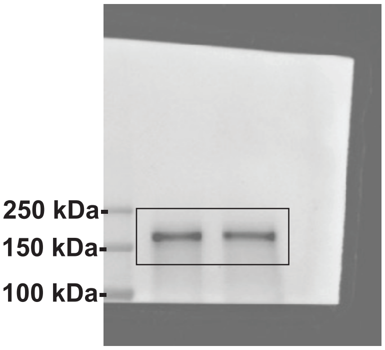

Fig. S4D
IP: anti-OTUD4
IB: anti-CD73

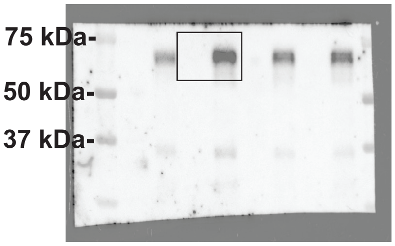

Fig. S4D
IP: anti-OTUD4
IB: anti-OTUD4

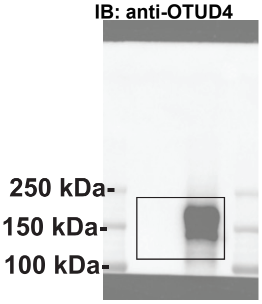

Fig. S4D
Input CD73

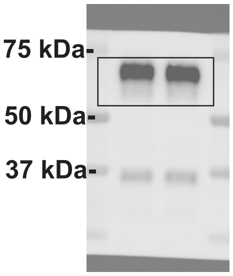

Fig. S4D
Input OTUD4

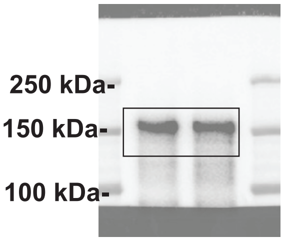

Fig. S6B

IP: anti-CD73-Flag
IB: anti-OTUD-V5

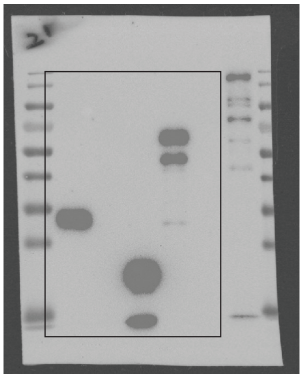

Fig. S6B
Input CD73-Flag

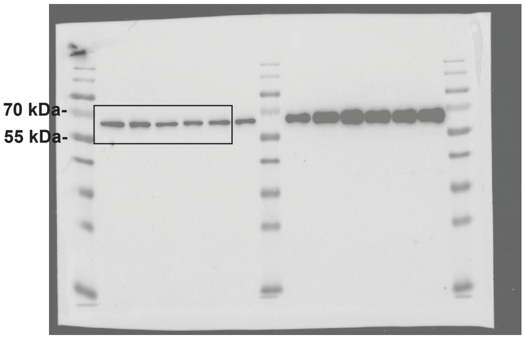

Fig. S6B
Input actin

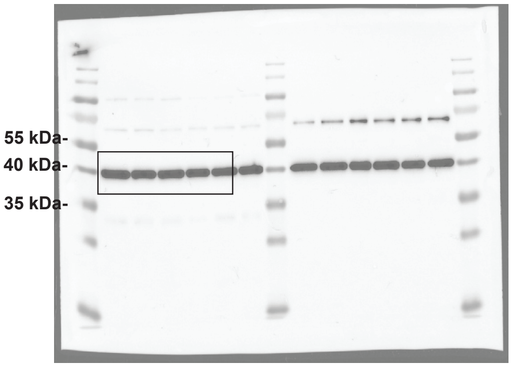

Fig. S6H
OTUD4

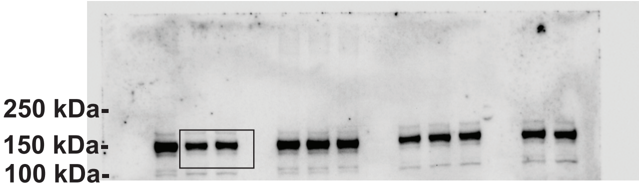

Fig. S6H
CD73

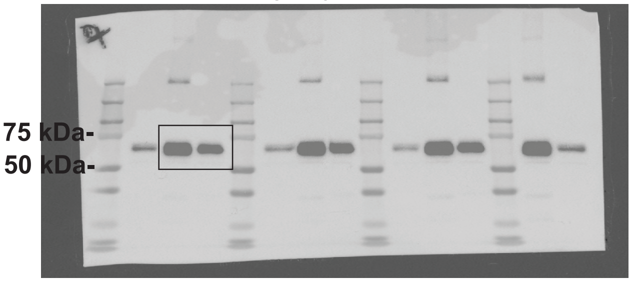

Fig. S6H
Actin

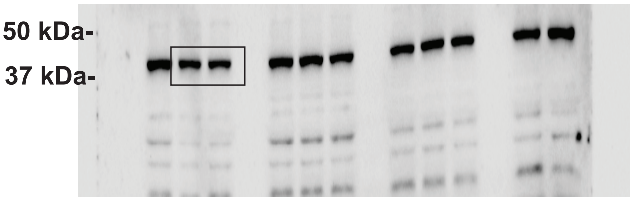

Fig. S6H

IP:CD73-V5
IB:OTUD4

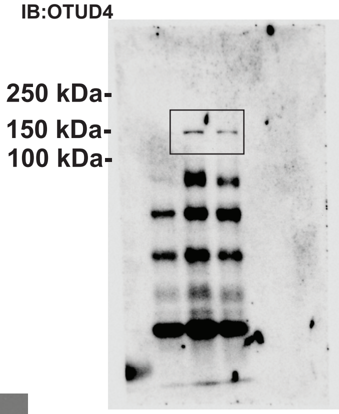

Fig. S6H

IP:CD73-V5
IB:V5

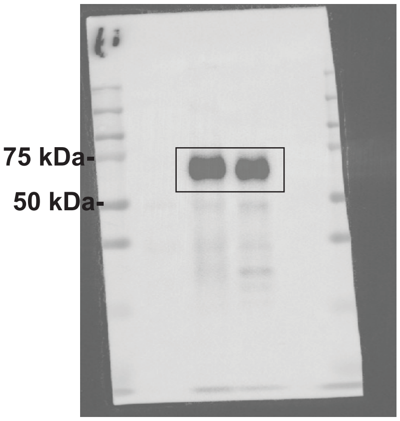

Fig. S6H

IP:CD73-V5
IB:Ub

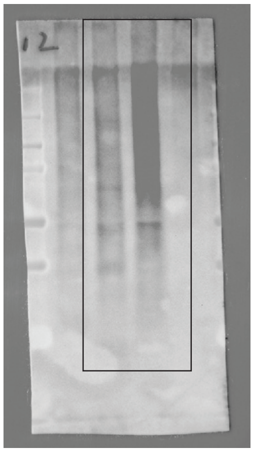

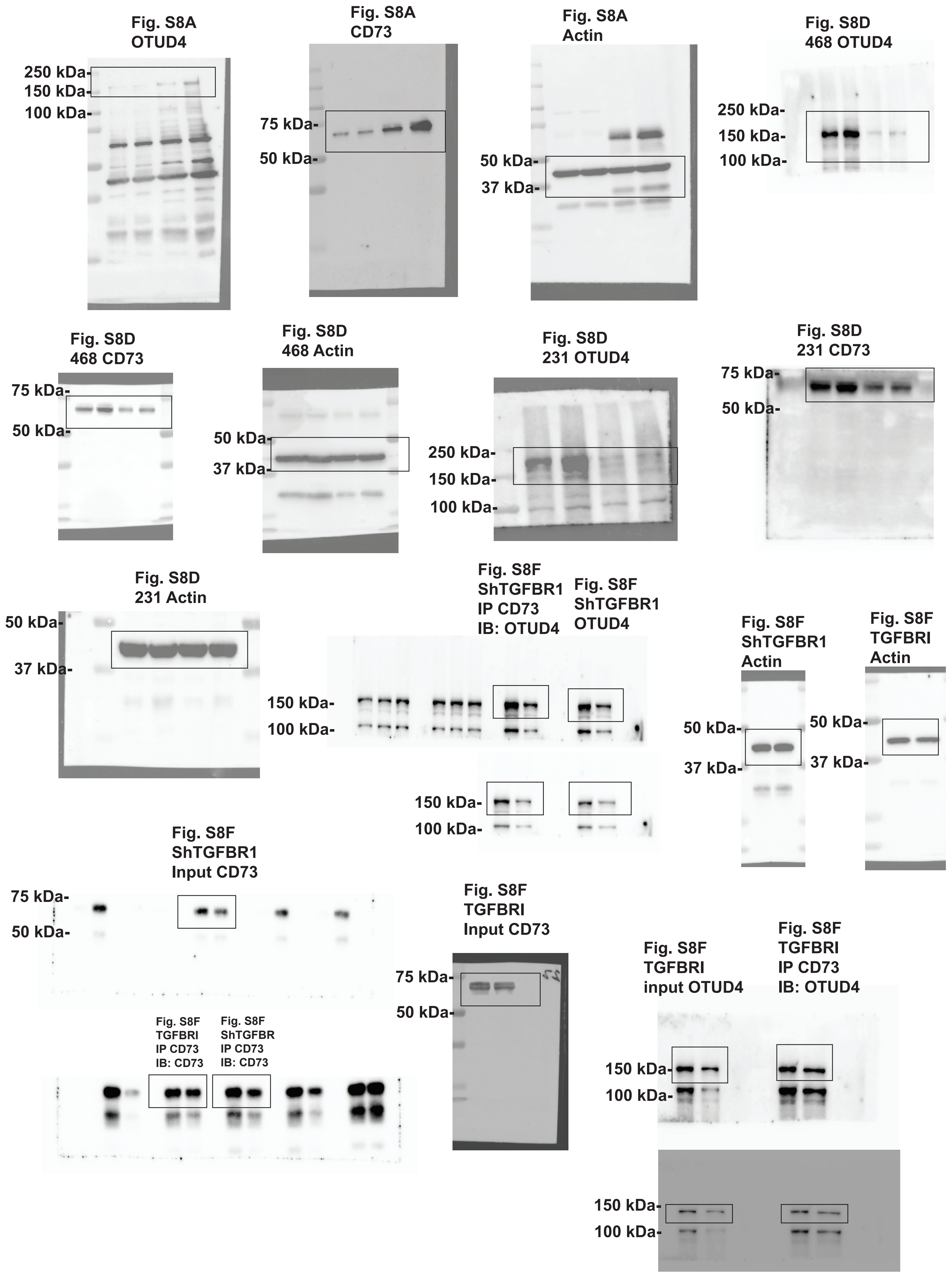

Fig. S8A OTUD4
250 kDa-
150 kDa-
100 kDa-
Fig. S8A CD73
75 kDa-
50 kDa-
Fig. S8A Actin
50 kDa-
37 kDa-
Fig. S8D 468 OTUD4
250 kDa-
150 kDa-
100 kDa-
Fig. S8D 468 CD73
75 kDa-
50 kDa-
Fig. S8D 468 Actin
50 kDa-
37 kDa-
Fig. S8D 231 OTUD4
250 kDa-
150 kDa-
100 kDa-
Fig. S8D 231 CD73
75 kDa-
50 kDa-
Fig. S8D 231 Actin
50 kDa-
37 kDa-
Fig. S8F ShTGFBR1 IP CD73 IB: OTUD4
Fig. S8F ShTGFBR1 OTUD4
150 kDa-
100 kDa-
150 kDa-
100 kDa-
Fig. S8F ShTGFBR1 Actin
50 kDa-
37 kDa-
Fig. S8F TGFBRI Actin
50 kDa-
37 kDa-
Fig. S8F ShTGFBR1 Input CD73
75 kDa-
50 kDa-
Fig. S8F TGFBRI IP CD73 IB: CD73
Fig. S8F ShTGFBR IP CD73 IB: CD73
Fig. S8F TGFBRI Input CD73
75 kDa-
50 kDa-
Fig. S8F TGFBRI input OTUD4
Fig. S8F TGFBRI IP CD73 IB: OTUD4
150 kDa-
100 kDa-
150 kDa-
100 kDa-

Fig. S10A
CD73

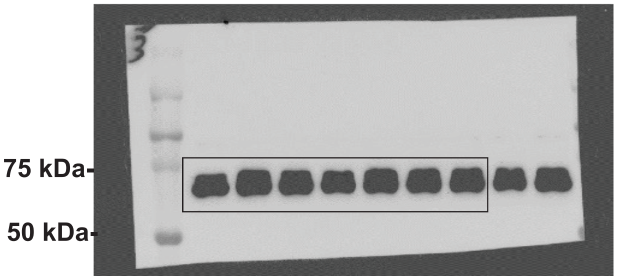

Fig. S10A
Actin

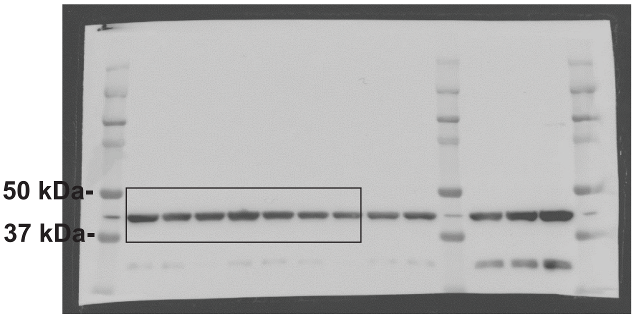

Fig. S10B
CD73

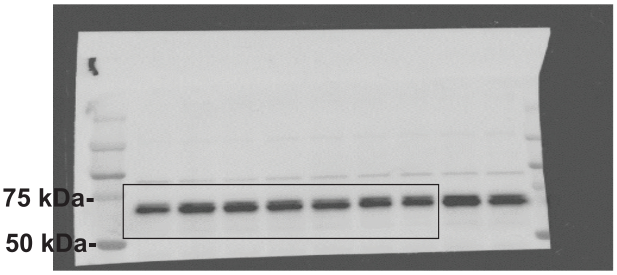

Fig. S10B
Actin

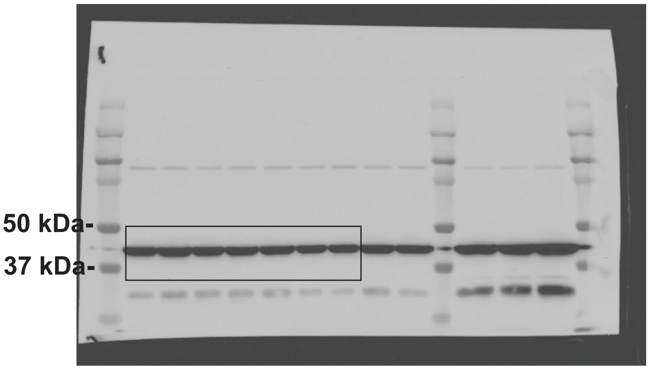

Fig. S10E
Upper CD73

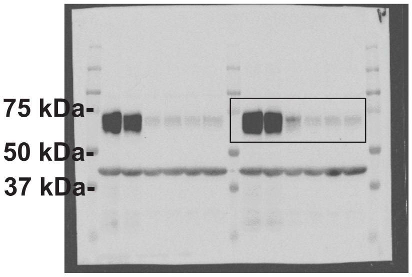

Fig. S10E
Upper actin

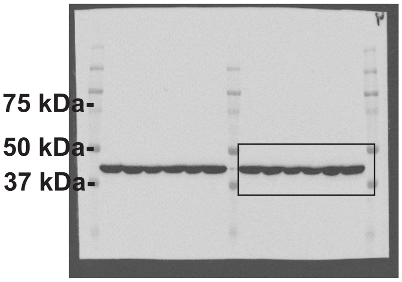

Fig. S10E
Lower CD73

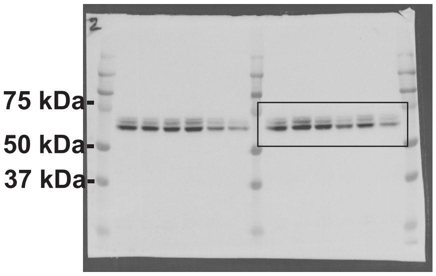

Fig. S10E
Lower actin

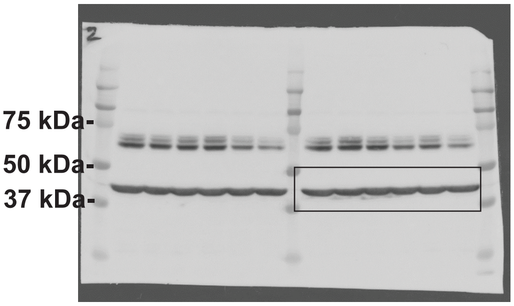

Fig. S10F upper CD73 Fig. S10F lower CD73

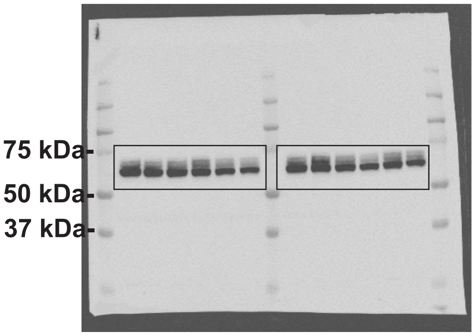

Fig. S10F upper Actin Fig. S10F lower Actin

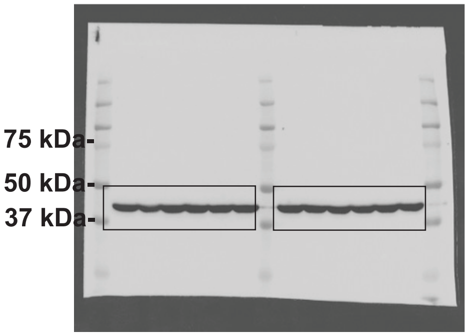

Fig. S10G upper CD73 Fig. S10G lower CD73

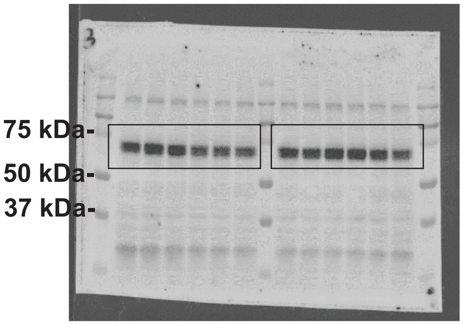

Fig. S10G upper Actin Fig. S10G lower Actin

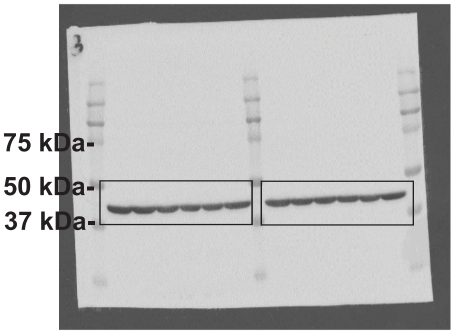

Fig. S10H upper CD73

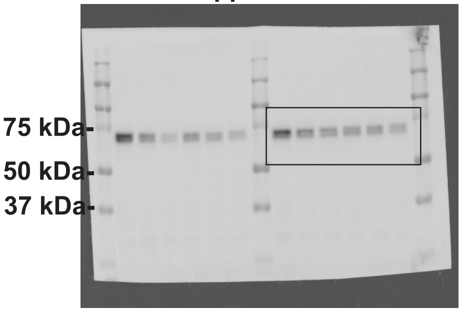

Fig. S10H upper Actin

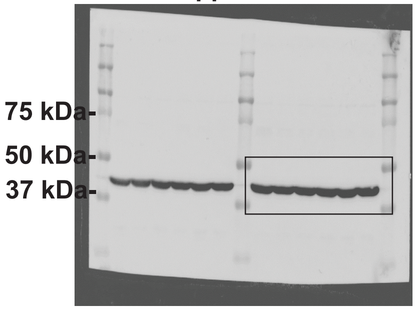

Fig. S10H lower CD73

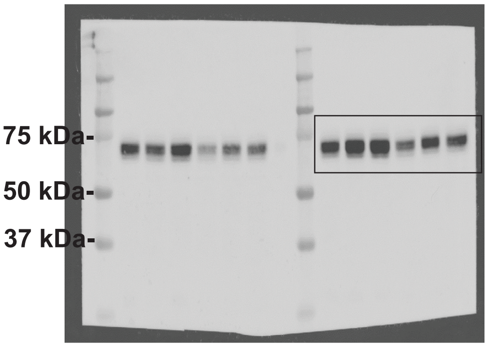

Fig. S10H lower Actin

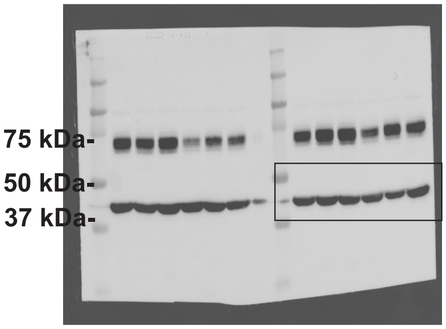

Fig. S11A CD73

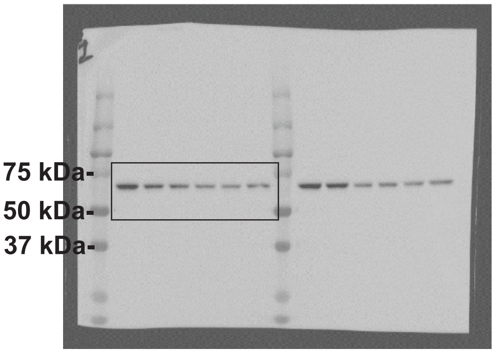

Fig. S11A Actin

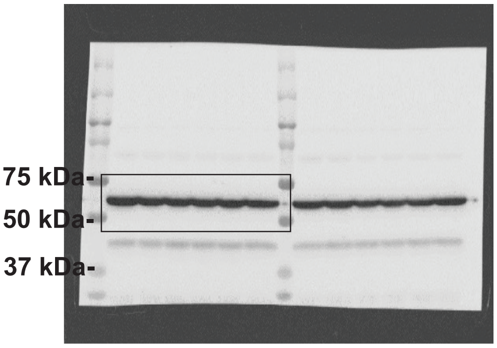

Supplement: Unedited blot and gel images [file jci-134-176390-s094.pdf]
